# Environmental Pollutants and Protein Destabilization in Lung Cancer: Anticancer Drug Strategies for Structural Stability

**DOI:** 10.1021/acsomega.5c06228

**Published:** 2026-01-01

**Authors:** Reza Rasoolzadeh, Homa Faraji, Leonardo Baptista, Fahimeh Sadat Vajedi, Vahid Nikoofard, Luciano T. Costa, José Walkimar de M. Carneiro

**Affiliations:** † Department of Inorganic Chemistry, Institute of Chemistry, Fluminense Federal University, Niterói, Rio de Janeiro 24020-141, Brazil; ‡ Biosensor Research Center, Endocrinology and Metabolism Molecular-Cellular Sciences Institute, 48439 Tehran University of Medical Sciences, Chamran Highway, Jalal-Al-Ahmad Street, Tehran 1411713137, Iran; § SpentaGen Computational Biology and AI Group, Tehran 1411713137, Iran; ∥ Department of Chemistry and Environmental, Faculty of Technology, Rio de Janeiro State University, Resende, Rio de Janeiro 27537-000, Brazil; ⊥ Department of Chemistry, Institute of Chemistry, Rio de Janeiro State University, Rio de Janeiro, Rio de Janeiro 20550-900, Brazil; # Department of Mathematics, Physics and Computation, Faculty of Technology, Rio de Janeiro State University, Resende, Rio de Janeiro 27537-000, Brazil; ¶ Institute of Chemistry, Fluminense Federal University, Campus Valonguinho, Centro, Niterói, Rio de Janeiro CEP 24020-141, Brazil

## Abstract

Environmental pollutants such as bisphenol A (BPA) and aminoantipyrine (AAP) are increasingly recognized for their detrimental effects on human health, particularly in lung cancer progression. These pollutants can alter protein conformations and interfere with anticancer drug efficacy. Among key lung cancer biomarkers, pro-gastrin-releasing peptide (Pro-GRP) and superoxide dismutase (SOD) play crucial roles in tumor progression and oxidative stress regulation, respectively. However, the molecular mechanisms underlying pollutant-induced disruptions in these proteins remain poorly understood. In this study, we employed molecular dynamics (MD) simulations, molecular docking, and binding free energy calculations to investigate the effects of BPA and AAP on the structural dynamics of Pro-GRP and SOD. Additionally, we assessed the impact of two anticancer drugs, Paclitaxel and Sotorasib, in mitigating pollutant-induced conformational instability. Our results suggest that AAP induces significant destabilization in both proteins, while BPA exhibits a milder effect. RMSD and RMSF analyses reveal that both Paclitaxel and Sotorasib stabilize protein structures by reducing fluctuations and preserving their native conformations. MM/PBSA analysis further indicates that anticancer agents modulate pollutant binding at critical residues, potentially mitigating their destabilizing effects. These findings provide computational evidence of pollutant–drug–protein interplay and suggest that environmental exposures could influence drug–protein interactions in lung cancer. As this work is computational and hypothesis-generating, experimental validation will be essential to establish biological and clinical relevance.

## Graphical abstract

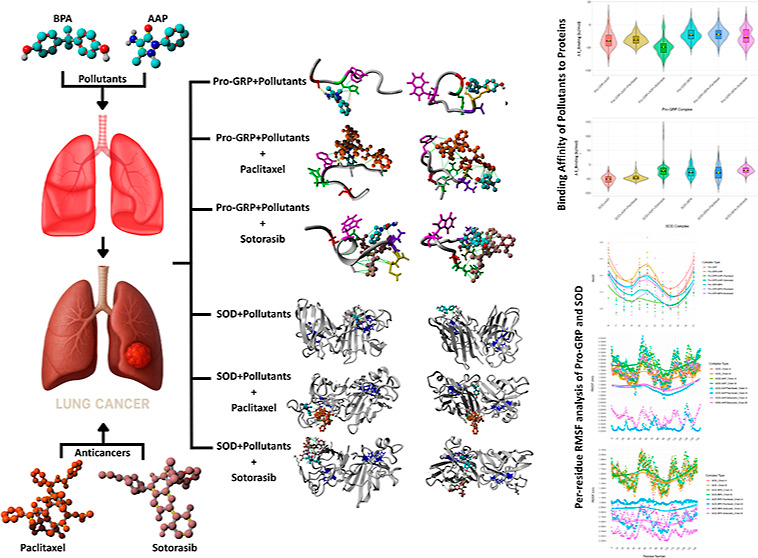

## Introduction

Lung cancer remains a leading cause of cancer-related mortality worldwide, with environmental pollutants playing a critical role in its etiology.^1,2^ Exposure to airborne pollutants, including polycyclic aromatic compounds (PACs), nitro-PACs, bisphenols (BPA, BPS), and environmental contaminants such as aminoantipyrine (AAP), has been linked to oxidative stress, inflammation, and direct molecular alterations that promote carcinogenesis.^3–5^ Notably, bisphenol A (BPA) has been implicated in lung cancer progression by upregulating matrix metalloproteinases via the G protein-coupled estrogen receptor (GPER)/ epidermal growth factor receptor (EGFR)/ extracellular signal-regulated kinase 1/2 (ERK1/2) signaling pathway.^2^

Among the many environmental pollutants implicated in lung carcinogenesis, **AAP** and **BPA** were selected for this study as representative examples of two major chemical classes aromatic amines and phenolic endocrine disruptors. **AAP** has been detected in industrial and pharmaceutical effluents and exhibits redox-active behavior capable of modifying protein side chains, reflecting its reported interactions with biological peptides and nanoparticles.^5^ **BPA**, one of the most prevalent endocrine disruptors, has been repeatedly identified in human plasma and lung tissue and is mechanistically linked to oxidative stress pathways and redox imbalance.^6,7^ These compounds therefore capture both electrophilic and phenolic modes of protein interaction observed across broader pollutant categories such as PAHs and nitro-PAHs.^4^

Biomonitoring studies indicate that human exposure to BPA is widespread, occurring mainly through dietary intake from food packaging, but also through dermal absorption and inhalation of airborne particles and indoor dust with unconjugated (bioactive) BPA consistently detected in human blood in the nanogram per milliliter range. Its presence has been reported in a variety of human biological fluids, including urine, blood, breast milk, and amniotic fluid, highlighting its clinical relevance.^6,7^ While large-scale human biomonitoring data for AAP are currently limited, its selection for this study is based on its status as a widely recognized environmental pollutant, which justifies its investigation in the context of human health. Understanding the molecular interactions between these pollutants and key lung cancer-related proteins is crucial to elucidating their role in carcinogenesis and their potential interference with anticancer therapies.

Among lung cancer biomarkers, proteins such as pro-gastrin-releasing peptide (Pro-GRP) and superoxide dismutase (SOD) have been extensively studied. Pro-GRP, a biomarker for small-cell lung cancer (SCLC), is highly expressed in tumor tissues and has a diagnostic sensitivity of approximately 84% and specificity of 95%.^8^ Elevated plasma levels of Pro-GRP are observed in SCLC patients, making it a reliable marker for early detection and monitoring.^9^ Additionally, antioxidant proteins, including SOD, glutathione peroxidase (GPx), catalase (CAT), and glutathione reductase (GR), play crucial roles in mitigating oxidative stress, which is a hallmark of lung cancer pathophysiology.^10^

SOD represents a central antioxidant enzyme that converts superoxide radicals into hydrogen peroxide and oxygen, thereby maintaining redox homeostasis.^10^ Dysregulated SOD activity is frequently observed in lung tumors and contributes to oxidative-stress imbalance and therapy resistance.^10,41^

Consequently, interactions between these proteins and environmental pollutants may influence not only tumor progression but also the efficacy of anticancer therapies.

Given the increasing relevance of targeted therapies, we also explored the interactions of lung cancer drugs with these proteins. Sotorasib, a Kirsten rat sarcoma viral oncogene homologue (KRAS)-G12C inhibitor, has demonstrated efficacy in nonsmall cell lung cancer (NSCLC) by selectively targeting mutant KRAS.^11,12^ Microtubule-stabilizing agents like docetaxel and Paclitaxel, are standard chemotherapeutics for lung cancer treatment, exerting their effects by disrupting mitotic spindle formation and inducing apoptosis.^13^ However, environmental pollutants may interfere with the binding and efficacy of these drugs, potentially altering their therapeutic potential.

To address these concerns, we employed molecular docking and molecular dynamics (MD) simulations to investigate the interactions between pollutants (AAP and BPA) and key lung cancer-associated proteins (Pro-GRP and SOD). Furthermore, we examined how these pollutants modulate the interactions between Pro-GRP and SOD with anticancer drugs, including Sotorasib and Paclitaxel. By elucidating these molecular interactions, our study aims to provide insights into the potential impact of environmental pollutants on lung cancer progression and therapeutic response, paving the way for more effective treatment strategies.

## Methods

In this study, the effects of pollutants on two key proteins associated with lung cancer, Pro-GRP and SOD, were investigated. Specifically, their interactions with two pollutants, AAP and BPA, as well as with two anticancer drugs, Sotorasib and Paclitaxel, were analyzed. A total of 54 μs of molecular dynamics simulations (MDS), including 1000 ns MDS with three repetitions for each complex, were performed. The models were analyzed based on several key metrics, including Root Mean Square Deviation (RMSD), Root Mean Square Fluctuation (RMSF), total energy, ligand–receptor distance, conformational changes, radius of gyration (Rg), and binding affinity (using MM/PBSA).

### Protein Preparation and Molecular Docking Simulation

The chosen pollutants (AAP, BPA) and drugs (Paclitaxel, Sotorasib) were selected to represent chemically and mechanistically distinct prototypes relevant to lung-cancer biology. AAP (an aromatic amine) and BPA (a phenolic compound) differ in polarity, aromaticity, and hydrogen-bonding patterns, enabling comparison of two major environmental toxicant classes.^4,5^ Paclitaxel (a microtubule stabilizer) and Sotorasib (a KRAS-G12C inhibitor) represent standard chemotherapeutic and targeted approaches, respectively, providing insight into different therapeutic mechanisms that could counteract pollutant-induced destabilization.^11–13^

The 3D crystal structures of Pro-GRP (PDB ID: 7W3Z) and SOD (PDB ID: 5YTO) were obtained from the RCSB Protein Data Bank (PDB)^14–16^ and subsequently processed using YASARA-View and AutoDock Tools.^17,18^

For Pro-GRP, since 7W3Z represents the complete structure of the human Gastrin-Releasing Peptide Receptor in complex with the agonist Gastrin-Releasing Peptide and Gq heterotrimers, we extracted the Pro-GRP portion (with the sequence PRGNHWAVGHLM).

For SOD, all crystal structures contain an asymmetric unit with five biologically relevant dimers. All water molecules were removed, and only chains A and B were selected and separated.

Subsequently, we performed energy minimization of the structures using the YASARA server21 and validated its structure using Procheck 22. All residues were found in the most favored and allowed regions (Figure S1).

To obtain the structures of anticancer drugs and pollutants, we used PubChem (accessed March 4, 2024). The selected compounds were Sotorasib (CID: 137278711), Paclitaxel (CID: 36314), AAP (CID: 2151), and BPA (CID: 6623).

Docking simulations were performed using AutoDock Vina.^19^ For each ligand (and for ternary complexes, the second ligand), 25 independent docking runs were carried out. The generated poses were clustered, and the top-ranked binding modes were selected based on binding free energy (Δ*G*) and cluster consistency (Table S1).

For all docking simulations, MD-relaxed protein structures were employed to better approximate near-physiological conformations. For ternary complexes denoted [Protein + Ligand1]+Ligand2, the second ligand was subsequently docked onto the MD-relaxed binary complex, thereby capturing potential cooperative or competitive effects between ligands.

### MD Simulations

MD simulations were conducted at a temperature of 310 K using GROMACS 2024 software with the Amber99SB force field.^20,21^ The parameters for the anticancer drugs were obtained from DrugBank.^22^ The physiological charge of Sotorasib is −1, while that of Paclitaxel is 0. We meticulously integrated the additional ligand atoms into the complex’s topology files, ensuring that all parameters for these ligands were properly incorporated.

Ligand topologies and parameters were generated using the ACPYPE tool (AnteChamber PYthon Parser interfacE), which relies on Antechamber to generate AMBER-compatible files. The General AMBER Force Field (GAFF) was employed for all ligands, and partial atomic charges were assigned using the AM1-BCC method, a semiempirical approach that combines AM1 with bond charge corrections to provide accurate electrostatic representations for small molecules.^22^

The free proteins and complexes were placed in a cubic box with dimensions of 9 nm, achieved using the gmx editconf module to set the boundary conditions. The protein was solvated using the Transferable Intermolecular Potential with 3 Points (TIP3P) solvent model. The TIP3P model was selected due to its efficient balance between computational speed and accuracy in reproducing key bulk water properties, such as density, radial distribution functions, and diffusion coefficients. TIP3P remains a widely used model in biomolecular simulations for its compatibility with force fields like Amber and CHARMM, offering a reasonable approximation of water’s physical properties.^23^ To neutralize the system and maintain a physiological ionic strength (0.15 M NaCl), Na^+^ and Cl^–^ ions were added to the free proteins and their complexes using the gmx genion module. All systems were energy-minimized with 1500 steps of the steepest descent algorithm.^24^ During the subsequent equilibration phase, a two-step approach was employed:

1. NVT Equilibration:^25,26^ The system was first equilibrated under constant volume and temperature (*NVT* ensemble) for 500 ps, using the modified Berendsen thermostat (V-rescale) to gradually increase the temperature from 0 to 310 K.
2. NPT Equilibration:^25,27^ The system was then equilibrated under constant pressure and temperature (*NPT* ensemble) for an additional 500 ps, using the Parrinello–Rahman barostat to maintain a pressure of 1 bar.

Following equilibration, 1000 ns production MD simulations were performed under NPT conditions, and the resultant trajectories were analyzed using various GROMACS utilities. Details of each system’s MD simulation are presented in Table S2.

### Binding Free Energy Calculations

The binding free energy of protein-pollutant and pollutant–drug complexes was estimated using the Molecular Mechanics/Poisson–Boltzmann Surface Area (MM/PBSA) method.^28^ This approach provides a balance between accuracy and computational efficiency, making it widely used in biomolecular simulations. MM/PBSA calculations were performed on equilibrated trajectories obtained from MDS. The total binding free energy (Δ*G*_bind) was computed as the sum of molecular mechanics energy (electrostatic and van der Waals (vdW) interactions), solvation free energy (polar and nonpolar contributions), and entropic contributions. The polar solvation energy was estimated using the Poisson–Boltzmann (PB) model, while the nonpolar term was derived from the solvent-accessible surface area (SASA). Entropic contributions were approximated using the normal-mode analysis approach. All calculations were performed using the g_mmpbsa tool integrated within GROMACS. The binding free energy results provided insights into the stability and affinity of pollutant-protein interactions as well as the competitive binding of anticancer drugs (Sotorasib and Paclitaxel) to pollutant–bound complexes.

### Statistical Analysis

As mentioned, each MDS run was repeated three times with different initial velocities, resulting in a total simulation time of 3000 ns (1000 ns × 3) for each complex. Prior to statistical analysis, the normality of data distributions was evaluated using the Shapiro–Wilk test. Statistical analyses were then performed using Welch’s ANOVA or one-way ANOVA to assess the significance of differences among all simulated complexes. The choice between Welch’s ANOVA and one-way ANOVA was based on Levene’s test for homogeneity of variances.^29^

Based on Levene’s test results, two post hoc tests were applied for pairwise comparisons:

1. Tukey’s Honest Significant Difference (HSD) test, when variances were homogeneous, to control the family wise error rate in multiple comparisons.
2. Games-Howell post hoc test, when variances were inhomogeneous, as it does not assume equal variances or sample sizes.

This statistical approach ensured a comprehensive evaluation of differences between the reference complex (Pro-GRP and SOD) and pollutant-treated complexes, as well as among pollutant-only and drug-treated complexes, to assess the impact of drug binding on pollutant-protein interactions. In all statistical analyses, Δ represents the difference relative to our reference, calculated using differences between means. Effect sizes and confidence intervals were considered alongside p-values to provide a robust interpretation of results.

The significance level for all statistical tests was set at 0.05 (*p* < 0.05). All statistical analyses were conducted using R version 4.4.2.

Our simulations utilize implicit 1:1 binding ratios, these align with the physiological concentrations of BPA observed in human serum (0.2–20 ng/mL) and tissues (up to 100 ng/g), where even nanomolar concentrations can disrupt signaling. AAP residues, although less commonly measured in environmental studies, are detected at concentrations ranging from 0.04 to 10 μg/mL in blood due to exposure, which corroborates our observations of destabilization at similar affinities. Increased levels (for example, in groups with high exposure) may amplify effects, thereby highlighting the need for studies related to biomonitoring.^30–32^

## Results and Discussion

### RMSD Analysis Reveals Drug-Induced Stabilization of Pro-GRP and SOD Proteins in the Presence of Pollutants

The C-alpha RMSD analysis provides crucial insights into the structural dynamics of the Pro-GRP and SOD complexes, as presented in Figures 1 and S2. These violin plots and time-series data display the RMSD distributions and fluctuations, with all statistically significant differences annotated in Table S3.

**Figure 1:**
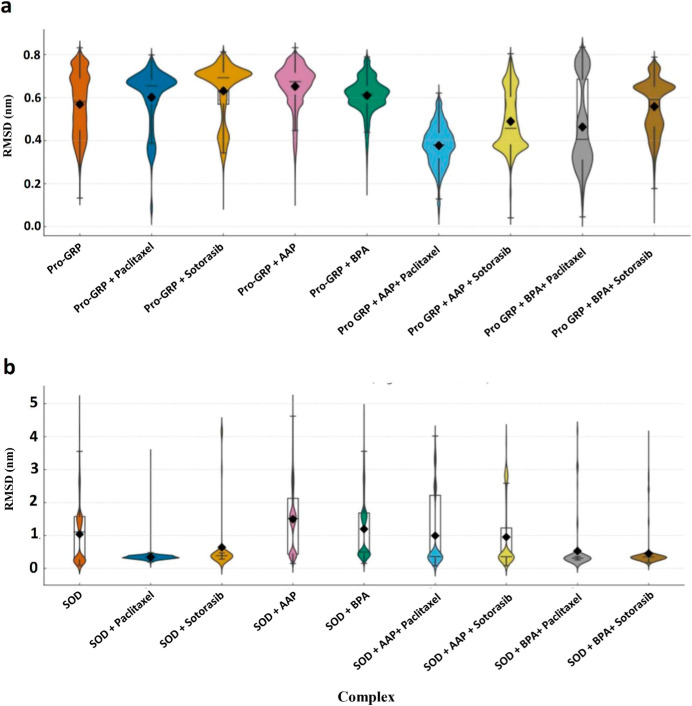
RMSD distribution of molecular dynamics simulations for Pro-GRP and SOD complexes. (a) RMSD profiles of Pro-GRP complexes with pollutants (AAP, BPA) and anticancer drugs (Paclitaxel, Sotorasib). The addition of anticancer drugs, particularly Paclitaxel, after pollutant binding significantly reduced RMSD values, indicating enhanced structural stability. (b) RMSD profiles of SOD complexes under similar conditions, showing greater fluctuations, especially in the presence of AAP, suggesting increased structural flexibility. Paclitaxel consistently reduced RMSD, reinforcing its strong stabilizing effect on the SOD complex. Yellow diamonds represent the mean RMSD values for each complex.

The analysis of Pro-GRP complexes reveals a nuanced response to molecular interactions. As expected, the addition of pollutants (Pro-GRP + AAP, Pro-GRP + BPA) leads to increased fluctuations and higher mean RMSD values compared to the Pro-GRP reference complex (*p* < 0.0001), highlighting pollutant-induced destabilization. In a distinct pattern, the addition of anticancer drugs alone (Pro-GRP + Paclitaxel, Pro-GRP + Sotorasib) also results in a slight but statistically significant increase in RMSD compared to the reference (*p* < 0.0001). This indicates that for Pro-GRP, both pollutants and drugs induce some conformational changes. However, when combined with pollutants, the drugs demonstrate a strong mitigating influence. When compared directly to the Pro-GRP + AAP complex, the Pro-GRP + AAP + Paclitaxel and Pro-GRP + AAP + Sotorasib complexes show a significant reduction in RMSD (*p* < 0.0001). A similar mitigating effect is observed for the Pro-GRP + BPA complex, where the addition of either Paclitaxel or Sotorasib significantly reduces RMSD compared to the pollutant-only complex (*p* < 0.0001).

A different and more pronounced trend is observed in the SOD complexes. The SOD + AAP and SOD + BPA complexes show a significant increase in RMSD compared to the SOD reference, confirming their destabilizing effect (*p* < 0.0001). In a critical contrast to Pro-GRP, the addition of Paclitaxel or Sotorasib alone (SOD + Paclitaxel, SOD + Sotorasib) results in a notably lower mean RMSD than the reference SOD complex (*p* < 0.0001). This demonstrates that both drugs are highly effective at enhancing the structural stability of the native SOD protein. This inherent stabilizing capacity is further highlighted when the drugs are combined with pollutants. The addition of Paclitaxel or Sotorasib to the SOD + AAP complex results in a dramatic reduction in RMSD, significantly counteracting the pollutant’s destabilizing effect (*p* < 0.0001). Similarly, for the SOD + BPA complexes, the addition of either drug restores stability to a level below that of the native SOD, showcasing their powerful stabilizing and mitigating effects.

From a biological perspective, the pollutant-induced destabilization of Pro-GRP may alter its ability to bind and activate GRPR, potentially disrupting downstream ERK-mediated proliferative signaling in SCLC.^2,8,9^

Similarly, conformational destabilization of SOD could impair its catalytic efficiency in dismutating reactive oxygen species, thereby aggravating oxidative stress a hallmark of lung-cancer pathophysiology.^10,44^

This comprehensive analysis confirms that while environmental pollutants induce destabilization in both proteins, the effect of anticancer drugs is protein-specific. For Pro-GRP, they primarily act as mitigators of pollutant effects, while for SOD, they are inherently stabilizing and show a powerful restorative effect in the presence of pollutants. This provides a more nuanced and scientifically rigorous understanding of their function.

Abo-Zaid et al. found that BPA administration had a deleterious effect by exacerbating oxidative stress through the inhibition of antioxidant enzyme activities, specifically SOD and GSPx, in the respiratory system of adult female rats.^33^ Based on our findings, one possible reason for this reduced activity could be the destabilization of SOD upon exposure to BPA.

It has been found that increased reactive oxygen species (ROS) generation is a hallmark of acute lung injury induced by BPA exposure, and several models have demonstrated that its inhibition can protect the lungs from BPA-induced damage.^34^ Therefore, the stabilizing effect of anticancer drugs, particularly when administered after pollutant exposure, suggests a promising therapeutic strategy for mitigating pollutant-induced protein destabilization.^34–37^

### Thermodynamic Stability Analysis of Pollutant-Protein and Drug–Protein Complexes

To investigate the stability of these complexes, total energy calculations were performed to provide a thermodynamic perspective on stability (Figure 2). All pairwise comparisons were highly significant (*p* < 0.001), further supporting these conclusions (Table S4).

**Figure 2:**
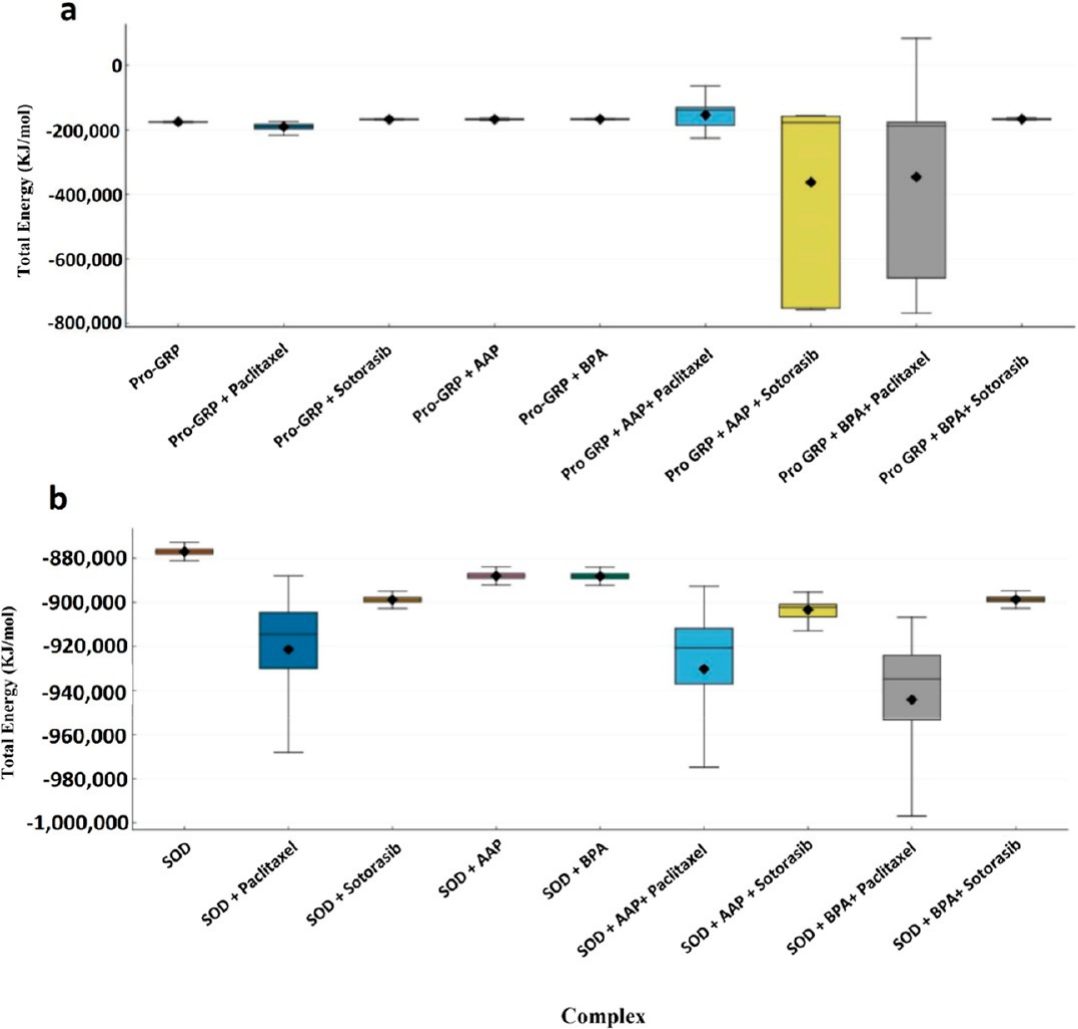
Total Energy Distribution of Pro-GRP and SOD Complexes. Box plots representing the total energy (kJ/mol) of different Pro-GRP (a) and SOD (b) complexes obtained from MDS. Higher total energy values in Pro-GRP complexes, particularly for AAP- and BPA-bound structures, indicate destabilization, while the addition of Paclitaxel and Sotorasib modulates stability in a pollutant-dependent manner. In contrast, for SOD complexes, exposure to pollutants significantly reduces total energy, suggesting increased structural stability. Paclitaxel consistently lowers total energy across both systems, while Sotorasib has a less pronounced but still stabilizing effect. Error bars represent standard deviations, and black diamonds indicate mean values.

For Pro-GRP, AAP- and BPA-bound complexes exhibited significantly higher total energy values compared to the Pro-GRP reference, suggesting that pollutants cause both structural destabilization (based on C-alpha RMSD) and contribute to the overall thermodynamic instability of the system. The addition of anticancer drugs alone also led to significantly different total energy values than the Pro-GRP reference. While the addition of Paclitaxel reduced the total energy, Sotorasib, in contrast, increased it. The combined effects are also pollutant-specific. While the total energy of Pro-GRP + AAP + Paclitaxel is higher than Pro-GRP + AAP, it effectively reduces total energy in the presence of BPA. Similarly, Sotorasib decreases total energy when exposed to AAP, but Pro-GRP + BPA + Sotorasib has a higher total energy than Pro-GRP + BPA, indicating a different effect based on the pollutant.

For SOD, exposure to both pollutants significantly reduced the total energy of the whole system, suggesting increased complex stability upon pollutant binding. However, this stabilization could have negative implications, as it indicates that these complexes remain stably bound to the pollutants, potentially altering their biological function. The addition of Paclitaxel or Sotorasib alone also significantly reduced the total energy of the SOD complex, an effect that was even more pronounced than with the pollutants. This mirrors the stabilizing effect observed in the RMSD analysis. When the drugs are combined with pollutants, they consistently further reduce the total energy of the system, showcasing their strong stabilizing capacity. This supports the notion that conjugating SOD with stabilizing elements enhances both its structural stability and enzymatic activity.^38^

### Residue-Specific Flexibility Analysis

To further examine the local flexibility of Pro-GRP and SOD complexes, we performed RMSF analysis, which provides residue-specific insight into structural stability. The results, presented in Figure 3, highlight regions of enhanced flexibility and their modulation by pollutant exposure and anticancer drug incorporation.

**Figure 3:**
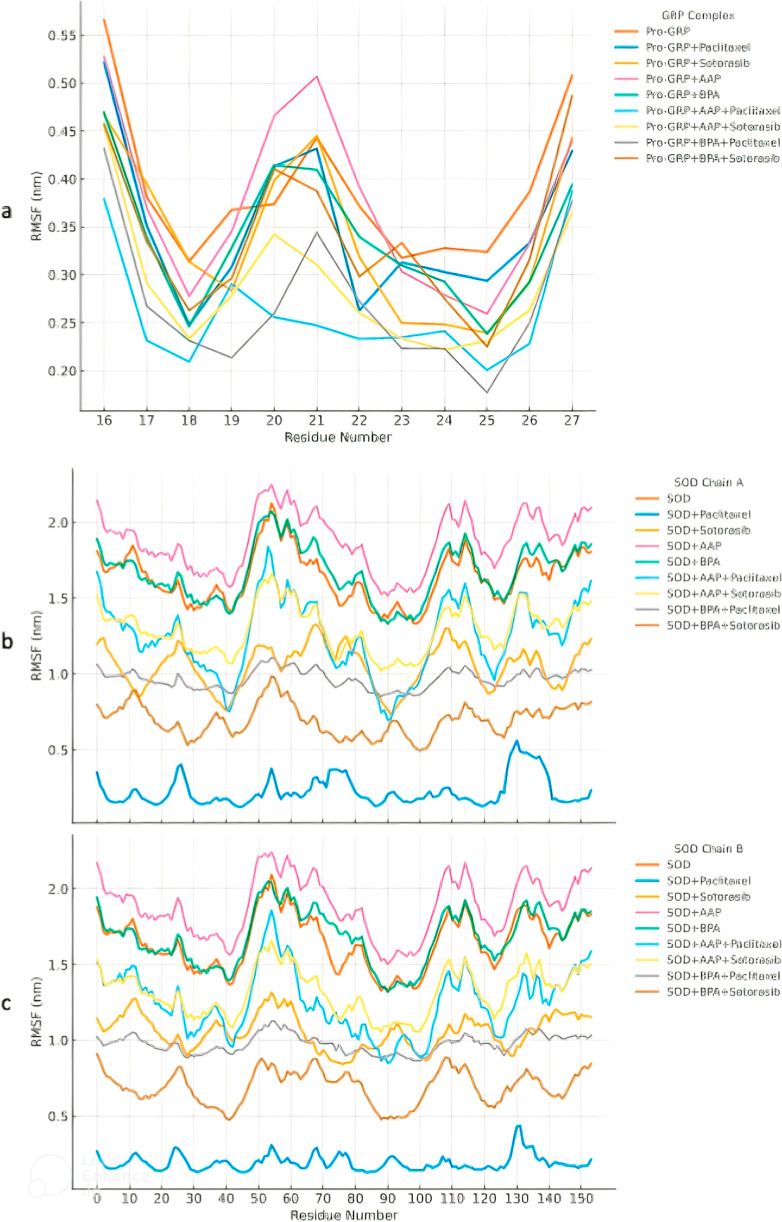
Per-residue RMSF analysis of Pro-GRP and SOD complexes. (a) RMSF profiles of Pro-GRP complexes indicate that the highest fluctuations occur in the N-terminal, C-terminal, and middle regions, particularly around Trp21, a crucial residue for GRP receptor activation. AAP exposure increases flexibility in the middle regions, though the changes are not statistically significant. In contrast, both Paclitaxel and Sotorasib significantly reduce RMSF compared to BPA-exposed GRP, suggesting stabilization. RMSF profiles of SOD complexes reveal three distinct fluctuation regions. AAP (b) exposure significantly increases overall RMSF (*p* < 0.001), while BPA (c) exposure does not induce notable fluctuations. Both anticancer drugs significantly reduce RMSF compared to pollutant-exposed complexes (*p* < 0.001), particularly affecting functionally critical residues such as His46, His48, His63, and His120, which coordinate metal ions essential for enzymatic activity. The scatter points represent individual data values, while the lines illustrate the smoothed trend for each condition.

In all Pro-GRP complexes, the most fluctuating residues were observed in the N-terminal, C-terminal, and middle regions. Notably, Trp21, which was previously identified as a key hydrophobic core residue in activated GRP, plays a crucial role in GRP receptor helical conformation stability and is critical for Gq signaling.^14^ Located in the middle of the peptide, Trp21 falls within a highly fluctuating region.

The RMSF profiles of Pro-GRP indicate an increase in fluctuation upon AAP exposure in the middle flexible regions, especially for Trp21. However, as shown in Table S5, this increase is not statistically significant (*p* > 0.05), and the introduction of BPA also did not result in significant differences compared to the control (*p* > 0.05).

The analysis of Pro-GRP complexes reveals a nuanced response to molecular interactions. As expected, the addition of pollutants (Pro-GRP + AAP, Pro-GRP + BPA) leads to increased fluctuations and higher mean RMSF values in the middle flexible regions, particularly around Trp21. However, as shown in Table S5, the RMSF profiles of Pro-GRP + AAP and Pro-GRP + BPA did not show statistically significant differences when compared to the Pro-GRP reference complex (*p* > 0.05). The same lack of statistical significance was observed for the anticancer drugs alone; the RMSF values for Pro-GRP + Paclitaxel and Pro-GRP + Sotorasib were not significantly different from the Pro-GRP control (*p* > 0.05). Despite this, when combined with pollutants, a mitigating influence was observed. For example, Paclitaxel significantly decreased RMSF compared to the Pro-GRP + BPA complex (*p* < 0.05), indicating a clear reduction in pollutant-induced flexibility. The effect of Sotorasib in this context was not statistically significant.

The RMSF patterns of SOD complexes differed from those of Pro-GRP. While AAP significantly increased overall RMSF (*p* < 0.001) in both chains, the fluctuations in BPA-exposed residues were not statistically significant (*p* > 0.05). Both anticancer drugs exhibited significantly lower RMSF values than pollutant-exposed complexes (*p* < 0.001). However, it should be noted that this decrease in enzyme flexibility is particularly high, especially in functionally important regions. These regions exhibit significantly lower flexibility compared to the normal enzyme (nonexposed SOD), which may impact its function. This could be one of the reasons for Sotorasib’s side effects which clinical data indicate that treatment dosage must be carefully managed due to its adverse effects.^39^

We observed three fluctuating regions in both chains: a peak between residues 40 and 80, a valley between residues 80 and 120, and a rising RMSF trend in the C-terminal region. Notably, all four catalytic residues (His46, His48, His63, and His120) of SOD, which coordinate metal ions crucial for enzymatic activity and whose stability is essential,^40–42^ are located in the most fluctuating RMSF regions. Consequently, the protein’s function is likely impacted.

Statistical analysis of these four critical residues indicates that conditions involving BPA and anticancer drug treatments exhibit significantly lower RMSF values compared to other conditions. These moderate but significant differences (*p* < 0.05) between SOD + BPA + Paclitaxel and SOD + BPA + Sotorasib suggest that Paclitaxel and Sotorasib impact RMSF differently, likely due to their distinct molecular interactions with the protein.

### Impact of Pollutants and Anticancer Drugs on Protein Conformational Stability

Statistical analysis of the Pro-GRP complexes reveals that while some comparisons show minor but significant conformational changes, the overall effect is limited. A heatmap was generated to provide a visual representation of Pro-GRP residue flexibility across different complexes, illustrating how pollutants (AAP and BPA) and anticancer drugs (Paclitaxel and Sotorasib) influence the protein’s conformational dynamics (Figure 4a).

**Figure 4:**
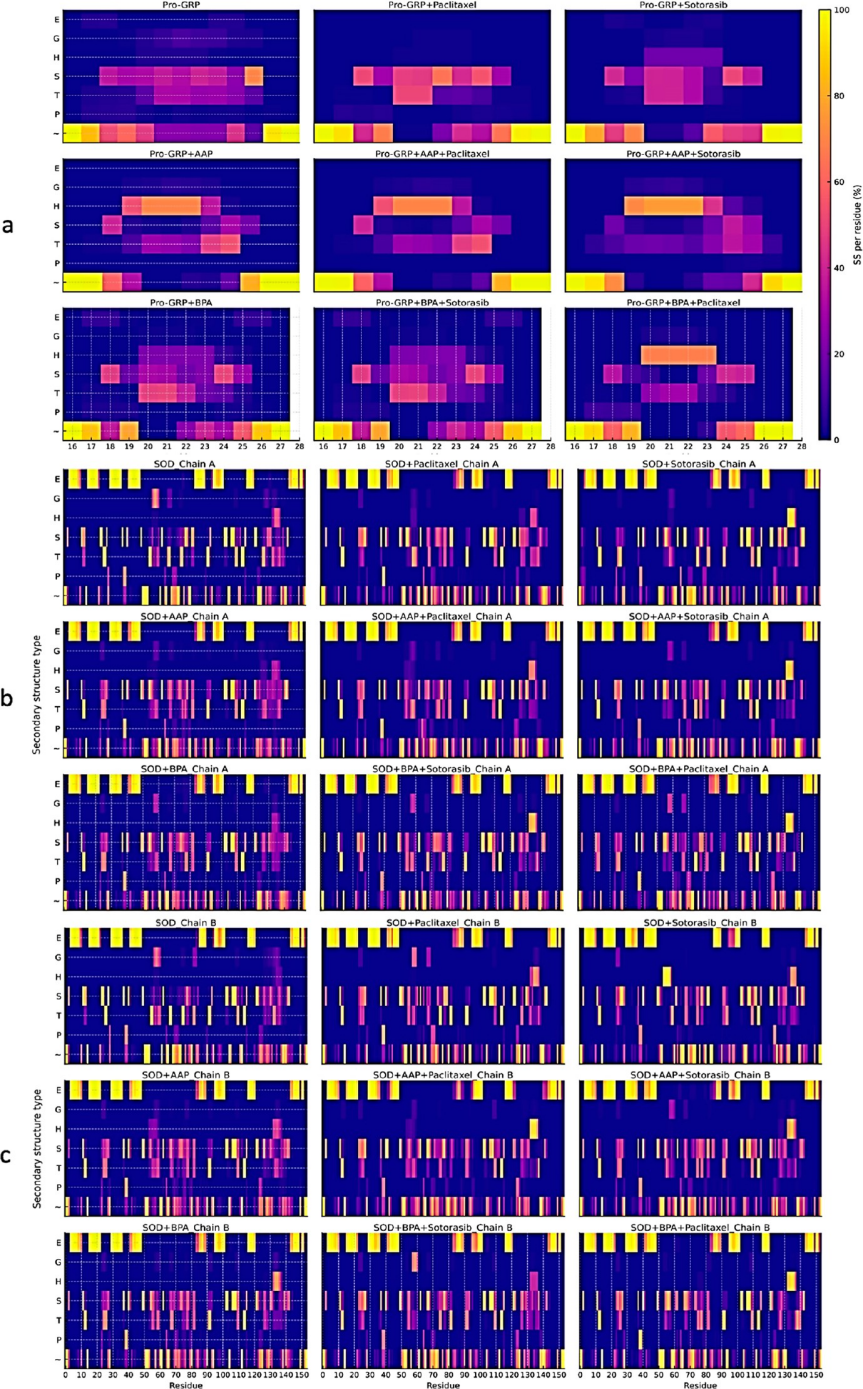
Heatmap representation of secondary structure persistence during MDS. This heatmap (a): Pro-GRP, ((b): SOD_chain A, (c): SOD_chain B) illustrates the percentage of time each residue maintained a specific secondary structure throughout MDS. Yellow regions indicate residues that consistently retained a single structural conformation (high persistence), while blue regions signify residues with low or no occupancy of that structure, indicating higher structural transitions and flexibility. In case of Pro-GRP, an interesting trend is observed upon AAP binding, particularly in the residue range of 19 to 23, where a reduction in extended β-strands (E) and isolated β-bridge (B) residues is accompanied by an increase in α-helix (H) content. This structural shift leads to reduced compactness, which is consistent with our gyration radius (Rg) results (Figure 8).

In the case of SOD, in agreement with the RMSF analysis and chain mobility, both AAP and BPA induce notable conformational changes in both chains (Figure 4b,c and Table S6). One notable observation is that although AAP affects both chain structures, the extent of these changes differs, with AAP altering the structure of chain A more than chain B (*p* < 0.001 vs *p* < 0.05). However, comparing these chain analyses together does not show any significant differences; the differences are only observed when comparing them with their reference structures (SOD). In contrast to the effect of pollutants, anticancer drugs also induce significant conformational deviations from the unexposed protein, as seen in the comparisons of drug-treated complexes with the native SOD (*p* < 0.001). Importantly, the drug-induced conformations represent distinct and stable structural states, rather than a simple restoration of the native fold. This observation is in good agreement with what we observed for total energy and RMSD (Figures 1b and 2b). This suggests that Paclitaxel and Sotorasib do not simply restore the native structure, but rather create a new, stable conformation in both the absence and presence of pollutants.

This moderation of pollutant effects is particularly relevant for preserving the enzyme’s functional integrity, as excessive conformational shifts may impair catalytic efficiency. This is in agreement with the study by Pieniążek et al., which stated that the administration of Paclitaxel increased rat liver SOD activity.^43^

A direct comparison of paclitaxel and sotorasib revealed clear differences in their ability to stabilize pollutant-exposed proteins. Across RMSD and RMSF profiles, paclitaxel consistently reduced conformational fluctuations of both Pro-GRP and SOD more effectively than sotorasib, particularly in the presence of AAP. MM/PBSA free energy calculations further supported this observation, showing stronger binding energies and more favorable stabilization with paclitaxel. While sotorasib also conferred a measurable stabilizing effect, its impact was less pronounced. These findings indicate that paclitaxel exerts a superior stabilizing influence on pollutant-exposed proteins compared to sotorasib.

The superior stabilizing action of Paclitaxel observed in our simulations may therefore have broader biological implications. By preserving the structural integrity of SOD and Pro-GRP, Paclitaxel could help maintain redox balance and signaling fidelity, counteracting pollutant-induced dysfunctions that contribute to tumor progression and variable therapeutic outcomes.^10,44^

### Impact of Anticancer Drugs on Pollutant Binding and Key Residue Interactions in GRP and SOD Complexes

As previously mentioned, since Trp21 plays a significant role in forming hydrophobic interactions and hydrogen bonds crucial for Gq signaling,^14^ we focused on this residue and selected it for indexing. This approach is expected to provide valuable insights into the interactions between pollutants and anticancer agents with GRP, as well as their impact on GRP’s interaction with its receptor (GRPR).

A comparison of the Pro-GRP + AAP complex with drug-treated complexes (Figure 5a) shows a decrease in the distance between the pollutant and the Trp21 residue upon treatment with both Paclitaxel and Sotorasib. Although this decrease is less than 1 nm, it is statistically significant (*p* < 0.001) (Table S7). To understand the reason behind this observation, we visualized their structures. As shown in Figure 6d,f,h, the Pro-GRP + AAP structure becomes more compact upon the addition of both drugs, which explains why the pollutant moves closer to Trp21 under these conditions.

**Figure 5:**
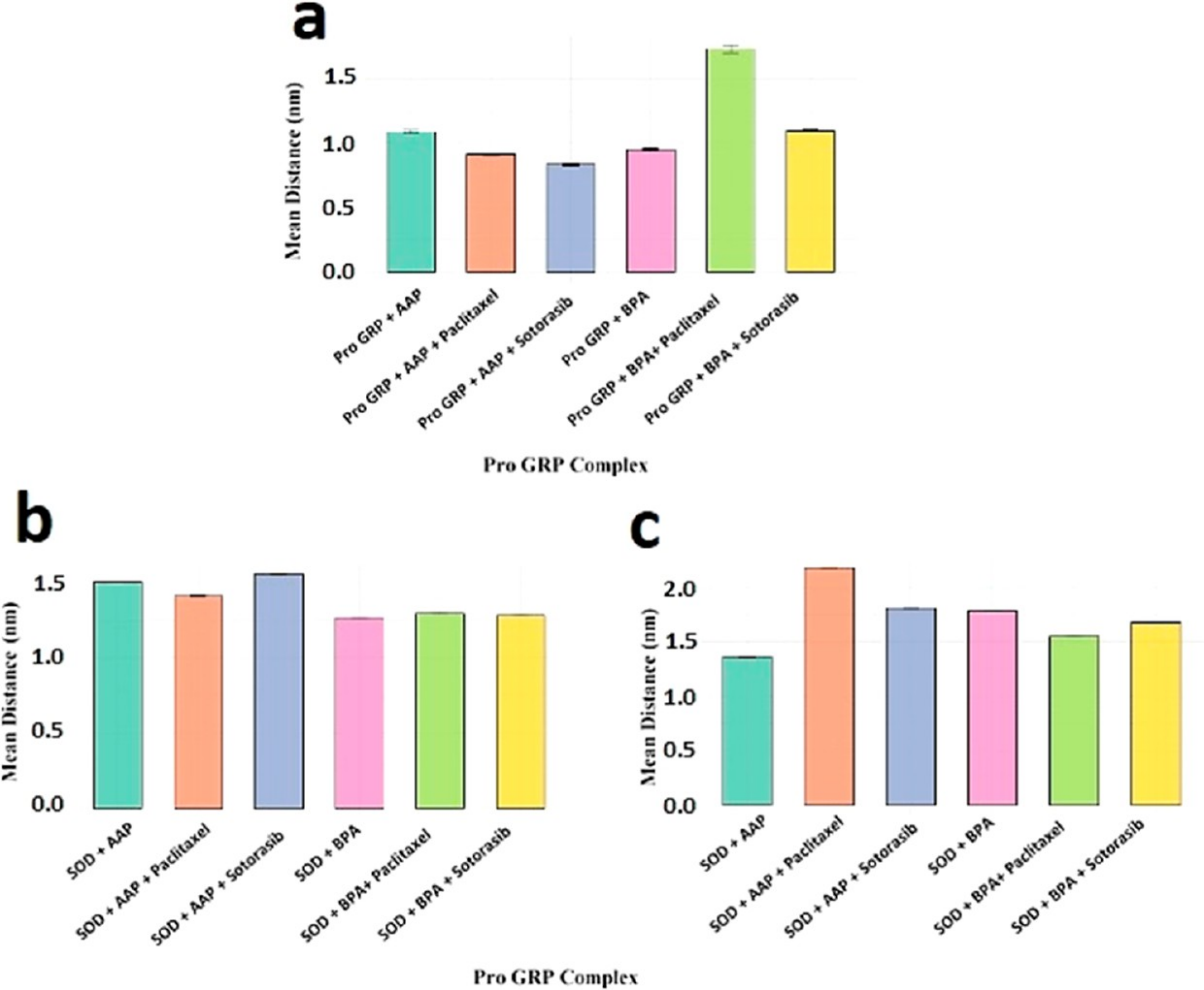
Comparative analysis of the distance between key residues and pollutants in different complexes. (a) Distance variations in Pro-GRP complexes with AAP, BPA, Paclitaxel, and Sotorasib, showing significant shifts in proximity upon drug treatment. (b) Distance measurements in SOD complexes, illustrating distinct trends between chain A and (c) chain B. The effect of drug interactions on pollutant positioning in SOD complexes is highlighted, emphasizing differences between AAP, BPA, and drug-treated conditions. Error bars represent standard deviations, and statistical significance is provided in Table S7.

**Figure 6:**
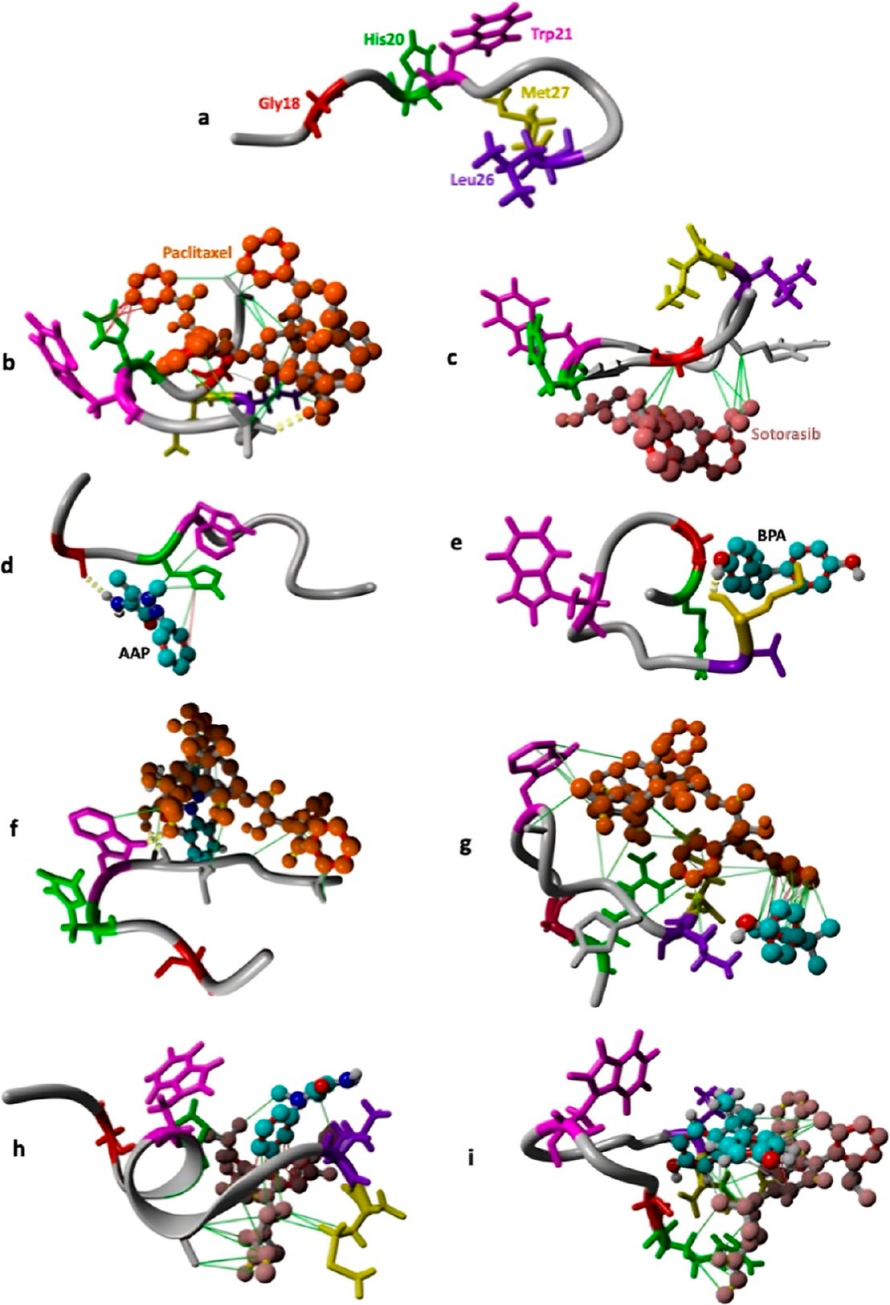
(a) Structural analysis of Pro-GRP complexes with pollutants and anticancer drugs. (b,c) Pro-GRP in complex with the anticancer drugs paclitaxel and sotorasib, respectively. (d,e) Binding interactions of AAP and BPA with Pro-GRP, showing their spatial positioning relative to Trp21. (f,g) Paclitaxel-bound complexes, illustrating its influence on pollutant positioning and protein conformation. (h,i) Sotorasib-bound complexes, showing drug–protein–pollutant interactions and the resulting changes in pollutant binding patterns. These structural observations correspond to the significant shifts in pollutant–Trp21 distances upon drug treatment (Figure 5a), as quantified in Table S7.

To confirm this structural compactness, we analyzed the Rg using the center of mass of the structures (Figures 8 and S4). As shown, this compactness is evident, indicating that both Paclitaxel and Sotorasib induce a more compact conformation of Pro-GRP upon AAP binding after 1000 ns of MDS. This conclusion is more evident when we compare the structure with and without drugs. As we can see (Figures 8a and S4a), both drugs cause Pro-GRP to become more compact.

An interesting result is observed in the case of BPA exposure. While BPA itself can be closer to Trp21 than AAP, both Paclitaxel and Sotorasib cause a significant increase in the distance between the pollutant and Trp21 (Figure 5a). This occurs because both drugs establish multiple interactions with BPA (Figure 6e,g,i), thereby preventing it from approaching the protein. Notably, although Paclitaxel and Sotorasib generally promote a more compact conformation of Pro-GRP in the absence of BPA, their presence leads to reduced compactness when BPA is bound. This provides strong evidence of a distinct mode of structural modulation by these drugs (Figures 8a and S4a).

In the case of SOD (Figure 5b,c), we observe different effects on chain A and chain B. While Paclitaxel decreases the distance between AAP and the four catalytic histidines in chain A, it increases the distance in chain B. In contrast, Sotorasib consistently increases the distance between the pollutant and the catalytic site in both chain A and chain B.

For BPA, we observe a different trend. Unlike in chain B, where both drugs cause a decrease in the distance to the four catalytic histidines, in chain A, there is a slight increase in distance, dependent on both drugs. By observing Figure 7, the reasons for these distance variations become evident. AAP establishes multiple interactions with residues in chain B (Figure 7d). Upon Paclitaxel treatment (Figure 7f), AAP interacts with Paclitaxel, which itself has strong interactions with chain A. Consequently, the pollutant moves away from chain B and gets closer to chain A. Notably, although AAP moves closer to the catalytic residues of chain A, its overall distance from the catalytic site remains greater than when no Paclitaxel was present, and AAP was originally closer to the catalytic site of chain B.

**Figure 7:**
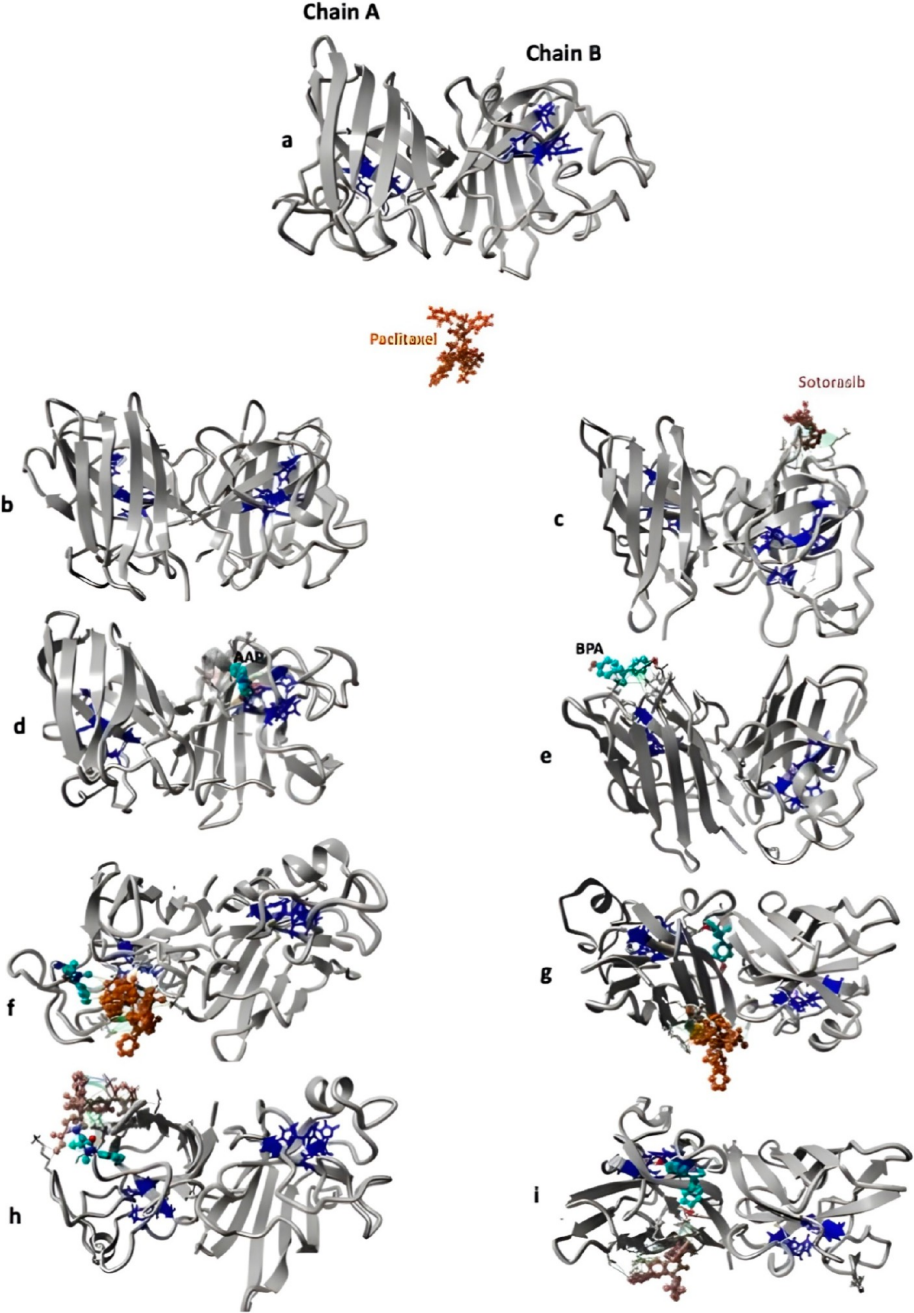
Structural representation of SOD complexes highlighting the interactions between pollutants, anticancer drugs, and the catalytic residues in chains A and B. (a) Native SOD structure with the four catalytic histidines (dark blue) shown in both chains. (b,c) SOD in complex with anticancer drugs paclitaxel and sotorasib, respectively. (d) AAP binding, showing strong interactions with chain B residues. (e) BPA binding, forming extensive interactions with chain A. (f) Paclitaxel-bound complexes illustrating how AAP shifts toward chain A while moving away from chain B due to drug interactions. (g) Paclitaxel-bound BPA complex, showing BPA moving to a more solvent-inaccessible location within SOD. (h,i) Sotorasib-bound complexes, demonstrating that the drug holds AAP away from the catalytic site and shifts BPA toward chain A. These structural insights explain the distance variations shown in Figure 5b,c, where paclitaxel and sotorasib differentially influence pollutant positioning within SOD. The line colors reflect interaction strength and are interpolated between two extremes according to the YASARA scheme: hydrophobic (gray to green), π–π (gray to red), cation−π (gray to blue), and ionic (gray to magenta). This mapping provides a visual representation of interaction strengths and types within the SOD complexes.

Interestingly, in the absence of pollutant exposure, Paclitaxel shows no particular affinity for either chain. This suggests that the presence of pollutants facilitates conformational changes in SOD, enabling Paclitaxel to establish spatial interactions with the protein.

Sotorasib interacts with AAP, preventing it from approaching the catalytic residues and keeping the pollutant farther from both active sites (Figures 5b,c and 7h).

These spatial rearrangements suggest two distinct mechanisms of stabilization. When anticancer drugs displace pollutants from overlapping or nearby binding regions—such as Sotorasib keeping AAP away from the SOD catalytic cavity this pattern is consistent with a competitive binding mechanism. In contrast, when a drug binds at a separate region and still restores compactness and reduces RMSD and total energy, as observed for Paclitaxel, the effect is consistent with allosteric modulation. Together, these trends indicate that the drugs may stabilize the proteins through both direct competition and indirect conformational regulation.

BPA, on the other hand, forms extensive interactions with chain A (Figure 7e), positioning itself close to the catalytic site but on the solvent-accessible surface of this chain. In the presence of both drugs, since these drugs exhibit an affinity for BPA (especially Sotorasib) (Figure S4b), they displace BPA between the two chains (Figure 7g,i), making it more solvent-inaccessible compared to the SOD + BPA complex. This explains why we observed lower RMSF values for both chains in the SOD + BPA + Paclitaxel and SOD + BPA + Sotorasib complexes compared to SOD + BPA (Figure 3c).

One interesting result is observed with Sotorasib: in the absence of pollutants (Figure 7c), it tends to interact with chain B of SOD, but in the presence of both pollutants (Figure 7h,i), its affinity shifts toward chain A, where it interacts with the pollutants. This agrees with the decreased binding affinity observed between the protein and pollutants (Figure 9b), as Sotorasib preferentially binds to both AAP and BPA.

Furthermore, the increase in Rg in anticancer-treated complexes (SOD + BPA + Paclitaxel and SOD + BPA + Sotorasib) (Figure 8) is due to the positioning of BPA within these complexes. BPA is located in the central region of the protein (Figure 7g,i), particularly in the case of Paclitaxel, which reduces compactness compared to the SOD + BPA complex (Figure 8b), where BPA is positioned on the outer surface of chain A.

**Figure 8:**
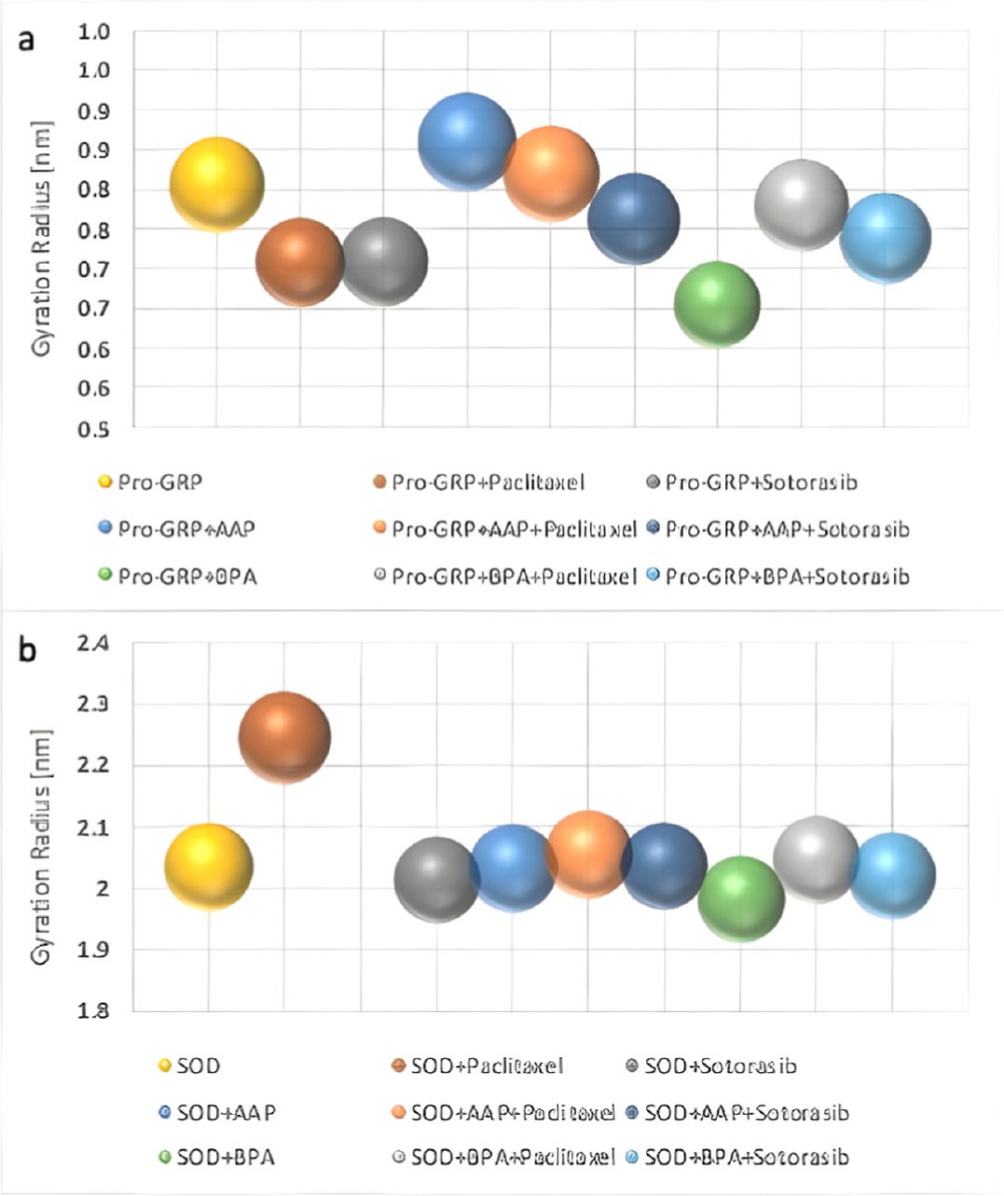
Gyration Radius of Complexes. The radius of gyration (Rg) of each complex after 1000 ns of MDS was calculated using the center of mass to assess structural compactness. The size and height of each sphere represent the Rg values of the respective complexes. BPA significantly increases the compactness of both Pro-GRP and SOD complexes, as reflected in the reduced Rg values. In Pro-GRP complexes, the presence of AAP alone does not significantly alter compactness, but when combined with Paclitaxel or Sotorasib, a notable decrease in Rg is observed, indicating a more compact structure. For SOD complexes, treatment with AAP or BPA results in similar compactness trends, with Paclitaxel and Sotorasib affecting the compactness differently. Overall, these results suggest that pollutants, particularly BPA, play a crucial role in inducing structural compaction, with anticancer drugs further modulating this effect. This analysis highlights the structural impact of pollutants and drugs, which may influence their interactions with the proteins. The Rg of the complexes over 1000 ns is presented in Figure S3.

### Impact of Anticancer Drugs on Pollutant-Protein Binding Affinity: Molecular Docking and MM/PBSA Analysis

To assess the binding strength of pollutants to proteins and the influence of anticancer drugs on their binding affinity, we combined molecular docking and MM/PBSA free-energy calculations (Figures 9, S4 and Table S1). Docking analyses, performed on MD-relaxed protein structures, allowed us to evaluate drug–pollutant–protein complexes in both drug-centric and pollutant-centric contexts. MM/PBSA calculations further quantified the binding free energies and decomposed energetic contributions, providing complementary validation of the docking trends.

**Figure 9:**
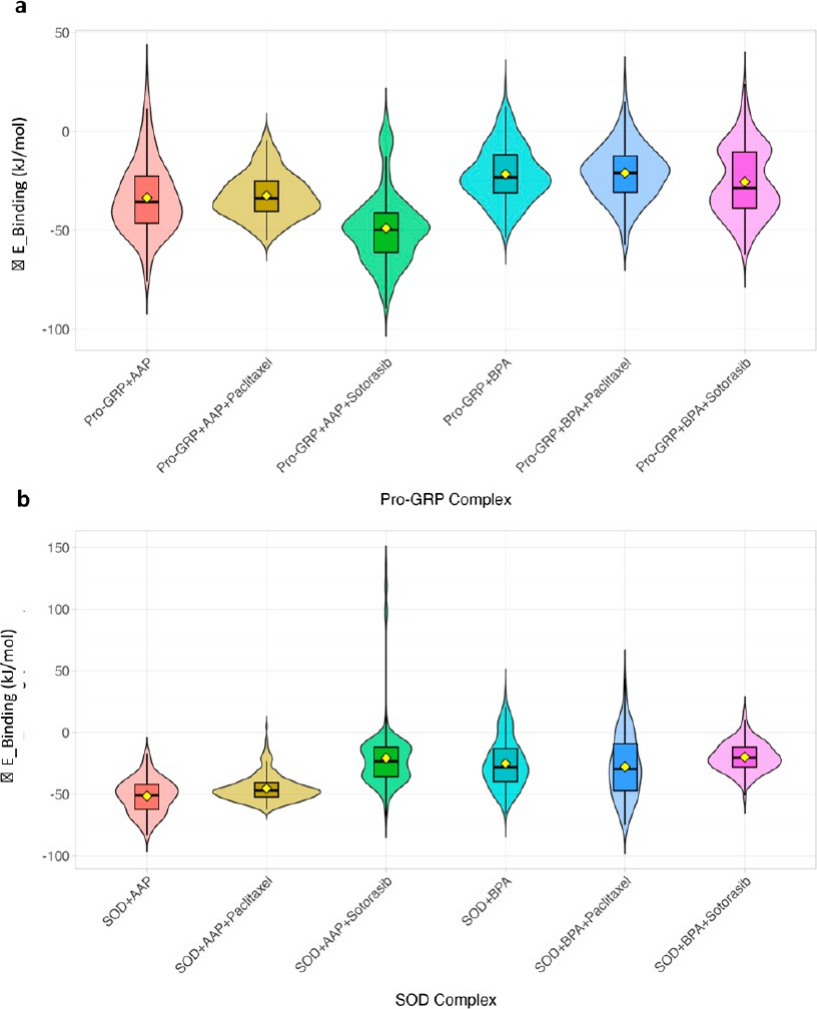
Binding affinity of pollutants to proteins in the presence of anticancer drugs. Violin plots representing the binding free energy (Δ*E*_binding in kcal/mol) of pollutants (AAP and BPA) with Pro-GRP (a) and SOD (b) under different treatment conditions, calculated using MM/PBSA. AAP exhibits significantly stronger binding affinity to both Pro-GRP and SOD compared to BPA. Paclitaxel does not significantly affect the binding affinity of pollutants with Pro-GRP, but in SOD, it significantly reduces AAP binding affinity while having no significant effect on BPA binding. Sotorasib increases the binding affinity of pollutants to Pro-GRP but reduces their binding to SOD. Interestingly, AAP interacts more strongly with Sotorasib than Paclitaxel in SOD, while in Pro-GRP, Paclitaxel exhibits a significantly higher affinity for AAP than Sotorasib, aligning with previous interaction analyses. These results suggest that anticancer drugs differentially modulate pollutant-protein binding affinities, with AAP consistently exhibiting stronger interactions than BPA across conditions.

Molecular docking on MD-relaxed protein structures showed distinct binding patterns for Pro-GRP and SOD. In Pro-GRP, BPA alone displayed slightly stronger binding than AAP (Δ*G* ≈ −4.0 vs −3.6 kcal·mol^–1^). However, in the presence of anticancer drugs, AAP consistently acted as a positive modulator: for example, [GRP + AAP]+Paclitaxel bound more strongly than GRP + Paclitaxel, whereas BPA reduced drug affinity (e.g., [GRP + BPA]+Paclitaxel vs GRP + Paclitaxel). In SOD, AAP exhibited stronger binding than BPA (Δ*G* ≈ −6.4 vs −5.9 kcal·mol^–1^), and also enhanced Paclitaxel binding ([SOD + AAP] + Paclitaxel vs SOD + Paclitaxel), while BPA weakened drug interactions ([SOD + BPA] + Paclitaxel vs SOD + Paclitaxel). Thus, docking suggested that AAP may differentially modulate drug binding, with clearer advantages in SOD than in Pro-GRP.

To validate and refine these predictions, MM/PBSA free-energy calculations were performed. For both Pro-GRP and SOD, AAP exhibits significantly higher binding affinity to the proteins compared to BPA (Figure 9), consistent with the trend in SOD and clarifying the ambiguous docking results in Pro-GRP.

In the pollutant-centric comparison, the effect of adding drugs to pollutant–protein complexes was evaluated. Although docking results indicated that in **Pro-GRP**, drugs slightly stabilized pollutant binding in all cases: [GRP + Paclitaxel] + AAP and [GRP + Sotorasib] + AAP showed improved stability relative to GRP + AAP (ΔΔ*G* ≈ −0.98 and −0.40), and similar enhancements were observed for BPA (ΔΔ*G* ≈ −0.35 for both), MM/PBSA results indicated that Paclitaxel does not significantly (Table S8) affect the binding affinity of either pollutant with Pro-GRP.

By contrast, docking results for **SOD** showed that adding drugs weakened AAP binding ([SOD + Paclitaxel] + AAP and [SOD + Sotorasib]+AAP, ΔΔ*G* ≈ +0.37 and +0.85), while only minimal stabilization was observed for BPA ([SOD + Paclitaxel] + BPA and [SOD + Sotorasib] + BPA, ΔΔ*G* ≈ −0.20 and −0.21. However, MM/PBSA showed that Paclitaxel has no significant effect on BPA binding but markedly decreases the affinity of AAP for this protein. Moreover, MM/PBSA further indicated that Sotorasib decreases the binding affinity of both pollutants in SOD.

Both docking and MM/PBSA analyses demonstrated that the affinity of both pollutants for Pro-GRP increased in the presence of Sotorasib; however, AAP consistently exhibited a much higher affinity than BPA.

As shown by the MM/PBSA results, both Sotorasib and Paclitaxel exhibited stronger binding to AAP within SOD. This suggests that the increased affinity of these drugs for the [SOD + AAP] complexes (Table S1) is primarily driven by drug–AAP interactions, which in turn may account for the weakened binding of AAP observed in the [SOD + Paclitaxel] and [SOD + Sotorasib] complexes.

So However, in Pro-GRP, Paclitaxel shows a significantly stronger affinity for AAP than Sotorasib (Figure S4 and Table S9).

Taken together, these results suggest that AAP generally shows stronger interactions than BPA, particularly in SOD, while the modulatory effects of Paclitaxel and Sotorasib appear to be protein-specific and context-dependent. Although docking and MM/PBSA analyses reveal consistent overall trends, some differences between the two approaches highlight the need for cautious interpretation and further validation.

Multipanel comparative bar charts represent five terms van der Waals, electrostatic, polar solvation, nonpolar solvation (SASA-based), and binding energy to further assess the influence of anticancer drugs on pollutant-protein interactions (Figure 10). More data align with the Δ*E* analysis.

**Figure 10:**
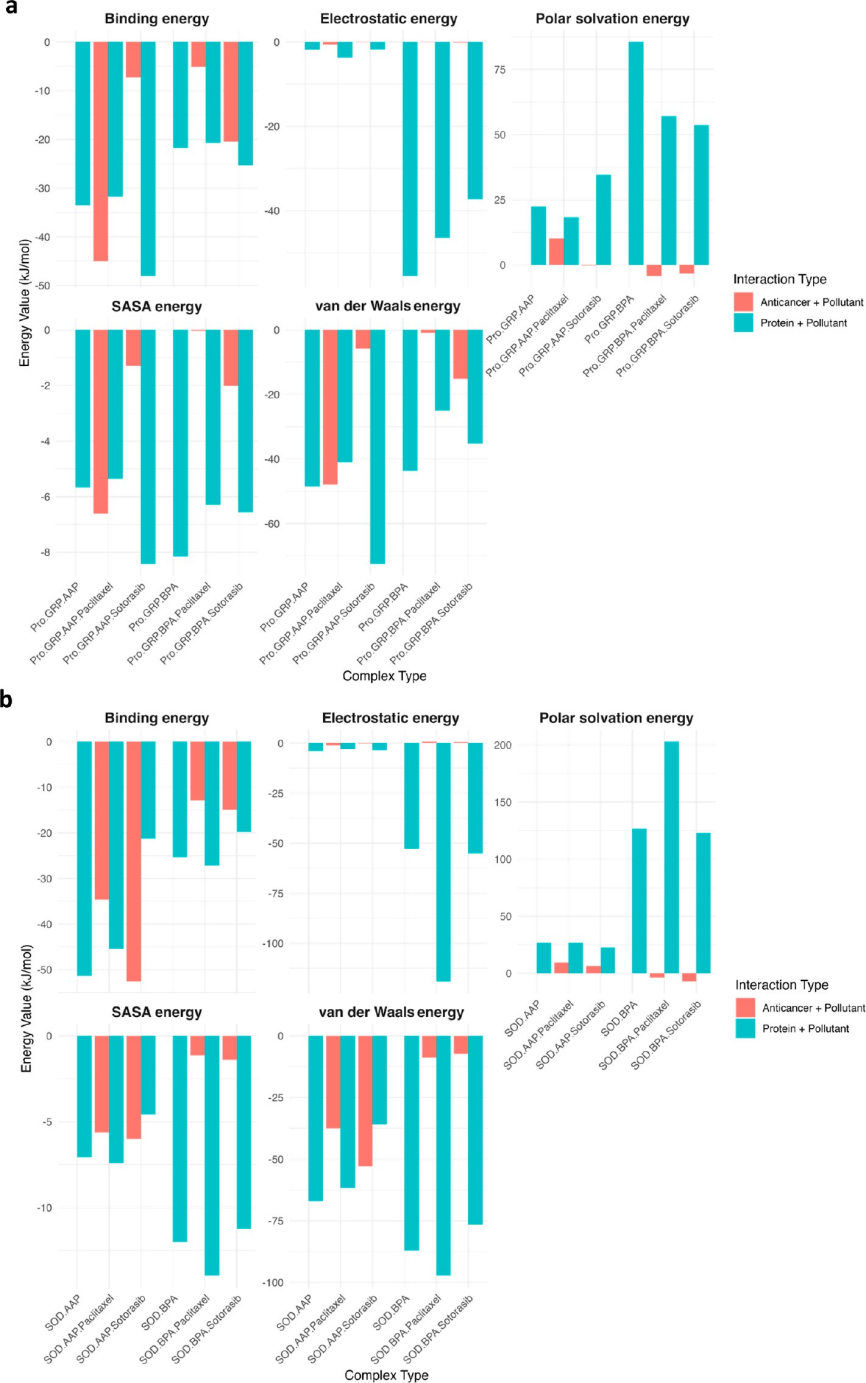
Comparison of MM/PBSA energy components for pollutant-protein and pollutant-anticancer interactions. (a) Energy decomposition analysis for pro-GRP complexes with and without anticancer drugs (Paclitaxel, Sotorasib). (b) Energy decomposition analysis for SOD complexes in similar conditions. Five key energy components are shown: van der Waals energy, electrostatic energy, polar solvation energy, SASA energy, and binding energy. The blue bars represent protein-pollutant interactions, while the red bars correspond to pollutant-anticancer drug interactions. Higher polar solvation energy indicates solvation penalties, while shifts in van der Waals and electrostatic contributions highlight the impact of anticancer drugs on pollutant binding affinity.

One interesting finding is that for Pro-GRP, Pro-GRP + AAP + Sotorasib, compared to the reference (Pro-GRP + AAP), shows a higher affinity for the pollutant. A similar trend is observed for SOD + BPA + Paclitaxel compared to its reference (SOD + BPA). In the case of lung cancer, this could be promising because, as found, Pro-GRP and mnSOD (or SOD1, which is the same SOD we studied) are expressed at higher levels in lung cancer.^8,44^ Additionally, therapy monitoring in small cell lung cancer (SCLC) patients suggests that a decrease in Pro-GRP levels is an optimal marker for successful therapy,^45^ and inhibition of SOD1 by small molecules induces cell death in various nonsmall cell lung cancer (NSCLC) cells, including those harboring KRAS mutations.^46^ It may be a good approach to use both AAP and Sotorasib to reduce elevated Pro-GRP levels in SCLC and BPA and Paclitaxel to lower SOD levels in NSCLC. However, as clearly stated, further studies are needed to assess this, especially regarding potential side effects and toxicity to normal cells compared to cancer cells.

The MM/PBSA energy-decomposition results further support these mechanistic interpretations, showing that complexes characterized by competitive displacement are dominated by van der Waals and electrostatic contributions, whereas allosteric stabilization is associated with reduced polar-solvation penalties and more favorable nonpolar (SASA) terms.

To better understand the binding affinity of pollutants to key protein residues, we analyzed the per-residue total binding free energies using MM/PBSA calculations. As shown in Figure 11, AAP exhibited a significant interaction with Trp21, suggesting a strong binding affinity at this site. However, Paclitaxel significantly reduced this affinity, indicating that its presence disrupts AAP binding, likely due to steric hindrance or competition for the binding pocket. The interactions between AAP and Paclitaxel shown in Figure 6 support this observation. Interestingly, Sotorasib did not exhibit the same effect, suggesting that its interaction with the protein does not interfere with AAP’s binding in the same manner. This may be due to the increased compactness (Figure 8) and conformational changes caused by the numerous interactions that Sotorasib forms with the protein, ultimately making AAP more accessible.

**Figure 11:**
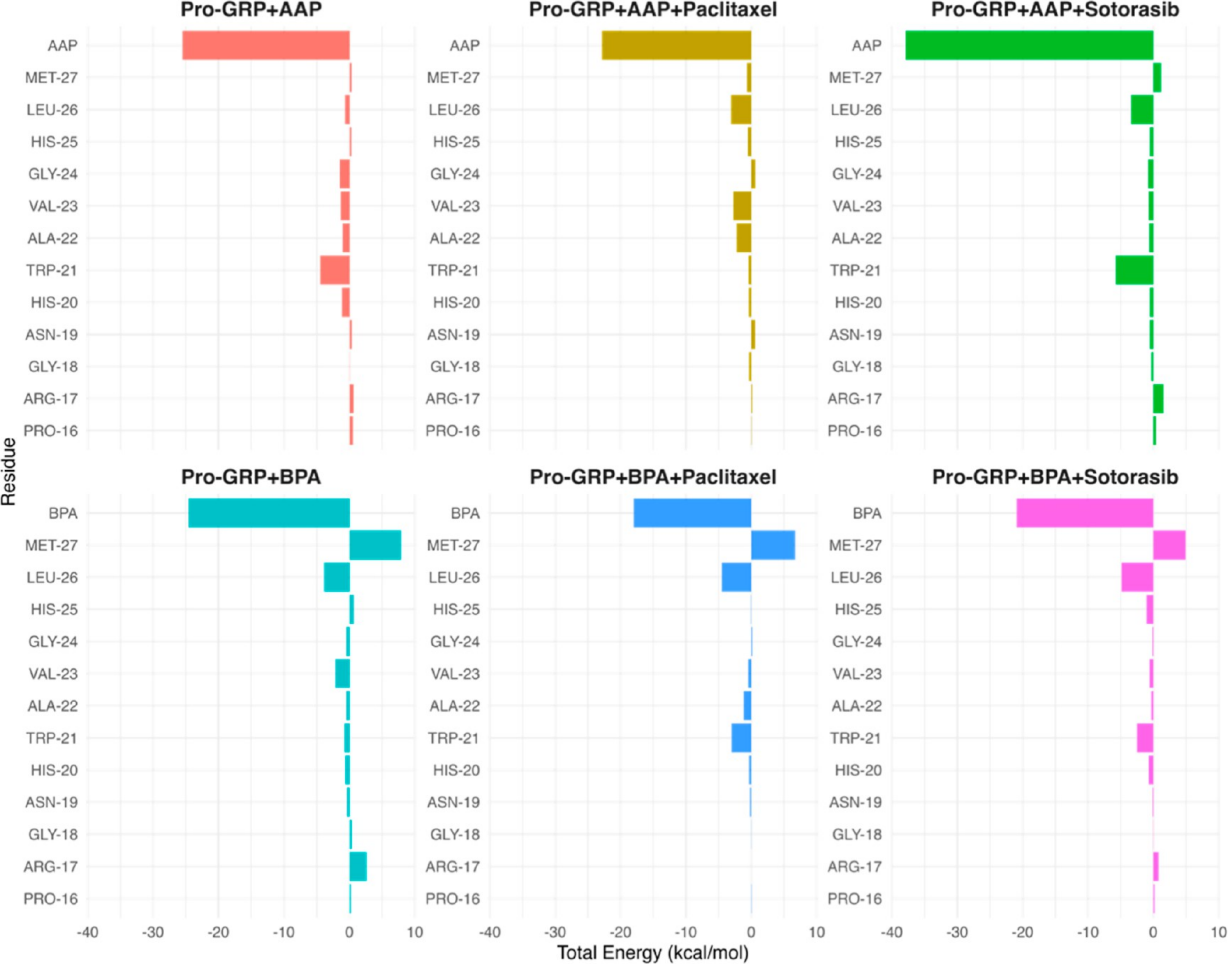
Per-residue total binding free energy analysis of pollutant-protein interactions using MM/PBSA calculations. AAP demonstrated a strong affinity for Trp21, which was significantly reduced in the presence of Paclitaxel, likely due to steric hindrance or competition for the binding pocket. Sotorasib, however, did not disrupt AAP’s binding, suggesting a different mode of interaction. In contrast, BPA showed no significant affinity for Trp21 alone, but its binding was enhanced in the presence of Paclitaxel and Sotorasib, indicating potential allosteric effects or indirect stabilization. These results highlight the differential impact of anticancer drugs on pollutant binding and suggest that drug-induced structural or energetic modifications can influence environmental toxin interactions with proteins.

For BPA, there was no notable affinity for Trp21, implying that this pollutant does not strongly interact with this key residue. However, an interesting observation is that both Paclitaxel and Sotorasib increased BPA’s affinity for Trp21. This may indicate a possible synergistic effect or allosteric modulation induced by the anticancer drugs. Such an effect could arise from indirect stabilization due to the interactions of the drugs with the protein (Figure 6), increased compactness (Figure 8), or an altered electrostatic environment that facilitates BPA binding.

These findings highlight the differential impact of anticancer drugs on pollutant-protein interactions, suggesting that drug-induced structural or energetic modifications can influence environmental toxin binding.

For SOD, we observe fluctuations (different total energy) in the binding affinity between residues and pollutants in both chains. Evaluation of this parameter for catalytic residues (His46, His48, His63, and His120) also shows different results. Upon treatment with Paclitaxel, a decrease in affinity (higher total energy) is observed for His46, His48, and His63 in chain A, as well as His46, His63, and His120 in chain B (Figure 12).

**Figure 12:**
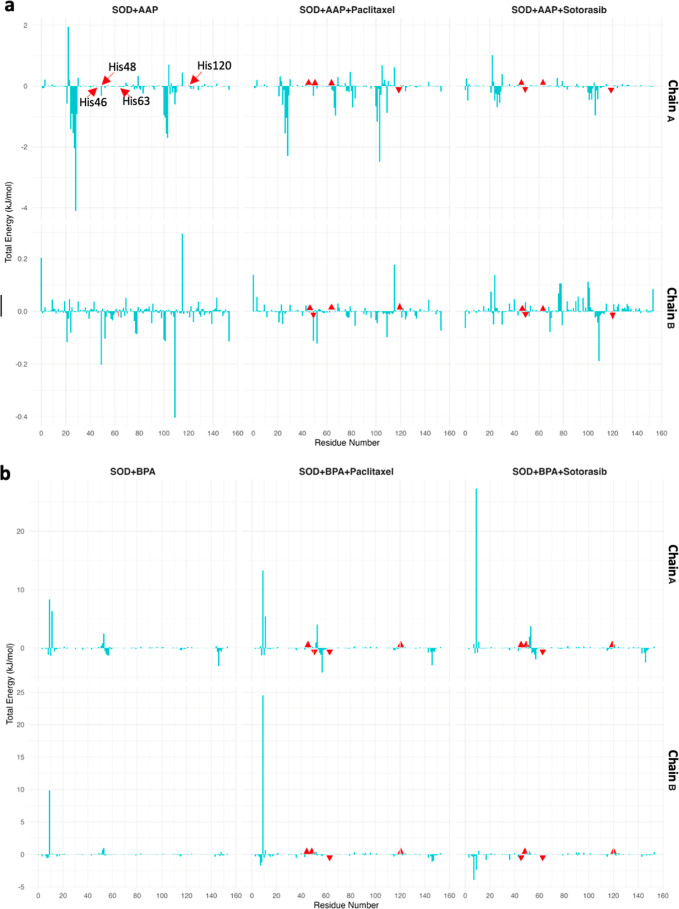
Per-residue total binding free energy contributions for SOD in the presence of pollutants (AAP and BPA) and anticancer drugs (Paclitaxel and Sotorasib). The top panel (a) illustrates the energy contributions of individual residues in the SOD + AAP complexes, while the bottom panel (b) represents the SOD + BPA complexes. Red-highlighted bars (identified by red arrows in the first graph) indicate catalytic residues (His46, His48, His63, and His120). The upward and downward red markers indicate the difference in total energy contributions of residues and pollutants compared to the reference structure.

By comparing these observations with what we see in Figure 7d, the reason becomes clearer. In the SOD-AAP complex, AAP is located in chain B, close to the catalytic site (Figure 5b,c). However, upon treatment with Paclitaxel, the drug forms numerous interactions with AAP and exhibits a high affinity for this pollutant (Figure S4). Additionally, since Paclitaxel interacts extensively with chain A of SOD, AAP is displaced further from chain B, but it is not close to the catalytic site of chain A either.

Sotorasib shows similar behavior in the total energy of AAP and catalytic residues for both chains of SOD, decreasing the affinity of His46 and His63, while increasing the affinity of His48 and His120.

For BPA, Paclitaxel decreases the pollutant’s affinity for His46 and His120 of chain A while increasing its affinity for His48 and His63 in the same chain. In chain B, except for His63, Paclitaxel reduces the binding affinity of BPA to the catalytic residues. In the SOD-BPA complex (Figure 7e), BPA is closer to the catalytic site of chain A, but upon exposure to Paclitaxel, it moves farther away (Figure 5b,c). However, as Paclitaxel is positioned approximately in the middle of the protein structure between the two chains, it facilitates BPA’s localization in the solvent-inaccessible region between the chains, distancing it from both active sites (Figure 7g).

The effect of Sotorasib, which forms strong interactions with chain A (Figure 7i), differs from that of Paclitaxel. This anticancer drug has a stronger affinity for BPA than Paclitaxel (Figure S4b), which results in a lower affinity for BPA at all catalytic residues except His63. In chain B, Sotorasib causes BPA to move closer to His46 and His63 compared to its position in the SOD + BPA complex (Figures 5b and 7e,i). As a result, BPA shows a higher affinity for these residues when compared to the SOD + BPA complex.

Although only two pollutants and two drugs were modeled, they can be regarded as representative prototypes of broader chemical and pharmacological categories, suggesting that comparable destabilization or stabilization trends might be observed for structurally related compounds.

## Conclusion

This study provides insights into the molecular effects of environmental pollutants on lung cancer–associated proteins and their interactions with anticancer agents. Our findings reveal that pollutants such as AAP and BPA induce structural instability in Pro-GRP and SOD, whereas anticancer drugs, particularly paclitaxel, partially restore structural stability. RMSD, RMSF, energy profiles, and MM/PBSA analyses consistently showed that paclitaxel exerted a stronger stabilizing effect than sotorasib, especially against AAP-induced perturbations.

Our findings show that Pro-GRP + AAP + Sotorasib and SOD + BPA + Paclitaxel complexes exhibited stronger binding affinities than the corresponding protein–pollutant complexes without drug. Since Pro-GRP and SOD1 are elevated in lung cancer, particularly in SCLC and NSCLC, our results suggest that pollutants (AAP and BPA) can destabilize these proteins, whereas anticancer agents such as Sotorasib and Paclitaxel may partially counteract this effect. These observations are exploratory, require experimental validation, and should not be interpreted as implying any therapeutic role for environmental pollutants.

Overall, our analyses demonstrate that environmental pollutants can destabilize key lung cancer biomarkers, while anticancer drugs may counteract these effects. Notably, Paclitaxel consistently showed a greater capacity than Sotorasib to stabilize pollutant-exposed Pro-GRP and SOD, underscoring its stronger protective role in this computational framework. These results highlight the importance of considering both environmental exposures and drug-specific stabilization mechanisms when evaluating protein structure and function in lung cancer biology.

The reliance on computational methods without in vitro or in vivo validation limits the translational applicability of the findings. Future studies integrating biochemical assays and cellular models will be essential to confirm these predictions and establish their biological and clinical relevance.

## Limitations

Our research focuses exclusively on acute, single-pollutant binding events observed in MD simulations and does not encompass dose-dependent or chronic exposure scenarios, which are essential in the field of environmental toxicology. The effects of pollutants may escalate over time due to repeated interactions or bioaccumulation, which could exacerbate protein destabilization in vivo.

Furthermore, the dependence on computational docking and MD simulations without corresponding in vitro or in vivo validation restricts the translational applicability of the results, as these simulations yield only structural hypotheses. Future research should incorporate kinetic models for chronic exposure, along with biochemical assays and cellular systems, to validate biological and clinical relevance.

We examined only two pollutants (AAP, BPA) and two anticancer drugs (paclitaxel, sotorasib). A broader set of pollutants and drugs should be tested to evaluate whether the observed trends are generalizable.

Concentration effects and chronic exposure scenarios were not modeled; these remain important directions for future research.

## Supplementary Material



## Notes

Additional figures, tables, and experimental details are provided here to complement the results discussed in the main text (PDF)

## Abbreviations

AAP: aminoantipyrine
BPA: bisphenol A
CAT: catalase
EGFR: epidermal growth factor receptor
ERK1/2: extracellular signal-regulated kinase 1/2
GPER: G protein-coupled estrogen receptor
GPx: glutathione reductase
GRP: gastrin-releasing peptide
KRAS: Kirsten rat sarcoma viral oncogene homologue
MDS: molecular dynamics simulations
MM/PBSA: molecular mechanics/Poisson/Boltzmann surface area
NSCLC: non-small cell lung cancer
PACs: polycyclic aromatic compounds
PDB: Protein Data Bank
Pro-GRP: pro-gastrin-releasing peptide
Rg: radius of gyration
RMSD: root mean square deviation
RMSF: root mean square fluctuation
ROS: reactive oxygen species
SCLC: small-cell lung cancer
SOD: superoxide dismutase.
