# Supplementary material for: Environmental Pollutants and Protein Destabilization in Lung Cancer: Anticancer Drug Strategies for Structural Stability
Source: ACS Omega. 2026 Jan 1;11(2):2513–32. doi: 10.1021/acsomega.5c06228 (PMC12824962; doi:10.1021/acsomega.5c06228)
Supplement: Supplementary file 1 [file ao5c06228_si_001.pdf]

Environmental Pollutants and Protein Destabilization in Lung Cancer: Anticancer Drug Strategies for Structural Stability

Reza Rasoolzadeh[1#*], Homa Faraji[2,3#], Leonardo Baptista[4*], Fahimeh Sadat Vajedi[5], Vahid Nikoofard[6], Luciano T. Costa[7], José Walkimar de M. Carneiro[1]

1. Department of Inorganic Chemistry, Institute of Chemistry, Fluminense Federal University, Niterói, Rio de Janeiro 24020-141, Brazil

2. Biosensor Research Center, Endocrinology and Metabolism Molecular-Cellular Sciences Institute, Tehran University of Medical Sciences, Chamran Highway, Jalal-Al-Ahmad Street, Tehran, 1411713137, Iran

3. SpentaGen Computational Biology and AI Group, Tehran, Iran

4. Department of Chemistry and Environmental, Faculty of Technology, Rio de Janeiro State University, Resende, Rio de Janeiro 27537-000, Brazil

5. Department of Chemistry, Institute of Chemistry, Rio de Janeiro State University, Rio de Janeiro, Rio de Janeiro 20550-900, Brazil

6. Department of Mathematics, Physics and Computation, Faculty of Technology, Rio de Janeiro State University, Resende, Rio de Janeiro 27537-000, Brazil

7. Institute of Chemistry, Fluminense Federal University, Campus Valonguinho, Centro, Niterói, Rio de Janeiro CEP 24020-141, Brazil

# All authors have contributed equally.

* Corresponding authors:

Reza Rasoolzadeh: Reza_Rasoolzadeh@id.uff.br
Leonardo Baptista: leobap@gmail.com

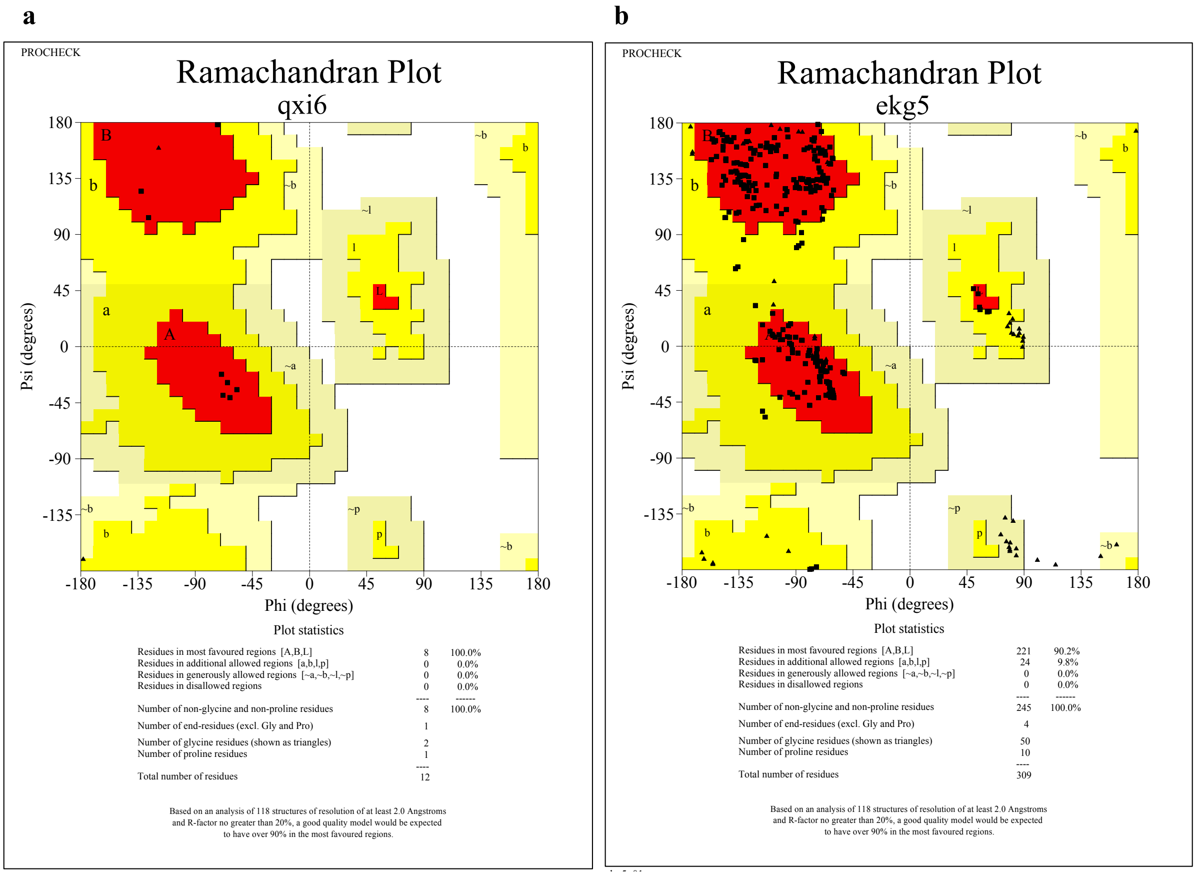

Sup. Figure 1. Ramachandran Plot Analysis of Pro-GRP and SOD Structures. Ramachandran plots of (a) Pro-GRP and (b) SOD, illustrating the distribution of backbone dihedral angles (phi, psi) and structural validation of the final optimized structures. The red regions represent the most favored conformations, yellow regions indicate allowed conformations, and light yellow regions represent generously allowed conformations. In (a) Pro-GRP, all residues fall within the most favored regions (100%), indicating a highly stable and well-folded structure. In (b) SOD, 90.2% of residues are in the most favored regions, with 9.8% in additional allowed regions, demonstrating a well-refined and reliable structure. No residues are present in disallowed regions in either model, confirming the overall high stereochemical quality of the protein structures.

Sup. table 1. Molecular docking outcomes for GRP and SOD complexes built on MD-relaxed (MDS) protein structures.

Structure	Best Binding Energy (kcal/mol)	Dissociation Constant (pM)	Contact Surface (A^2)	Interacting Residues
GRP+AAP	- 3.6360	2161606656	151.46	GLY 18 ASN 19 TRP 21 ALA 22 VAL 23 LEU 26
GRP+ BPA	- 4.0210	1128672384	168.50	ASN 19 HIS 20 TRP 21 ALA 22 VAL 23 LEU 26 NH2 28
GRP+Paclitaxel	- 5.7630	59657492	299.22	ARG 17 GLY 18 ASN 19 HIS 20 TRP 21 ALA 22 VAL 23 LEU 26 MET 27 NH2 28
GRP+Sotorasib	- 5.8330	53009572	255.95	ARG 17 GLY 18 ASN 19 HIS 20 TRP 21 ALA 22 VAL 23 LEU 26 MET 27
[GRP+AAP]+Paclitaxel	- 6.5020	17138420	309.75	A PRO 1 A ARG 2 A ASN 4 A HIS 5 A TRP 6 A ALA 7 AAP
[GRP+AAP]+Sotorasib	-6.0110	39253516	231.40	A PRO 1 A ARG 2 A ASN 4 A HIS 5 A TRP 6 A ALA 7 A VAL 8 AAP

[GRP+BPA]+Paclitaxel	-5.2500	141807424	219.51	A PRO 16 A ARG 17 A TRP 21 A ALA 22 A GLY 24 A HIS 25
[GRP+BPA]+Sotorasib	- 5.6090	77365704	200.91	A PRO 16 A GLY 18 A ASN 19 A HIS 20 A TRP 21 A MET 27 B BPA
[GRP+Paclitaxel]+AAP	-4.6140	414851968	155.81	A TRP 21 B Paclitaxel
[GRP+Sotorasib]+AAP	-4.0360	1100456192	141.38	A HIS 20 B S3R 28
[GRP+Paclitaxel]+BPA	-4.3720	624139264	130.81	A ASN 19 A HIS 20 A TRP 21 A ALA 22
[GRP+Sotorasib]+BPA	-4.3720	24139264	130.05	A ASN 19 A HIS 20 A TRP 21 A ALA 22
SOD+AAP	- 6.4230	19582970	266.94	A ALA -1 A MET 0 A ALA 1 A LEU 106 A SER 107 A GLY 108 A CYS 111 A ILE 113 A ILE 151 B ALA 1 B ASP 109 B CYS 111 B ILE 113 B ARG 115 B ILE 151
SOD+BPA	- 5.8900	48147392	252.60	A PRO 74 A ARG 79 A ASP 83 A LEU 84 A GLY 85 A ASN 86 A VAL 87 A SER 98 A ILE 99 A GLU 100 A ASP 101 A LEU 126
SOD+Paclitaxel	- 8.5520	538642.8125	538.57	A VAL 7 A LEU 8 A LYS 9 A GLY 10 A ASP 11 A GLY 51 A ASP 52 A ASN 53 A ALA 55 A GLY 56 A CYS 146 A GLY 147 A VAL 148 B CYS 6 B VAL 7 B LEU 8 B LYS 9 B GLN 15 B GLY 16 B ILE 17 B GLY 147 B VAL 148
SOD+Sotorasib	- 8.6250	476201.9688	438.37	A VAL 7 A LYS 9 A GLY 51 A ASN 53 A CYS 146 A GLY 147 A VAL 148 B CYS 6 B VAL 7 B LYS 9 B GLY 10 B ASP 11 B ASN 53 B THR 54 B ALA 55 B GLY 56 B CYS 57 B CYS 146 B GLY 147 B VAL 148
[SOD+AAP]+Paclitaxel	- 8.8630	318665.3750	517.36	A HIS 48 A GLU 49 A PHE 50 A ALA 55 A GLY 56 A SER 59 A ALA 60 A GLY 61 A PRO 62 A LEU 67 A SER 68 A ARG 69 A LYS 70 A HIS 71 A THR 116 A VAL 118 A LYS 136 A ASN 139 B MET 0 B ALA 152 B GLN 153
[SOD+AAP]+Sotorasib	-8.7970	356216.0625	410.06	A HIS 48 A GLU 49 A PHE 50 A ALA 55 A GLY 56 A SER 59 A ALA 60 A GLY 61 A PRO 62 A SER 68 A HIS 71 A ASN 139 B MET 0 B ALA 152 B GLN 153
[SOD+BPA]+Paclitaxel	-7.3780	3907098	374.15	A LYS 75 A ARG 79 A ASP 83 A LEU 84 A GLY 85 A ASN 86 A VAL 87 A THR 88 A ALA 95 A ASP 96 A VAL 97 A SER 98 A ILE 99 A GLU 100 A ASP 101 A LEU 126 A GLY 127
[SOD+BPA]+Sotorasib	- 8.4060	689160.3750	340.10	A GLN 153 B GLU 49 B PHE 50 B THR 58 B SER 59 B ALA 60 B GLY 61 B PRO 62 B GLY 72 B GLY 73 B PRO 74 B GLY 130 B ASN 131 B GLU 132 B LYS 136
[SOD+Paclitaxel]+AAP	-6.0580	36259944	250.10	A CYS 6 A VAL 7 A LYS 9 A ASP 52 A ASN 53 A ALA 55 A GLY 56 A CYS 57 A THR 116 A CYS 146 A GLY 147 A VAL 148 B GLY 51 B ASP 52 B ASN 53 B THR 54 B GLY 147 B VAL 148
[SOD+Sotorasib]+AAP	-5.5700	82629640	239.74	A GLU 49 A PHE 64 A ASN 65 A PRO 66 A SER 68 A GLY 108 A ASP 109 A CYS 111 A ARG 115 B MET 0 B ALA 1 B GLY 108 B ASP 109 B CYS 111 B ILE 113 B ILE 151
[SOD+Paclitaxel]+BPA	-6.0940	34122336	264.98	A VAL 7 A LEU 8 A LYS 9 A ASN 53 A THR 54 A CYS 146 A GLY 147 A VAL 148 B VAL 7 B LYS 9 B ASN 53 B THR 54 B GLY 147 B VAL 148
[SOD+Sotorasib]+BPA	-6.1010	33721564	224.69	A VAL 5 A CYS 6 A VAL 7 A ILE 17 A ILE 18 A ASN 19 A ALA 152 B PHE 50 B GLY 51 B ASP 52 B THR 54 B ALA 55

Entries in brackets (e.g., **[Protein+Ligand1]+Ligand2**) denote **ternary complexes** where the second ligand was docked onto the **MD-relaxed binary complex**. Reported values include the best binding free energy **ΔG** (kcal·mol^{-1}; more negative indicates stronger binding), the dissociation constant **K_d** (pM; recalculated from ΔG using ΔG = RT ln K_d at 298 K), the **contact surface area** (Å^2), and the **contacting residues** at the protein–ligand interface. See Methods for structure preparation, MD relaxation and docking/scoring details.

Sup. Table 2 Details of Molecular Dynamics Simulations

complexes

simulation systems	no. of Anticancer	no. of Pollutant	no. of protein	Type and no. of added ions	temperature (K)	box dimensions (nm)	duration (ns)
Pro-GRP	0	0	1	Cl^-: 1	310	5.21×521×5.21	1000×3
Pro-GRP+Paclitaxel	1	0	1	Cl^-: 1	310	5.21×521×5.21	1000×3
Pro-GRP+Sotorasib	1	0	1	Cl^-: 1	310	5.21×521×5.21	1000×3
Pro-GRP+AAP	0	1	1	Cl^-: 1	310	5.21×521×5.21	1000×3
Pro-GRP+AAP+Paclitaxel	1	1	1	Cl^-: 1	310	5.21×521×5.21	1000×3
Pro-GRP+AAP+Sotorasib	1	1	1	Cl^-: 1	310	5.21×521×5.21	1000×3
Pro-GRP+BPA	0	1	1	Cl^-: 1	310	5.21×521×5.21	1000×3
Pro-GRP+BPA+Paclitaxel	1	1	1	Cl^-: 1	310	5.21×521×5.21	1000×3
Pro-GRP+BPA+Sotorasib	1	1	1	Cl^-: 1	310	5.21×521×5.21	1000×3
SOD	0	0	1	Na^+: 8	310	9.05×9.05×9.05	1000×3
SOD+Paclitaxel	1	0	1	Na^+: 10	310	9.05×9.05×9.05	1000×3
SOD+Sotorasib	1	0	1	Na^+: 10	310	9.05×9.05×9.05	1000×3
SOD+AAP	0	1	1	Na^+: 10	310	9.05×9.05×9.05	1000×3
SOD+AAP+ Paclitaxel	1	1	1	Na^+: 10	310	9.05×9.05×9.05	1000×3
SOD+AAP+Sotorasib	1	1	1	Na^+: 10	310	9.05×9.05×9.05	1000×3
SOD+BPA	0	1	1	Na^+: 10	310	9.05×9.05×9.05	1000×3
SOD+BPA+Paclitaxel	1	1	1	Na^+: 10	310	9.05×9.05×9.05	1000×3
SOD+BPA+Sotorasib	1	1	1	Na^+: 10	310	9.05×9.05×9.05	1000×3

Sup. Table 3 Pairwise Statistical Comparisons of RMSD Values Among Complexes with Pollutants and Drugs (Games-Howell Test Results)

Pro-GRP complexes					
Group1	Group2	Mean_Diff (Δ)	p_value	p_adj	Significance
Pro-GRP	Pro-GRP+AAP	0.0837	0	0	****
Pro-GRP	Pro-GRP+AAP+Paclitaxel	-0.1918	0	0	****
Pro-GRP	Pro-GRP+AAP+Sotorasib	-0.0794	0	0	****
Pro-GRP	Pro-GRP+BPA	0.0412	0	0	****
Pro-GRP	Pro-GRP+BPA+Paclitaxel	-0.105	0	0	****
Pro-GRP	Pro-GRP+BPA+Sotorasib	-0.0103	0	0	****
Pro-GRP	Pro-GRP+Paclitaxel	0.0328	0	0	****
Pro-GRP	Pro-GRP+Sotorasib	0.0626	0	0	****
Pro-GRP+AAP	Pro-GRP+AAP+Paclitaxel	-0.2755	0	0	****
Pro-GRP+AAP	Pro-GRP+AAP+Sotorasib	-0.163	0	0	****
Pro-GRP+AAP	Pro-GRP+BPA	-0.0425	0	0	****
Pro-GRP+AAP	Pro-GRP+BPA+Paclitaxel	-0.1886	0	0	****
Pro-GRP+AAP	Pro-GRP+BPA+Sotorasib	-0.094	0	0	****
Pro-GRP+AAP	Pro-GRP+Paclitaxel	-0.0508	0	0	****
Pro-GRP+AAP	Pro-GRP+Sotorasib	-0.0211	0	0	****
Pro-GRP+AAP+Paclitaxel	Pro-GRP+AAP+Sotorasib	0.1124	0	0	****
Pro-GRP+AAP+Paclitaxel	Pro-GRP+BPA	0.233	0	0	****

Pro-GRP+AAP+Paclitaxel	Pro-GRP+BPA+Paclitaxel	0.0868	0	0	****
Pro-GRP+AAP+Paclitaxel	Pro-GRP+BPA+Sotorasib	0.1815	0	0	****
Pro-GRP+AAP+Paclitaxel	Pro-GRP+Paclitaxel	0.2246	0	0	****
Pro-GRP+AAP+Paclitaxel	Pro-GRP+Sotorasib	0.2544	0	0	****
Pro-GRP+AAP+Sotorasib	Pro-GRP+BPA	0.1205	0	0	****
Pro-GRP+AAP+Sotorasib	Pro-GRP+BPA+Paclitaxel	-0.0256	0	0	****
Pro-GRP+AAP+Sotorasib	Pro-GRP+BPA+Sotorasib	0.0691	0	0	****
Pro-GRP+AAP+Sotorasib	Pro-GRP+Paclitaxel	0.1122	0	0	****
Pro-GRP+AAP+Sotorasib	Pro-GRP+Sotorasib	0.142	0	0	****
Pro-GRP+BPA	Pro-GRP+BPA+Paclitaxel	-0.1461	0	0	****
Pro-GRP+BPA	Pro-GRP+BPA+Sotorasib	-0.0514	0	0	****
Pro-GRP+BPA	Pro-GRP+Paclitaxel	-0.0083	0	0	****
Pro-GRP+BPA	Pro-GRP+Sotorasib	0.0214	0	0	****
Pro-GRP+BPA+Paclitaxel	Pro-GRP+BPA+Sotorasib	0.0947	0	0	****
Pro-GRP+BPA+Paclitaxel	Pro-GRP+Paclitaxel	0.1378	0	0	****
Pro-GRP+BPA+Paclitaxel	Pro-GRP+Sotorasib	0.1676	0	0	****
Pro-GRP+BPA+Sotorasib	Pro-GRP+Paclitaxel	0.0431	0	0	****
Pro-GRP+BPA+Sotorasib	Pro-GRP+Sotorasib	0.0729	0	0	****
Pro-GRP+Paclitaxel	Pro-GRP+Sotorasib	0.0298	0	0	****

SOD Complexes

Group1	Group2	Mean_Diff (Δ)	p_value	p_adj	Significance
SOD	SOD+AAP	0.461	0	0	****
SOD	SOD+AAP+Paclitaxel	-0.0417	0	0	****
SOD	SOD+AAP+Sotorasib	-0.0848	0	0	****
SOD	SOD+BPA	0.159	0	0	****
SOD	SOD+BPA+Paclitaxel	-0.5082	0	0	****
SOD	SOD+BPA+Sotorasib	-0.5823	0	0	****
SOD	SOD+Paclitaxel	-0.6953	0	0	****
SOD	SOD+Sotorasib	-0.3953	0	0	****
SOD+AAP	SOD+AAP+Paclitaxel	-0.5027	0	0	****
SOD+AAP	SOD+AAP+Sotorasib	-0.5459	0	0	****
SOD+AAP	SOD+BPA	-0.302	0	0	****
SOD+AAP	SOD+BPA+Paclitaxel	-0.9693	0	0	****
SOD+AAP	SOD+BPA+Sotorasib	-1.0433	0	0	****
SOD+AAP	SOD+Paclitaxel	-1.1563	0	0	****
SOD+AAP	SOD+Sotorasib	-0.8563	0	0	****
SOD+AAP+Paclitaxel	SOD+AAP+Sotorasib	-0.0431	0	0	****
SOD+AAP+Paclitaxel	SOD+BPA	0.2007	0	0	****
SOD+AAP+Paclitaxel	SOD+BPA+Paclitaxel	-0.4665	0	0	****
SOD+AAP+Paclitaxel	SOD+BPA+Sotorasib	-0.5406	0	0	****
SOD+AAP+Paclitaxel	SOD+Paclitaxel	-0.6536	0	0	****
SOD+AAP+Paclitaxel	SOD+Sotorasib	-0.3536	0	0	****
SOD+AAP+Sotorasib	SOD+BPA	0.2438	0	0	****

SOD+AAP+Sotorasib	SOD+BPA+Paclitaxel	-0.4234	0	0	****
SOD+AAP+Sotorasib	SOD+BPA+Sotorasib	-0.4974	0	0	****
SOD+AAP+Sotorasib	SOD+Paclitaxel	-0.6105	0	0	****
SOD+AAP+Sotorasib	SOD+Sotorasib	-0.3104	0	0	****
SOD+BPA	SOD+BPA+Paclitaxel	-0.6673	0	0	****
SOD+BPA	SOD+BPA+Sotorasib	-0.7413	0	0	****
SOD+BPA	SOD+Paclitaxel	-0.8543	0	0	****
SOD+BPA	SOD+Sotorasib	-0.5543	0	0	****
SOD+BPA+Paclitaxel	SOD+BPA+Sotorasib	-0.074	0	0	****
SOD+BPA+Paclitaxel	SOD+Paclitaxel	-0.1871	0	0	****

****** for p < 0.0001 and *** for p < 0.001**

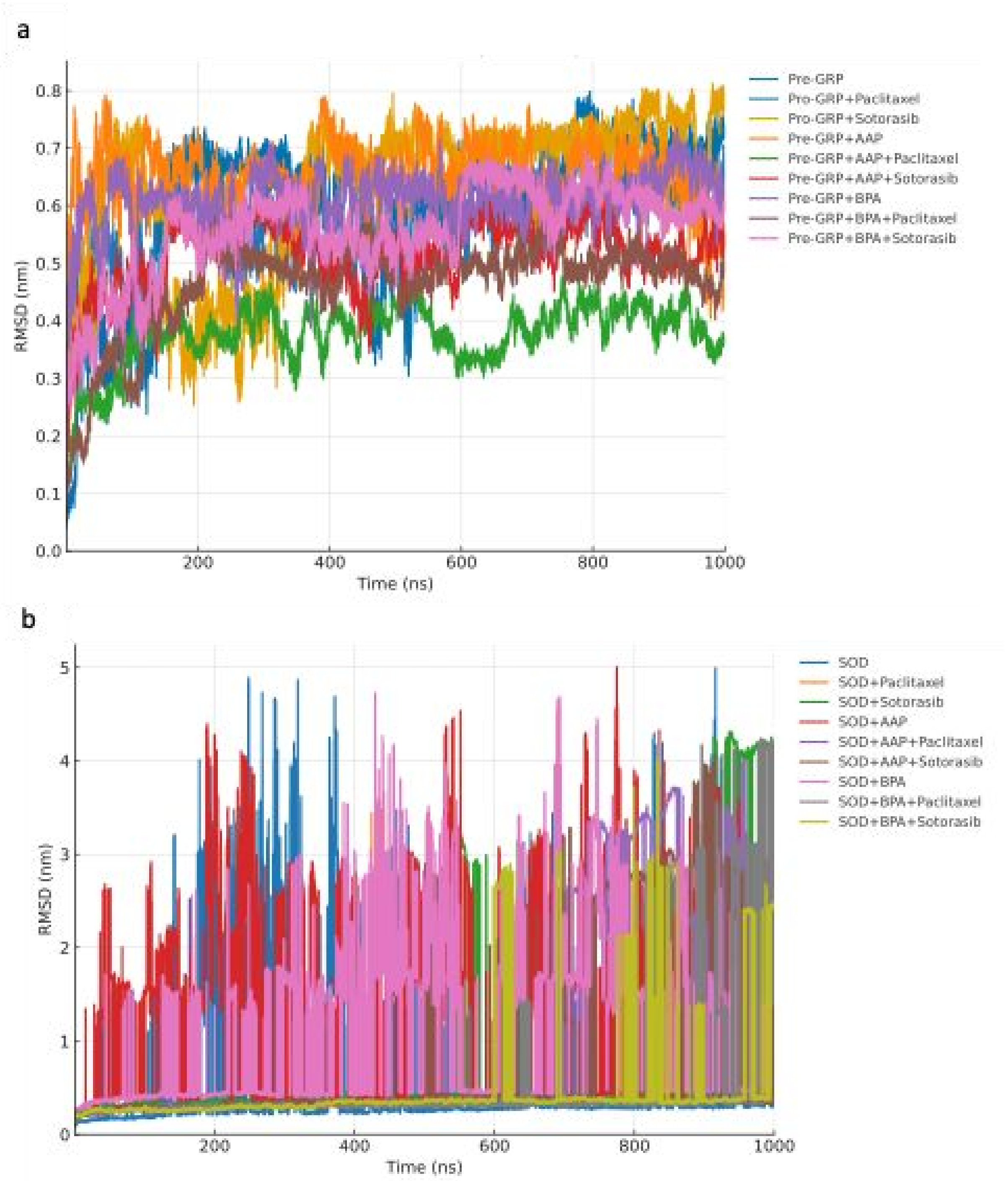

Sup. Figure 2. RMSD analysis of different molecular systems over a 1000 ns simulation. (a) RMSD profiles of the Pre-GRP system with various treatments, indicating stable fluctuations within the range of 0.5–1.2 nm. (b) RMSD profiles of the SOD system with different treatments, showing significantly higher deviations and instability, particularly in untreated and BPA-treated conditions.

Sup. Table 4. Pairwise Statistical Comparisons of Total Energy Between Pro-GRP and SOD Complexes

Pro-GRP complexes					
Group1	Group2	Mean Diff	p-value	p_adj	Significance
Pro-GRP	Pro-GRP+AAP	7817.3606	0	0	****
Pro-GRP	Pro-GRP+AAP+Paclitaxel	21295.6413	0	0	****
Pro-GRP	Pro-GRP+AAP+Sotorasib	-187703.72	0	0	****
Pro-GRP	Pro-GRP+BPA	8510.4521	0	0	****
Pro-GRP	Pro-GRP+BPA+Paclitaxel	-171228.07	0	0	****
Pro-GRP	Pro-GRP+BPA+Sotorasib	8620.8629	0	0	****
Pro-GRP	Pro-GRP+Paclitaxel	-14843.017	0	0	****
Pro-GRP	Pro-GRP+Sotorasib	7510.2357	0	0	****
Pro-GRP+AAP	Pro-GRP+AAP+Paclitaxel	13478.2806	0	0	****
Pro-GRP+AAP	Pro-GRP+AAP+Sotorasib	-195521.08	0	0	****
Pro-GRP+AAP	Pro-GRP+BPA	693.0915	0.9869	0.9869	ns
Pro-GRP+AAP	Pro-GRP+BPA+Paclitaxel	-179045.43	0	0	****
Pro-GRP+AAP	Pro-GRP+BPA+Sotorasib	803.5023	0.9669	0.9669	ns
Pro-GRP+AAP	Pro-GRP+Paclitaxel	-22660.378	0	0	****
Pro-GRP+AAP	Pro-GRP+Sotorasib	-307.1249	1	1	ns
Pro-GRP+AAP+Paclitaxel	Pro-GRP+AAP+Sotorasib	-208999.36	0	0	****
Pro-GRP+AAP+Paclitaxel	Pro-GRP+BPA	-12785.189	0	0	****
Pro-GRP+AAP+Paclitaxel	Pro-GRP+BPA+Paclitaxel	-192523.71	0	0	****
Pro-GRP+AAP+Paclitaxel	Pro-GRP+BPA+Sotorasib	-12674.778	0	0	****
Pro-GRP+AAP+Paclitaxel	Pro-GRP+Paclitaxel	-36138.658	0	0	****
Pro-GRP+AAP+Paclitaxel	Pro-GRP+Sotorasib	-13785.406	0	0	****
Pro-GRP+AAP+Sotorasib	Pro-GRP+BPA	196214.171	0	0	****
Pro-GRP+AAP+Sotorasib	Pro-GRP+BPA+Paclitaxel	16475.6464	0	0	****
Pro-GRP+AAP+Sotorasib	Pro-GRP+BPA+Sotorasib	196324.582	0	0	****
Pro-GRP+AAP+Sotorasib	Pro-GRP+Paclitaxel	172860.702	0	0	****
Pro-GRP+AAP+Sotorasib	Pro-GRP+Sotorasib	195213.955	0	0	****

Pro-GRP+BPA	Pro-GRP+BPA+Paclitaxel	-179738.52	0	0	****
Pro-GRP+BPA	Pro-GRP+BPA+Sotorasib	110.4108	1	1	ns
Pro-GRP+BPA	Pro-GRP+Paclitaxel	-23353.469	0	0	****
Pro-GRP+BPA	Pro-GRP+Sotorasib	-1000.2164	0.8872	0.8872	ns
Pro-GRP+BPA+Paclitaxel	Pro-GRP+BPA+Sotorasib	179848.936	0	0	****
Pro-GRP+BPA+Paclitaxel	Pro-GRP+Paclitaxel	156385.056	0	0	****
Pro-GRP+BPA+Paclitaxel	Pro-GRP+Sotorasib	178738.308	0	0	****
Pro-GRP+BPA+Sotorasib	Pro-GRP+Paclitaxel	-23463.88	0	0	****
Pro-GRP+BPA+Sotorasib	Pro-GRP+Sotorasib	-1110.6273	0.8128	0.8128	ns
Pro-GRP+Paclitaxel	Pro-GRP+Sotorasib	22353.2529	0	0	****
**SOD Complexes**					
**Group1**	**Group2**	**Mean_Diff**	**p_value**	**p_adj**	**Significance**
SOD	SOD+AAP	-10973.062	0	0	****
SOD	SOD+AAP+Paclitaxel	-53250.542	0	0	****
SOD	SOD+AAP+Sotorasib	-26405.585	0	0	****
SOD	SOD+BPA	-11118.827	0	0	****
SOD	SOD+BPA+Paclitaxel	-67146.684	0	0	****
SOD	SOD+BPA+Sotorasib	-21724.141	0	0	****
SOD	SOD+Paclitaxel	-44378.278	0	0	****
SOD	SOD+Sotorasib	-21847.091	0	0	****
SOD+AAP	SOD+AAP+Paclitaxel	-42277.48	0	0	****
SOD+AAP	SOD+AAP+Sotorasib	-15432.523	0	0	****
SOD+AAP	SOD+BPA	-145.7649	0.8442	0.8442	ns
SOD+AAP	SOD+BPA+Paclitaxel	-56173.622	0	0	****
SOD+AAP	SOD+BPA+Sotorasib	-10751.079	0	0	****
SOD+AAP	SOD+Paclitaxel	-33405.216	0	0	****
SOD+AAP	SOD+Sotorasib	-10874.029	0	0	****
SOD+AAP+Paclitaxel	SOD+AAP+Sotorasib	26844.9565	0	0	****
SOD+AAP+Paclitaxel	SOD+BPA	42131.7149	0	0	****
SOD+AAP+Paclitaxel	SOD+BPA+Paclitaxel	-13896.143	0	0	****
SOD+AAP+Paclitaxel	SOD+BPA+Sotorasib	31526.4013	0	0	****
SOD+AAP+Paclitaxel	SOD+Paclitaxel	8872.2642	0	0	****
SOD+AAP+Paclitaxel	SOD+Sotorasib	31403.451	0	0	****
SOD+AAP+Sotorasib	SOD+BPA	15286.7583	0	0	****
SOD+AAP+Sotorasib	SOD+BPA+Paclitaxel	-40741.099	0	0	****

SOD+AAP+Sotorasib	SOD+BPA+Sotorasib	4681.4448	0	0	****
SOD+AAP+Sotorasib	SOD+Paclitaxel	-17972.692	0	0	****
SOD+AAP+Sotorasib	SOD+Sotorasib	4558.4945	0	0	****
SOD+BPA	SOD+BPA+Paclitaxel	-56027.857	0	0	****
SOD+BPA	SOD+BPA+Sotorasib	-10605.314	0	0	****
SOD+BPA	SOD+Paclitaxel	-33259.451	0	0	****
SOD+BPA	SOD+Sotorasib	-10728.264	0	0	****
SOD+BPA+Paclitaxel	SOD+BPA+Sotorasib	45422.5439	0	0	****
SOD+BPA+Paclitaxel	SOD+Paclitaxel	22768.4068	0	0	****
SOD+BPA+Paclitaxel	SOD+Sotorasib	45299.5936	0	0	****
SOD+BPA+Sotorasib	SOD+Paclitaxel	-22654.137	0	0	****
SOD+BPA+Sotorasib	SOD+Sotorasib	-122.9503	0.9357	0.9357	ns
SOD+Paclitaxel	SOD+Sotorasib	22531.1868	0	0	****

All pairwise comparisons show highly significant differences ($p < 0.001$; *).**

Sup. Table 5. Pairwise Statistical Comparisons of RMSF Between Pro-GRP and SOD Complexes

Pro-GRP complexes						
Group1	Group2	Mean Diff	t-value	p-value	p_adj	Significance
Pro-GRP	Pro-GRP+AAP	-0.0152	0.75128375	0.45500031	0.9985	NS
Pro-GRP	Pro-GRP+AAP+Paclitaxel	-0.1286	6.97640535	1.38E-09	0	***
Pro-GRP	Pro-GRP+AAP+Sotorasib	-0.0997	4.99743241	4.12E-06	0.0001	***
Pro-GRP	Pro-GRP+BPA	-0.0506	2.85043287	0.00573161	0.283	NS
Pro-GRP	Pro-GRP+BPA+Paclitaxel	-0.1176	4.62288324	1.68E-05	0	***
Pro-GRP	Pro-GRP+BPA+Sotorasib	-0.0498	2.51482264	0.01420909	0.3037	NS
Pro-GRP	Pro-GRP+Paclitaxel	-0.0394	2.10952859	0.0384765	0.6323	NS
Pro-GRP	Pro-GRP+Sotorasib	-0.0489	2.5836778	0.01186483	0.3289	NS
Pro-GRP+AAP	Pro-GRP+AAP+Paclitaxel	-0.1134	5.67448054	2.91E-07	0	***
Pro-GRP+AAP	Pro-GRP+AAP+Sotorasib	-0.0845	3.95063254	0.0001837	0.0023	**
Pro-GRP+AAP	Pro-GRP+BPA	-0.0354	1.82991144	0.07152112	0.7547	NS
Pro-GRP+AAP	Pro-GRP+BPA+Paclitaxel	-0.1024	3.85219906	0.00025646	0.0001	***
Pro-GRP+AAP	Pro-GRP+BPA+Sotorasib	-0.0347	1.62927663	0.10774688	0.7769	NS
Pro-GRP+AAP	Pro-GRP+Paclitaxel	-0.0242	1.1979352	0.23498271	0.9656	NS
Pro-GRP+AAP	Pro-GRP+Sotorasib	-0.0337	1.64984347	0.10345557	0.8015	NS
GRP+AAP+Paclitaxel	Pro-GRP+AAP+Sotorasib	0.0289	-1.4643623	0.14757174	0.9067	NS
GRP+AAP+Paclitaxel	Pro-GRP+BPA	0.078	-4.4535718	3.12E-05	0.0075	**
GRP+AAP+Paclitaxel	Pro-GRP+BPA+Paclitaxel	0.011	-0.4360305	0.66415637	0.9999	NS
GRP+AAP+Paclitaxel	Pro-GRP+BPA+Sotorasib	0.0788	-4.0200311	0.0001448	0.0065	**
GRP+AAP+Paclitaxel	Pro-GRP+Paclitaxel	0.0892	-4.8396406	7.49E-06	0.001	**
GRP+AAP+Paclitaxel	Pro-GRP+Sotorasib	0.0797	-4.2607549	6.24E-05	0.0056	**
GRP+AAP+Sotorasib	Pro-GRP+BPA	0.0491	-2.5698251	0.01230622	0.3243	NS
GRP+AAP+Sotorasib	Pro-GRP+BPA+Paclitaxel	-0.0179	0.67739354	0.50038904	0.9951	NS
GRP+AAP+Sotorasib	Pro-GRP+BPA+Sotorasib	0.0499	-2.3717575	0.02045811	0.3028	NS
GRP+AAP+Sotorasib	Pro-GRP+Paclitaxel	0.0603	-3.0232843	0.00349277	0.1002	NS
GRP+AAP+Sotorasib	Pro-GRP+Sotorasib	0.0508	-2.5143404	0.01422689	0.2788	NS
Pro-GRP+BPA	Pro-GRP+BPA+Paclitaxel	-0.067	2.70298431	0.00861762	0.0417	**
Pro-GRP+BPA	Pro-GRP+BPA+Sotorasib	0.0008	-0.041464	0.967044	1	NS
Pro-GRP+BPA	Pro-GRP+Paclitaxel	0.0112	-0.6325642	0.52907921	0.9998	NS
Pro-GRP+BPA	Pro-GRP+Sotorasib	0.0017	-0.0942601	0.92517182	1	NS
GRP+BPA+Paclitaxel	Pro-GRP+BPA+Sotorasib	0.0677	-2.577204	0.01206929	0.0372	**
GRP+BPA+Paclitaxel	Pro-GRP+Paclitaxel	0.0782	-3.0743221	0.00300713	0.0072	**
GRP+BPA+Paclitaxel	Pro-GRP+Sotorasib	0.0687	-2.679055	0.00919523	0.0326	**
GRP+BPA+Sotorasib	Pro-GRP+Paclitaxel	0.0104	-0.5272181	0.59970917	0.9999	NS
GRP+BPA+Sotorasib	Pro-GRP+Sotorasib	0.0009	-0.0455483	0.96379999	1	NS
ro-GRP+Paclitaxel	Pro-GRP+Sotorasib	-0.0095	0.50350504	0.61619009	1	NS

SOD Complexes						
Group1	Group2	Mean_Diff	t_value	p_value	p_adj	Significance

SOD_Chain.A	SOD_Chain.B	-0.010512121	0.7954456	4.27E-01	1.00E+00	NS
SOD_Chain.A	SOD.AAP_Chain.A	-0.237772294	16.27025907	3.06E-52	2.78E-50	***
SOD_Chain.A	SOD.AAP.Paclitaxel_Chain.A	0.391869913	10.00818086	1.16E-21	1.05E-19	***
SOD_Chain.A	SOD.AAP.Sotorasib_Chain.A	0.342111255	10.82050218	7.54E-25	6.86E-23	***
SOD_Chain.A	SOD.BPA_Chain.A	-0.032137446	2.44559538	1.46E-02	1.00E+00	NS
SOD_Chain.A	SOD.BPA.Paclitaxel_Chain.A	0.670345238	50.86362586	1.12E-269	1.02E-267	***
SOD_Chain.A	SOD.BPA.Sotorasib_Chain.A	0.943362987	72.44633643	0.00E+00	0.00E+00	***
SOD_Chain.B	SOD.AAP_Chain.B	-0.237337229	15.73545784	2.19E-49	1.99E-47	***
SOD_Chain.B	SOD.AAP.Paclitaxel_Chain.B	0.397594805	10.09571352	5.34E-22	4.86E-20	***
SOD_Chain.B	SOD.AAP.Sotorasib_Chain.B	0.328977706	10.45128902	1.87E-23	1.70E-21	***
SOD_Chain.B	SOD.BPA_Chain.B	-0.034490476	2.57472782	1.02E-02	9.27E-01	NS
SOD_Chain.B	SOD.BPA.Paclitaxel_Chain.B	0.672690476	50.0480951	4.47E-265	4.06E-263	***
SOD_Chain.B	SOD.BPA.Sotorasib_Chain.B	0.955221212	69.47273503	0.00E+00	0.00E+00	***
SOD.AAP_Chain.A	SOD.AAP_Chain.B	-0.010077056	0.61738156	5.37E-01	1.00E+00	NS
SOD.AAP_Chain.A	SOD.AAP.Paclitaxel_Chain.A	0.629642208	15.8437256	9.47E-47	8.62E-45	***
SOD.AAP_Chain.A	SOD.AAP.Sotorasib_Chain.A	0.57988355	17.93105535	1.06E-57	9.66E-56	***
SOD.AAP_Chain.A	SOD.BPA_Chain.A	0.205634848	13.89855249	6.83E-40	6.21E-38	***
SOD.AAP_Chain.B	SOD.AAP.Paclitaxel_Chain.B	0.634932035	15.89052244	5.08E-47	4.63E-45	***
SOD.AAP_Chain.B	SOD.AAP.Sotorasib_Chain.B	0.566314935	17.59144068	3.92E-56	3.57E-54	***
SOD.AAP_Chain.B	SOD.BPA_Chain.B	0.202846753	13.52322303	5.28E-38	4.80E-36	***
SOD.AAP.Paclitaxel_Chain.A	SOD.AAP.Paclitaxel_Chain.B	-0.004787229	0.08875215	9.29E-01	1.00E+00	NS
SOD.AAP.Paclitaxel_Chain.A	SOD.AAP.Sotorasib_Chain.A	-0.049758658	1.02309697	3.07E-01	1.00E+00	NS
SOD.AAP.Paclitaxel_Chain.A	SOD.BPA.Paclitaxel_Chain.A	0.278475325	7.09745996	4.20E-12	3.83E-10	***
SOD.AAP.Paclitaxel_Chain.B	SOD.AAP.Sotorasib_Chain.B	-0.0686171	1.41250412	1.58E-01	1.00E+00	NS
SOD.AAP.Paclitaxel_Chain.B	SOD.BPA.Paclitaxel_Chain.B	0.275095671	6.98812342	8.62E-12	7.84E-10	***
SOD.AAP.Sotorasib_Chain.A	SOD.AAP.Sotorasib_Chain.B	-0.023645671	0.5549035	5.79E-01	1.00E+00	NS
SOD.AAP.Sotorasib_Chain.A	SOD.BPA.Sotorasib_Chain.A	0.601251732	18.99573391	3.66E-62	3.33E-60	***
SOD.AAP.Sotorasib_Chain.B	SOD.BPA.Sotorasib_Chain.B	0.626243506	19.82417033	1.14E-66	1.04E-64	***
SOD.BPA_Chain.A	SOD.BPA_Chain.B	-0.012865152	0.96568209	3.34E-01	1.00E+00	NS
SOD.BPA_Chain.A	SOD.BPA.Paclitaxel_Chain.A	0.702482684	52.50152932	2.87E-279	2.61E-277	***
SOD.BPA_Chain.A	SOD.BPA.Sotorasib_Chain.A	0.975500433	73.76237303	0.00E+00	0.00E+00	***
SOD.BPA_Chain.B	SOD.BPA.Paclitaxel_Chain.B	0.707180952	52.98192045	5.24E-282	4.77E-280	***
SOD.BPA_Chain.B	SOD.BPA.Sotorasib_Chain.B	0.989711688	72.46174789	0.00E+00	0.00E+00	***
SOD.BPA.Paclitaxel_Chain.A	SOD.BPA.Sotorasib_Chain.A	0.273017749	20.58494889	9.25E-78	8.42E-76	***
SOD.BPA.Paclitaxel_Chain.B	SOD.BPA.Sotorasib_Chain.A	0.281184632	21.22094532	1.10E-81	9.97E-80	***
SOD.BPA.Paclitaxel_Chain.B	SOD.BPA.Sotorasib_Chain.B	0.282530736	20.61870416	5.99E-78	5.45E-76	***
SOD.BPA.Sotorasib_Chain.A	SOD.BPA.Sotorasib_Chain.B	0.001346104	0.09924549	9.21E-01	1.00E+00	NS
SOD+Paclitaxel_Chain A	SOD+Sotorasib_Chain A	-0.8253558	-104.45846	0	0	***
SOD+Sotorasib_Chain A	SOD_Chain A	-0.5874268	-52.246982	8.14E-278	0	***

SOD+AAP_Chain A	SOD_Chain A	0.23777229	16.2702591	1.62E-52	0	***
SOD+AAP_Chain A	SOD+Paclitaxel_Chain A	1.65055498	134.953658	0	0	***
SOD+AAP_Chain A	SOD+Sotorasib_Chain A	0.82519913	62.8057775	0	0	***
SOD+AAP_Chain A	SOD+BPA_Chain A	0.20563485	13.8985525	5.18E-40	0	***
SOD+AAP_Chain A	SOD+BPA+Paclitaxel_Chain A	0.90811753	61.2374114	0	0	***
SOD+AAP_Chain A	SOD+BPA+Sotorasib_Chain A	1.18113528	80.4069819	0	0	***
SOD+BPA_Chain A	SOD_Chain A	0.03213745	2.44559538	0.0146474	0.9414	NS
SOD+BPA_Chain A	SOD+Paclitaxel_Chain A	1.44492013	138.586707	0	0	***
SOD+BPA_Chain A	SOD+Sotorasib_Chain A	0.61956429	53.9775613	1.19E-287	0	***
SOD+AAP+Paclitaxel_Chain A	SOD_Chain A	-0.3918699	-10.008181	1.87E-22	0	***
SOD+AAP+Paclitaxel_Chain A	SOD+Paclitaxel_Chain A	1.02091277	26.6353568	2.21E-116	0	***
SOD+AAP+Paclitaxel_Chain A	SOD+Sotorasib_Chain A	0.19555693	5.06248013	5.00E-07	0	***
SOD+AAP+Paclitaxel_Chain A	SOD+AAP_Chain A	-0.6296422	-15.843726	3.49E-50	0	***
SOD+AAP+Paclitaxel_Chain A	SOD+BPA_Chain A	-0.4240074	-10.810155	9.97E-26	0	***
SOD+AAP+Paclitaxel_Chain A	SOD+AAP+Sotorasib_Chain A	-0.0497587	-1.023097	0.30653036	0.5731	NS
SOD+AAP+Paclitaxel_Chain A	SOD+BPA+Paclitaxel_Chain A	0.27847532	7.09745996	2.53E-12	0	***
SOD+AAP+Paclitaxel_Chain A	SOD+BPA+Sotorasib_Chain A	0.55149307	14.0747272	6.72E-41	0	***
SOD+AAP+Sotorasib_Chain A	SOD_Chain A	-0.3421113	-10.820502	9.02E-26	0	***
SOD+AAP+Sotorasib_Chain A	SOD+Paclitaxel_Chain A	1.07067143	35.0025242	1.95E-171	0	***
SOD+AAP+Sotorasib_Chain A	SOD+Sotorasib_Chain A	0.24531558	7.92291126	6.66E-15	0	***
SOD+AAP+Sotorasib_Chain A	SOD+AAP_Chain A	-0.5798835	-17.931055	6.57E-62	0	***
SOD+AAP+Sotorasib_Chain A	SOD+BPA_Chain A	-0.3742487	-11.805489	4.72E-30	0	***
SOD+AAP+Sotorasib_Chain A	SOD+BPA+Paclitaxel_Chain A	0.32823398	10.3487862	8.04E-24	0	***
SOD+AAP+Sotorasib_Chain A	SOD+BPA+Sotorasib_Chain A	0.60125173	18.9957339	3.69E-68	0	***
SOD+BPA+Paclitaxel_Chain A	SOD_Chain A	-0.6703452	-50.863626	7.78E-270	0	***
SOD+BPA+Paclitaxel_Chain A	SOD+Paclitaxel_Chain A	0.74243745	70.881408	0	0	***
SOD+BPA+Paclitaxel_Chain A	SOD+Sotorasib_Chain A	-0.0829184	-7.1965101	1.28E-12	0.0306	**
SOD+BPA+Paclitaxel_Chain A	SOD+BPA_Chain A	-0.7024827	-52.501529	2.84E-279	0	***
SOD+BPA+Paclitaxel_Chain A	SOD+BPA+Sotorasib_Chain A	0.27301775	20.5849489	9.10E-78	0	***
SOD+BPA+Sotorasib_Chain A	SOD_Chain A	-0.943363	-72.446336	0	0	***
SOD+BPA+Sotorasib_Chain A	SOD+Paclitaxel_Chain A	0.4694197	45.6847252	4.79E-239	0	***
SOD+BPA+Sotorasib_Chain A	SOD+Sotorasib_Chain A	-0.3559361	-31.384122	1.16E-147	0	***
SOD+BPA+Sotorasib_Chain A	SOD+BPA_Chain A	-0.9755004	-73.762373	0	0	***
SOD+Paclitaxel_Chain B	SOD_Chain B	-1.4569662	-147.97072	0	0	***
SOD+Paclitaxel_Chain B	SOD+Sotorasib_Chain B	-0.8631805	-149.48761	0	0	***
SOD+Sotorasib_Chain B	SOD_Chain B	-0.5937857	-54.58426	4.61E-291	0	***
SOD+AAP_Chain B	SOD_Chain B	0.23733723	15.7354578	1.35E-49	0	***
SOD+AAP_Chain B	SOD+Paclitaxel_Chain B	1.69430346	141.933342	0	0	***
SOD+AAP_Chain B	SOD+Sotorasib_Chain B	0.83112294	64.9220149	0	0	***
SOD+AAP_Chain B	SOD+BPA_Chain B	0.20284675	13.523223	3.81E-38	0	***

SOD+AAP_Chain B	SOD+BPA+Paclitaxel_Chain B	0.91002771	60.5064475	3e-323	0	***
SOD+AAP_Chain B	SOD+BPA+Sotorasib_Chain B	1.19255844	77.8601069	0	0	***
SOD+BPA_Chain B	SOD_Chain B	0.03449048	2.57472782	0.01018685	0.9128	NS
SOD+BPA_Chain B	SOD+Paclitaxel_Chain B	1.49145671	153.464476	0	0	***
SOD+BPA_Chain B	SOD+Sotorasib_Chain B	0.62827619	58.3745042	6.466330763486e-312	0	***
SOD+AAP+Paclitaxel_Chain B	SOD_Chain B	-0.3975948	-10.095714	8.39E-23	0	***
SOD+AAP+Paclitaxel_Chain B	SOD+Paclitaxel_Chain B	1.05937143	27.668335	3.70E-123	0	***
SOD+AAP+Paclitaxel_Chain B	SOD+Sotorasib_Chain B	0.19619091	5.08707852	4.41E-07	0	***
SOD+AAP+Paclitaxel_Chain B	SOD+AAP_Chain B	-0.634932	-15.890522	1.94E-50	0	***
SOD+AAP+Paclitaxel_Chain B	SOD+BPA_Chain B	-0.4320853	-10.980344	1.90E-26	0	***
SOD+AAP+Paclitaxel_Chain B	SOD+AAP+Sotorasib_Chain B	-0.0686171	-1.4125041	0.15813902	0.1464	NS
SOD+AAP+Paclitaxel_Chain B	SOD+BPA+Paclitaxel_Chain B	0.27509567	6.98812342	5.34E-12	0	***
SOD+AAP+Paclitaxel_Chain B	SOD+BPA+Sotorasib_Chain B	0.55762641	14.1269093	3.66E-41	0	***
SOD+AAP+Sotorasib_Chain B	SOD_Chain B	-0.3289777	-10.451289	3.07E-24	0	***
SOD+AAP+Sotorasib_Chain B	SOD+Paclitaxel_Chain B	1.12798853	37.4786225	1.61E-187	0	***
SOD+AAP+Sotorasib_Chain B	SOD+Sotorasib_Chain B	0.26480801	8.69645741	1.55E-17	0	***
SOD+AAP+Sotorasib_Chain B	SOD+AAP_Chain B	-0.5663149	-17.591441	5.96E-60	0	***
SOD+AAP+Sotorasib_Chain B	SOD+BPA_Chain B	-0.3634682	-11.561607	5.75E-29	0	***
SOD+AAP+Sotorasib_Chain B	SOD+BPA+Paclitaxel_Chain B	0.34371277	10.9265149	3.22E-26	0	***
SOD+AAP+Sotorasib_Chain B	SOD+BPA+Sotorasib_Chain B	0.62624351	19.8241703	3.96E-73	0	***
SOD+BPA+Paclitaxel_Chain B	SOD_Chain B	-0.6726905	-50.048095	4.40E-265	0	***
SOD+BPA+Paclitaxel_Chain B	SOD+Paclitaxel_Chain B	0.78427576	80.1864063	0	0	***
SOD+BPA+Paclitaxel_Chain B	SOD+Sotorasib_Chain B	-0.0789048	-7.2932062	6.52E-13	0.049	**
SOD+BPA+Paclitaxel_Chain B	SOD+BPA_Chain B	-0.707181	-52.98192	5.17E-282	0	***
SOD+BPA+Paclitaxel_Chain B	SOD+BPA+Sotorasib_Chain B	0.28253074	20.6187042	5.65E-78	0	***
SOD+BPA+Sotorasib_Chain B	SOD_Chain B	-0.9552212	-69.472735	0	0	***
SOD+BPA+Sotorasib_Chain B	SOD+Paclitaxel_Chain B	0.50174502	49.1871322	5.02E-260	0	***
SOD+BPA+Sotorasib_Chain B	SOD+Sotorasib_Chain B	-0.3614355	-32.270608	1.67E-153	0	***
SOD+BPA+Sotorasib_Chain B	SOD+BPA_Chain B	-0.9897117	-72.461748	0	0	***

$p < 0.001$ → Highly Significant (***), $p < 0.05$ → Significant (**), and $p > 0.05$ → Not Significant (NS)

Sup. table 6. Pairwise Statistical Comparisons of Conformational Changes Between Complexes

Pro-GRP Complexes						
**SS_Type**	Group1	Group2	Mean_Diff	t_value	p_adj	Significance
**E**	Pro-GRP	Pro-GRP+AAP	1.11346154	2.50384034	0.04652249	*
**E**	Pro-GRP	Pro-GRP+AAP+Paclitaxel	1.11346154	2.50384034	0.04652249	*
**E**	Pro-GRP	Pro-GRP+AAP+Sotorasib	1.11012821	2.49633193	0.04652249	*
**E**	Pro-GRP	Pro-GRP+BPA	-0.9798718	-1.0805846	0.34209174	NS
**E**	Pro-GRP	Pro-GRP+BPA+Paclitaxel	1.11346154	2.50384034	0.04652249	*
**E**	Pro-GRP	Pro-GRP+BPA+Sotorasib	-0.9798718	-1.0805846	0.34209174	NS
**E**	Pro-GRP	Pro-GRP+Paclitaxel	0.99346154	2.2261962	0.06084383	NS
**E**	Pro-GRP	Pro-GRP+Sotorasib	0.34387821	0.66996244	0.57480659	NS
**E**	Pro-GRP+AAP	Pro-GRP+AAP+Paclitaxel	0		1	NS
**E**	Pro-GRP+AAP	Pro-GRP+AAP+Sotorasib	-0.0033333	-2.3452079	0.05374259	NS
**E**	Pro-GRP+AAP	Pro-GRP+BPA	-2.0933333	-2.6488918	0.04652249	*
**E**	Pro-GRP+AAP	Pro-GRP+BPA+Paclitaxel	0		1	NS
**E**	Pro-GRP+AAP	Pro-GRP+BPA+Sotorasib	-2.0933333	-2.6488918	0.04652249	*
**E**	Pro-GRP+AAP	Pro-GRP+Paclitaxel	-0.12	-3.2206699	0.04652249	*
**E**	Pro-GRP+AAP	Pro-GRP+Sotorasib	-0.7695833	-3.0025045	0.04652249	*
**E**	Pro-GRP+AAP+Paclitaxel	Pro-GRP+AAP+Sotorasib	-0.0033333	-2.3452079	0.05374259	NS
**E**	Pro-GRP+AAP+Paclitaxel	Pro-GRP+BPA	-2.0933333	-2.6488918	0.04652249	*
**E**	Pro-GRP+AAP+Paclitaxel	Pro-GRP+BPA+Paclitaxel	0		1	NS
**E**	Pro-GRP+AAP+Paclitaxel	Pro-GRP+BPA+Sotorasib	-2.0933333	-2.6488918	0.04652249	*
**E**	Pro-GRP+AAP+Paclitaxel	Pro-GRP+Paclitaxel	-0.12	-3.2206699	0.04652249	*
**E**	Pro-GRP+AAP+Paclitaxel	Pro-GRP+Sotorasib	-0.7695833	-3.0025045	0.04652249	*
**E**	Pro-GRP+AAP+Sotorasib	Pro-GRP+BPA	-2.09	-2.6446696	0.04652249	*
**E**	Pro-GRP+AAP+Sotorasib	Pro-GRP+BPA+Paclitaxel	0.00333333	2.34520788	0.05374259	NS
**E**	Pro-GRP+AAP+Sotorasib	Pro-GRP+BPA+Sotorasib	-2.09	-2.6446696	0.04652249	*
**E**	Pro-GRP+AAP+Sotorasib	Pro-GRP+Paclitaxel	-0.1166667	-3.1289311	0.04652249	*
**E**	Pro-GRP+AAP+Sotorasib	Pro-GRP+Sotorasib	-0.76625	-2.9894536	0.04652249	*

**E**	Pro-GRP+BPA	Pro-GRP+BPA+Paclitaxel	2.09333333	2.64889181	0.04652249	*
**E**	Pro-GRP+BPA	Pro-GRP+BPA+Sotorasib	0	0	1	NS
**E**	Pro-GRP+BPA	Pro-GRP+Paclitaxel	1.97333333	2.49427378	0.04652249	*
**E**	Pro-GRP+BPA	Pro-GRP+Sotorasib	1.32375	1.59335418	0.16706396	NS
**E**	Pro-GRP+BPA+Paclitaxel	Pro-GRP+BPA+Sotorasib	-2.0933333	-2.6488918	0.04652249	*
**E**	Pro-GRP+BPA+Paclitaxel	Pro-GRP+Paclitaxel	-0.12	-3.2206699	0.04652249	*
**E**	Pro-GRP+BPA+Paclitaxel	Pro-GRP+Sotorasib	-0.7695833	-3.0025045	0.04652249	*
**E**	Pro-GRP+BPA+Sotorasib	Pro-GRP+Paclitaxel	1.97333333	2.49427378	0.04652249	*
**E**	Pro-GRP+BPA+Sotorasib	Pro-GRP+Sotorasib	1.32375	1.59335418	0.16706396	NS
**E**	Pro-GRP+Paclitaxel	Pro-GRP+Sotorasib	-0.6495833	-2.5079686	0.04652249	*
**G**	Pro-GRP	Pro-GRP+AAP	1.83592949	1.69946291	0.24084057	NS
**G**	Pro-GRP	Pro-GRP+AAP+Paclitaxel	1.83592949	1.69946291	0.24084057	NS
**G**	Pro-GRP	Pro-GRP+AAP+Sotorasib	1.47217949	1.25559323	0.36613987	NS
**G**	Pro-GRP	Pro-GRP+BPA	2.77634615	2.83589234	0.17423339	NS
**G**	Pro-GRP	Pro-GRP+BPA+Paclitaxel	2.30092949	2.22972891	0.20778579	NS
**G**	Pro-GRP	Pro-GRP+BPA+Sotorasib	2.77634615	2.83589234	0.17423339	NS
**G**	Pro-GRP	Pro-GRP+Paclitaxel	2.77967949	2.84120931	0.17423339	NS
**G**	Pro-GRP	Pro-GRP+Sotorasib	2.40009615	2.37909571	0.20778579	NS
**G**	Pro-GRP+AAP	Pro-GRP+AAP+Paclitaxel	0	0	1	NS
**G**	Pro-GRP+AAP	Pro-GRP+AAP+Sotorasib	-0.36375	-0.4469296	0.76612738	NS
**G**	Pro-GRP+AAP	Pro-GRP+BPA	0.94041667	1.89564717	0.20778579	NS
**G**	Pro-GRP+AAP	Pro-GRP+BPA+Paclitaxel	0.465	0.78314999	0.54941258	NS
**G**	Pro-GRP+AAP	Pro-GRP+BPA+Sotorasib	0.94041667	1.89564717	0.20778579	NS
**G**	Pro-GRP+AAP	Pro-GRP+Paclitaxel	0.94375	1.90737037	0.20778579	NS
**G**	Pro-GRP+AAP	Pro-GRP+Sotorasib	0.56416667	1.02088619	0.42802362	NS
**G**	Pro-GRP+AAP+Paclitaxel	Pro-GRP+AAP+Sotorasib	-0.36375	-0.4469296	0.76612738	NS
**G**	Pro-GRP+AAP+Paclitaxel	Pro-GRP+BPA	0.94041667	1.89564717	0.20778579	NS
**G**	Pro-GRP+AAP+Paclitaxel	Pro-GRP+BPA+Paclitaxel	0.465	0.78314999	0.54941258	NS

**G**	Pro-GRP+AAP+Paclitaxel	Pro-GRP+BPA+Sotorasib	0.94041667	1.89564717	0.20778579	NS
**G**	Pro-GRP+AAP+Paclitaxel	Pro-GRP+Paclitaxel	0.94375	1.90737037	0.20778579	NS
**G**	Pro-GRP+AAP+Paclitaxel	Pro-GRP+Sotorasib	0.56416667	1.02088619	0.42802362	NS
**G**	Pro-GRP+AAP+Sotorasib	Pro-GRP+BPA	1.30416667	1.93595905	0.20778579	NS
**G**	Pro-GRP+AAP+Sotorasib	Pro-GRP+BPA+Paclitaxel	0.82875	1.1072186	0.40861281	NS
**G**	Pro-GRP+AAP+Sotorasib	Pro-GRP+BPA+Sotorasib	1.30416667	1.93595905	0.20778579	NS
**G**	Pro-GRP+AAP+Sotorasib	Pro-GRP+Paclitaxel	1.3075	1.94367094	0.20778579	NS
**G**	Pro-GRP+AAP+Sotorasib	Pro-GRP+Sotorasib	0.92791667	1.29541767	0.36613987	NS
**G**	Pro-GRP+BPA	Pro-GRP+BPA+Paclitaxel	-0.4754167	-1.2530736	0.36613987	NS
**G**	Pro-GRP+BPA	Pro-GRP+BPA+Sotorasib	0	0	1	NS
**G**	Pro-GRP+BPA	Pro-GRP+Paclitaxel	0.00333333	0.01751555	1	NS
**G**	Pro-GRP+BPA	Pro-GRP+Sotorasib	-0.37625	-1.2093615	0.36613987	NS
**G**	Pro-GRP+BPA+Paclitaxel	Pro-GRP+BPA+Sotorasib	0.47541667	1.25307363	0.36613987	NS
**G**	Pro-GRP+BPA+Paclitaxel	Pro-GRP+Paclitaxel	0.47875	1.2675504	0.36613987	NS
**G**	Pro-GRP+BPA+Paclitaxel	Pro-GRP+Sotorasib	0.09916667	0.21997395	0.93153178	NS
**G**	Pro-GRP+BPA+Sotorasib	Pro-GRP+Paclitaxel	0.00333333	0.01751555	1	NS
**G**	Pro-GRP+BPA+Sotorasib	Pro-GRP+Sotorasib	-0.37625	-1.2093615	0.36613987	NS
**G**	Pro-GRP+Paclitaxel	Pro-GRP+Sotorasib	-0.3795833	-1.2282859	0.36613987	NS
**H**	Pro-GRP	Pro-GRP+AAP	-23.873301	-2.599343	0.12058444	NS
**H**	Pro-GRP	Pro-GRP+AAP+Paclitaxel	-23.873301	-2.599343	0.12058444	NS
**H**	Pro-GRP	Pro-GRP+AAP+Sotorasib	-28.812051	-2.8270363	0.12058444	NS
**H**	Pro-GRP	Pro-GRP+BPA	-6.3333013	-2.2430967	0.12058444	NS
**H**	Pro-GRP	Pro-GRP+BPA+Paclitaxel	-21.699551	-2.2399551	0.12058444	NS
**H**	Pro-GRP	Pro-GRP+BPA+Sotorasib	-6.3333013	-2.2430967	0.12058444	NS
**H**	Pro-GRP	Pro-GRP+Paclitaxel	0.71753205	1.8508273	0.13576634	NS
**H**	Pro-GRP	Pro-GRP+Sotorasib	-4.6137179	-1.9068148	0.13576634	NS
**H**	Pro-GRP+AAP	Pro-GRP+AAP+Paclitaxel	0	0	1	NS

**H**	Pro-GRP+AAP	Pro-GRP+AAP+Sotorasib	-4.93875	-0.3602406	0.8124069	NS
**H**	Pro-GRP+AAP	Pro-GRP+BPA	17.54	1.82811733	0.13576634	NS
**H**	Pro-GRP+AAP	Pro-GRP+BPA+Paclitaxel	2.17375	0.16296087	0.92333763	NS
**H**	Pro-GRP+AAP	Pro-GRP+BPA+Sotorasib	17.54	1.82811733	0.13576634	NS
**H**	Pro-GRP+AAP	Pro-GRP+Paclitaxel	24.5908333	2.67934847	0.12058444	NS
**H**	Pro-GRP+AAP	Pro-GRP+Sotorasib	19.2595833	2.03083494	0.12824727	NS
**H**	Pro-GRP+AAP+Paclitaxel	Pro-GRP+AAP+Sotorasib	-4.93875	-0.3602406	0.8124069	NS
**H**	Pro-GRP+AAP+Paclitaxel	Pro-GRP+BPA	17.54	1.82811733	0.13576634	NS
**H**	Pro-GRP+AAP+Paclitaxel	Pro-GRP+BPA+Paclitaxel	2.17375	0.16296087	0.92333763	NS
**H**	Pro-GRP+AAP+Paclitaxel	Pro-GRP+BPA+Sotorasib	17.54	1.82811733	0.13576634	NS
**H**	Pro-GRP+AAP+Paclitaxel	Pro-GRP+Paclitaxel	24.5908333	2.67934847	0.12058444	NS
**H**	Pro-GRP+AAP+Paclitaxel	Pro-GRP+Sotorasib	19.2595833	2.03083494	0.12824727	NS
**H**	Pro-GRP+AAP+Sotorasib	Pro-GRP+BPA	22.47875	2.12811022	0.12058444	NS
**H**	Pro-GRP+AAP+Sotorasib	Pro-GRP+BPA+Paclitaxel	7.1125	0.50616966	0.77423223	NS
**H**	Pro-GRP+AAP+Sotorasib	Pro-GRP+BPA+Sotorasib	22.47875	2.12811022	0.12058444	NS
**H**	Pro-GRP+AAP+Sotorasib	Pro-GRP+Paclitaxel	29.5295833	2.89909245	0.12058444	NS
**H**	Pro-GRP+AAP+Sotorasib	Pro-GRP+Sotorasib	24.1983333	2.31296053	0.12058444	NS
**H**	Pro-GRP+BPA	Pro-GRP+BPA+Paclitaxel	-15.36625	-1.5248465	0.20209051	NS
**H**	Pro-GRP+BPA	Pro-GRP+BPA+Sotorasib	0	0	1	NS
**H**	Pro-GRP+BPA	Pro-GRP+Paclitaxel	7.05083333	2.51597234	0.12058444	NS
**H**	Pro-GRP+BPA	Pro-GRP+Sotorasib	1.71958333	0.46701355	0.77423223	NS
**H**	Pro-GRP+BPA+Paclitaxel	Pro-GRP+BPA+Sotorasib	15.36625	1.52484646	0.20209051	NS
**H**	Pro-GRP+BPA+Paclitaxel	Pro-GRP+Paclitaxel	22.4170833	2.31548327	0.12058444	NS
**H**	Pro-GRP+BPA+Paclitaxel	Pro-GRP+Sotorasib	17.0858333	1.71344493	0.16073784	NS
**H**	Pro-GRP+BPA+Sotorasib	Pro-GRP+Paclitaxel	7.05083333	2.51597234	0.12058444	NS
**H**	Pro-GRP+BPA+Sotorasib	Pro-GRP+Sotorasib	1.71958333	0.46701355	0.77423223	NS

**H**	Pro-GRP+Paclitaxel	Pro-GRP+Sotorasib	-5.33125	-2.2259774	0.12058444	NS
**S**	Pro-GRP	Pro-GRP+AAP	16.6736218	2.41478503	0.15082451	NS
**S**	Pro-GRP	Pro-GRP+AAP+Paclitaxel	16.6736218	2.41478503	0.15082451	NS
**S**	Pro-GRP	Pro-GRP+AAP+Sotorasib	16.6644551	2.48693449	0.15082451	NS
**S**	Pro-GRP	Pro-GRP+BPA	5.84612179	0.76022779	0.65502927	NS
**S**	Pro-GRP	Pro-GRP+BPA+Paclitaxel	13.5098718	1.83037696	0.22321998	NS
**S**	Pro-GRP	Pro-GRP+BPA+Sotorasib	5.84612179	0.76022779	0.65502927	NS
**S**	Pro-GRP	Pro-GRP+Paclitaxel	-4.6259615	-0.5078834	0.76551836	NS
**S**	Pro-GRP	Pro-GRP+Sotorasib	3.54903846	0.44281397	0.79444578	NS
**S**	Pro-GRP+AAP	Pro-GRP+AAP+Paclitaxel	0	0	1	NS
**S**	Pro-GRP+AAP	Pro-GRP+AAP+Sotorasib	-0.0091667	-0.0017773	1	NS
**S**	Pro-GRP+AAP	Pro-GRP+BPA	-10.8275	-1.6943876	0.22321998	NS
**S**	Pro-GRP+AAP	Pro-GRP+BPA+Paclitaxel	-3.16375	-0.5259975	0.76551836	NS
**S**	Pro-GRP+AAP	Pro-GRP+BPA+Sotorasib	-10.8275	-1.6943876	0.22321998	NS
**S**	Pro-GRP+AAP	Pro-GRP+Paclitaxel	-21.299583	-2.6488102	0.15082451	NS
**S**	Pro-GRP+AAP	Pro-GRP+Sotorasib	-13.124583	-1.9364747	0.22321998	NS
**S**	Pro-GRP+AAP+Paclitaxel	Pro-GRP+AAP+Sotorasib	-0.0091667	-0.0017773	1	NS
**S**	Pro-GRP+AAP+Paclitaxel	Pro-GRP+BPA	-10.8275	-1.6943876	0.22321998	NS
**S**	Pro-GRP+AAP+Paclitaxel	Pro-GRP+BPA+Paclitaxel	-3.16375	-0.5259975	0.76551836	NS
**S**	Pro-GRP+AAP+Paclitaxel	Pro-GRP+BPA+Sotorasib	-10.8275	-1.6943876	0.22321998	NS
**S**	Pro-GRP+AAP+Paclitaxel	Pro-GRP+Paclitaxel	-21.299583	-2.6488102	0.15082451	NS
**S**	Pro-GRP+AAP+Paclitaxel	Pro-GRP+Sotorasib	-13.124583	-1.9364747	0.22321998	NS
**S**	Pro-GRP+AAP+Sotorasib	Pro-GRP+BPA	-10.818333	-1.753599	0.22321998	NS
**S**	Pro-GRP+AAP+Sotorasib	Pro-GRP+BPA+Paclitaxel	-3.1545833	-0.5458298	0.76551836	NS
**S**	Pro-GRP+AAP+Sotorasib	Pro-GRP+BPA+Sotorasib	-10.818333	-1.753599	0.22321998	NS
**S**	Pro-GRP+AAP+Sotorasib	Pro-GRP+Paclitaxel	-21.290417	-2.7063941	0.15082451	NS
**S**	Pro-GRP+AAP+Sotorasib	Pro-GRP+Sotorasib	-13.115417	-1.9963764	0.22321998	NS

**S**	Pro-GRP+BPA	Pro-GRP+BPA+Paclitaxel	7.66375	1.11037915	0.45644434	NS
**S**	Pro-GRP+BPA	Pro-GRP+BPA+Sotorasib	0	0	1	NS
**S**	Pro-GRP+BPA	Pro-GRP+Paclitaxel	-10.472083	-1.2002841	0.43925082	NS
**S**	Pro-GRP+BPA	Pro-GRP+Sotorasib	-2.2970833	-0.3032086	0.86017721	NS
**S**	Pro-GRP+BPA+Paclitaxel	Pro-GRP+BPA+Sotorasib	-7.66375	-1.1103791	0.45644434	NS
**S**	Pro-GRP+BPA+Paclitaxel	Pro-GRP+Paclitaxel	-18.135833	-2.1453527	0.22321998	NS
**S**	Pro-GRP+BPA+Paclitaxel	Pro-GRP+Sotorasib	-9.9608333	-1.3716322	0.36894712	NS
**S**	Pro-GRP+BPA+Sotorasib	Pro-GRP+Paclitaxel	-10.472083	-1.2002841	0.43925082	NS
**S**	Pro-GRP+BPA+Sotorasib	Pro-GRP+Sotorasib	-2.2970833	-0.3032086	0.86017721	NS
**S**	Pro-GRP+Paclitaxel	Pro-GRP+Sotorasib	8.175	0.90709994	0.58648608	NS
**T**	Pro-GRP	Pro-GRP+AAP	-2.2767949	-0.3485352	0.99227014	NS
**T**	Pro-GRP	Pro-GRP+AAP+Paclitaxel	-2.2767949	-0.3485352	0.99227014	NS
**T**	Pro-GRP	Pro-GRP+AAP+Sotorasib	-0.8526282	-0.1850517	0.99227014	NS
**T**	Pro-GRP	Pro-GRP+BPA	-1.0909615	-0.1661185	0.99227014	NS
**T**	Pro-GRP	Pro-GRP+BPA+Paclitaxel	5.24445513	1.08731826	0.99227014	NS
**T**	Pro-GRP	Pro-GRP+BPA+Sotorasib	-1.0909615	-0.1661185	0.99227014	NS
**T**	Pro-GRP	Pro-GRP+Paclitaxel	1.48695513	0.23121966	0.99227014	NS
**T**	Pro-GRP	Pro-GRP+Sotorasib	3.09195513	0.53570175	0.99227014	NS
**T**	Pro-GRP+AAP	Pro-GRP+AAP+Paclitaxel	0	0	1	NS
**T**	Pro-GRP+AAP	Pro-GRP+AAP+Sotorasib	1.42416667	0.22385397	0.99227014	NS
**T**	Pro-GRP+AAP	Pro-GRP+BPA	1.18583333	0.15014557	0.99227014	NS
**T**	Pro-GRP+AAP	Pro-GRP+BPA+Paclitaxel	7.52125	1.15356497	0.99227014	NS
**T**	Pro-GRP+AAP	Pro-GRP+BPA+Sotorasib	1.18583333	0.15014557	0.99227014	NS
**T**	Pro-GRP+AAP	Pro-GRP+Paclitaxel	3.76375	0.48347448	0.99227014	NS
**T**	Pro-GRP+AAP	Pro-GRP+Sotorasib	5.36875	0.74053619	0.99227014	NS
**T**	Pro-GRP+AAP+Paclitaxel	Pro-GRP+AAP+Sotorasib	1.42416667	0.22385397	0.99227014	NS
**T**	Pro-GRP+AAP+Paclitaxel	Pro-GRP+BPA	1.18583333	0.15014557	0.99227014	NS
**T**	Pro-GRP+AAP+Paclitaxel	Pro-GRP+BPA+Paclitaxel	7.52125	1.15356497	0.99227014	NS

**T**	Pro-GRP+AAP+Paclitaxel	Pro-GRP+BPA+Sotorasib	1.18583333	0.15014557	0.99227014	NS
**T**	Pro-GRP+AAP+Paclitaxel	Pro-GRP+Paclitaxel	3.76375	0.48347448	0.99227014	NS
**T**	Pro-GRP+AAP+Paclitaxel	Pro-GRP+Sotorasib	5.36875	0.74053619	0.99227014	NS
**T**	Pro-GRP+AAP+Sotorasib	Pro-GRP+BPA	-0.2383333	-0.037252	1	NS
**T**	Pro-GRP+AAP+Sotorasib	Pro-GRP+BPA+Paclitaxel	6.09708333	1.32839099	0.99227014	NS
**T**	Pro-GRP+AAP+Sotorasib	Pro-GRP+BPA+Sotorasib	-0.2383333	-0.037252	1	NS
**T**	Pro-GRP+AAP+Sotorasib	Pro-GRP+Paclitaxel	2.33958333	0.37387147	0.99227014	NS
**T**	Pro-GRP+AAP+Sotorasib	Pro-GRP+Sotorasib	3.94458333	0.70714809	0.99227014	NS
**T**	Pro-GRP+BPA	Pro-GRP+BPA+Paclitaxel	6.33541667	0.96650508	0.99227014	NS
**T**	Pro-GRP+BPA	Pro-GRP+BPA+Sotorasib	0	0	1	NS
**T**	Pro-GRP+BPA	Pro-GRP+Paclitaxel	2.57791667	0.32990547	0.99227014	NS
**T**	Pro-GRP+BPA	Pro-GRP+Sotorasib	4.18291667	0.57447547	0.99227014	NS
**T**	Pro-GRP+BPA+Paclitaxel	Pro-GRP+BPA+Sotorasib	-6.3354167	-0.9665051	0.99227014	NS
**T**	Pro-GRP+BPA+Paclitaxel	Pro-GRP+Paclitaxel	-3.7575	-0.585439	0.99227014	NS
**T**	Pro-GRP+BPA+Paclitaxel	Pro-GRP+Sotorasib	-2.1525	-0.3738488	0.99227014	NS
**T**	Pro-GRP+BPA+Sotorasib	Pro-GRP+Paclitaxel	2.57791667	0.32990547	0.99227014	NS
**T**	Pro-GRP+BPA+Sotorasib	Pro-GRP+Sotorasib	4.18291667	0.57447547	0.99227014	NS
**T**	Pro-GRP+Paclitaxel	Pro-GRP+Sotorasib	1.605	0.22421056	0.99227014	NS
**P**	Pro-GRP	Pro-GRP+AAP	1.34371795	3.41360359	0.04289363	*
**P**	Pro-GRP	Pro-GRP+AAP+Paclitaxel	1.34371795	3.41360359	0.04289363	*
**P**	Pro-GRP	Pro-GRP+AAP+Sotorasib	0.95038462	2.16606314	0.1200129	NS
**P**	Pro-GRP	Pro-GRP+BPA	0.42038462	0.84269415	0.4742949	NS
**P**	Pro-GRP	Pro-GRP+BPA+Paclitaxel	-0.5271154	-0.5090519	0.69558936	NS
**P**	Pro-GRP	Pro-GRP+BPA+Sotorasib	0.42038462	0.84269415	0.4742949	NS
**P**	Pro-GRP	Pro-GRP+Paclitaxel	-0.0333654	-0.0585839	1	NS
**P**	Pro-GRP	Pro-GRP+Sotorasib	1.23871795	3.12079339	0.04289363	*
**P**	Pro-GRP+AAP	Pro-GRP+AAP+Paclitaxel	0	0	1	NS

**P**	Pro-GRP+AAP	Pro-GRP+AAP+Sotorasib	-0.3933333	-2.0150643	0.15024687	NS
**P**	Pro-GRP+AAP	Pro-GRP+BPA	-0.9233333	-3.0044085	0.04289363	*
**P**	Pro-GRP+AAP	Pro-GRP+BPA+Paclitaxel	-1.8708333	-1.9527952	0.15024687	NS
**P**	Pro-GRP+AAP	Pro-GRP+BPA+Sotorasib	-0.9233333	-3.0044085	0.04289363	*
**P**	Pro-GRP+AAP	Pro-GRP+Paclitaxel	-1.3770833	-3.3403477	0.04289363	*
**P**	Pro-GRP+AAP	Pro-GRP+Sotorasib	-0.105	-1.874137	0.15024687	NS
**P**	Pro-GRP+AAP+Paclitaxel	Pro-GRP+AAP+Sotorasib	-0.3933333	-2.0150643	0.15024687	NS
**P**	Pro-GRP+AAP+Paclitaxel	Pro-GRP+BPA	-0.9233333	-3.0044085	0.04289363	*
**P**	Pro-GRP+AAP+Paclitaxel	Pro-GRP+BPA+Paclitaxel	-1.8708333	-1.9527952	0.15024687	NS
**P**	Pro-GRP+AAP+Paclitaxel	Pro-GRP+BPA+Sotorasib	-0.9233333	-3.0044085	0.04289363	*
**P**	Pro-GRP+AAP+Paclitaxel	Pro-GRP+Paclitaxel	-1.3770833	-3.3403477	0.04289363	*
**P**	Pro-GRP+AAP+Paclitaxel	Pro-GRP+Sotorasib	-0.105	-1.874137	0.15024687	NS
**P**	Pro-GRP+AAP+Sotorasib	Pro-GRP+BPA	-0.53	-1.4587142	0.24197471	NS
**P**	Pro-GRP+AAP+Sotorasib	Pro-GRP+BPA+Paclitaxel	-1.4775	-1.5116084	0.24197471	NS
**P**	Pro-GRP+AAP+Sotorasib	Pro-GRP+BPA+Sotorasib	-0.53	-1.4587142	0.24197471	NS
**P**	Pro-GRP+AAP+Sotorasib	Pro-GRP+Paclitaxel	-0.98375	-2.159517	0.1200129	NS
**P**	Pro-GRP+AAP+Sotorasib	Pro-GRP+Sotorasib	0.28833333	1.42919975	0.25510699	NS
**P**	Pro-GRP+BPA	Pro-GRP+BPA+Paclitaxel	-0.9475	-0.9419922	0.4742949	NS
**P**	Pro-GRP+BPA	Pro-GRP+BPA+Sotorasib	0	0	1	NS
**P**	Pro-GRP+BPA	Pro-GRP+Paclitaxel	-0.45375	-0.883334	0.4742949	NS
**P**	Pro-GRP+BPA	Pro-GRP+Sotorasib	0.81833333	2.62685185	0.06764304	NS
**P**	Pro-GRP+BPA+Paclitaxel	Pro-GRP+BPA+Sotorasib	0.9475	0.94199216	0.4742949	NS
**P**	Pro-GRP+BPA+Paclitaxel	Pro-GRP+Paclitaxel	0.49375	0.47352769	0.70111051	NS
**P**	Pro-GRP+BPA+Paclitaxel	Pro-GRP+Sotorasib	1.76583333	1.84059069	0.15878422	NS
**P**	Pro-GRP+BPA+Sotorasib	Pro-GRP+Paclitaxel	-0.45375	-0.883334	0.4742949	NS
**P**	Pro-GRP+BPA+Sotorasib	Pro-GRP+Sotorasib	0.81833333	2.62685185	0.06764304	NS

**P**	Pro-GRP+Paclitaxel	Pro-GRP+Sotorasib	1.27208333	3.06232436	0.04289363	*
~	Pro-GRP	Pro-GRP+AAP	3.57689103	0.22758254	1	NS
~	Pro-GRP	Pro-GRP+AAP+Paclitaxel	3.57689103	0.22758254	1	NS
~	Pro-GRP	Pro-GRP+AAP+Sotorasib	8.05855769	0.52424064	1	NS
~	Pro-GRP	Pro-GRP+BPA	-0.006859	-0.0004922	1	NS
~	Pro-GRP	Pro-GRP+BPA+Paclitaxel	-1.6001923	-0.1053495	1	NS
~	Pro-GRP	Pro-GRP+BPA+Sotorasib	-0.006859	-0.0004922	1	NS
~	Pro-GRP	Pro-GRP+Paclitaxel	-2.078109	-0.1471352	1	NS
~	Pro-GRP	Pro-GRP+Sotorasib	-2.0722756	-0.150937	1	NS
~	Pro-GRP+AAP	Pro-GRP+AAP+Paclitaxel	0	0	1	NS
~	Pro-GRP+AAP	Pro-GRP+AAP+Sotorasib	4.48166667	0.25005243	1	NS
~	Pro-GRP+AAP	Pro-GRP+BPA	-3.58375	-0.2144951	1	NS
~	Pro-GRP+AAP	Pro-GRP+BPA+Paclitaxel	-5.1770833	-0.2913936	1	NS
~	Pro-GRP+AAP	Pro-GRP+BPA+Sotorasib	-3.58375	-0.2144951	1	NS
~	Pro-GRP+AAP	Pro-GRP+Paclitaxel	-5.655	-0.3353157	1	NS
~	Pro-GRP+AAP	Pro-GRP+Sotorasib	-5.6491667	-0.3416323	1	NS
~	Pro-GRP+AAP+Paclitaxel	Pro-GRP+AAP+Sotorasib	4.48166667	0.25005243	1	NS
~	Pro-GRP+AAP+Paclitaxel	Pro-GRP+BPA	-3.58375	-0.2144951	1	NS
~	Pro-GRP+AAP+Paclitaxel	Pro-GRP+BPA+Paclitaxel	-5.1770833	-0.2913936	1	NS
~	Pro-GRP+AAP+Paclitaxel	Pro-GRP+BPA+Sotorasib	-3.58375	-0.2144951	1	NS
~	Pro-GRP+AAP+Paclitaxel	Pro-GRP+Paclitaxel	-5.655	-0.3353157	1	NS
~	Pro-GRP+AAP+Paclitaxel	Pro-GRP+Sotorasib	-5.6491667	-0.3416323	1	NS
~	Pro-GRP+AAP+Sotorasib	Pro-GRP+BPA	-8.0654167	-0.4922832	1	NS
~	Pro-GRP+AAP+Sotorasib	Pro-GRP+BPA+Paclitaxel	-9.65875	-0.5531249	1	NS
~	Pro-GRP+AAP+Sotorasib	Pro-GRP+BPA+Sotorasib	-8.0654167	-0.4922832	1	NS
~	Pro-GRP+AAP+Sotorasib	Pro-GRP+Paclitaxel	-10.136667	-0.6127232	1	NS
~	Pro-GRP+AAP+Sotorasib	Pro-GRP+Sotorasib	-10.130833	-0.6250427	1	NS

~	Pro-GRP+BPA	Pro-GRP+BPA+Paclitaxel	-1.5933333	-0.0982776	1	NS
~	Pro-GRP+BPA	Pro-GRP+BPA+Sotorasib	0	0	1	NS
~	Pro-GRP+BPA	Pro-GRP+Paclitaxel	-2.07125	-0.1360978	1	NS
~	Pro-GRP+BPA	Pro-GRP+Sotorasib	-2.0654167	-0.1390521	1	NS
~	Pro-GRP+BPA+Paclitaxel	Pro-GRP+BPA+Sotorasib	1.59333333	0.09827756	1	NS
~	Pro-GRP+BPA+Paclitaxel	Pro-GRP+Paclitaxel	-0.4779167	-0.0291872	1	NS
~	Pro-GRP+BPA+Paclitaxel	Pro-GRP+Sotorasib	-0.4720833	-0.0294404	1	NS
~	Pro-GRP+BPA+Sotorasib	Pro-GRP+Paclitaxel	-2.07125	-0.1360978	1	NS
~	Pro-GRP+BPA+Sotorasib	Pro-GRP+Sotorasib	-2.0654167	-0.1390521	1	NS
~	Pro-GRP+Paclitaxel	Pro-GRP+Sotorasib	0.00583333	0.00038812	1	NS
**SOD Complexes_Chain A**						
**SS_Type**	Group1	Group2	Mean_Diff	t_value	p_adj	Significance
**E**	SOD	SOD+AAP	2.25627706	199.778536	0	***
**E**	SOD	SOD+AAP+Paclitaxel	2.09087662	177.262731	0	***
**E**	SOD	SOD+AAP+Sotorasib	2.69010823	226.607068	0	***
**E**	SOD	SOD+BPA	1.50869048	122.312354	0	***
**E**	SOD	SOD+BPA+Paclitaxel	1.9471645	136.060949	0	***
**E**	SOD	SOD+BPA+Sotorasib	2.03880952	175.514774	0	***
**E**	SOD	SOD+Paclitaxel	2.81758658	229.130186	0	***
**E**	SOD	SOD+Sotorasib	1.94047619	163.698995	0	***
**E**	SOD+AAP	SOD+AAP+Paclitaxel	-0.1654004	12.291639	1.31E-34	***
**E**	SOD+AAP	SOD+AAP+Sotorasib	0.43383117	32.0811982	1.15E-224	***
**E**	SOD+AAP	SOD+BPA	-0.7475866	53.6613757	0	***
**E**	SOD+AAP	SOD+BPA+Paclitaxel	-0.3091126	19.6784592	6.20E-86	***
**E**	SOD+AAP	SOD+BPA+Sotorasib	-0.2174675	16.351495	6.34E-60	***
**E**	SOD+AAP	SOD+Paclitaxel	0.56130952	40.3876345	0	***
**E**	SOD+AAP	SOD+Sotorasib	-0.3158009	23.3793033	1.80E-120	***
**E**	SOD+AAP+Paclitaxel	SOD+AAP+Sotorasib	0.5992316	42.9727878	0	***
**E**	SOD+AAP+Paclitaxel	SOD+BPA	-0.5821861	40.5956847	0	***
**E**	SOD+AAP+Paclitaxel	SOD+BPA+Paclitaxel	-0.1437121	8.94149671	4.52E-19	***
**E**	SOD+AAP+Paclitaxel	SOD+BPA+Sotorasib	-0.0520671	3.79278219	0.0001533	***
**E**	SOD+AAP+Paclitaxel	SOD+Paclitaxel	0.72670996	50.7885497	0	***
**E**	SOD+AAP+Paclitaxel	SOD+Sotorasib	-0.1504004	10.7971022	4.39E-27	***
**E**	SOD+AAP+Sotorasib	SOD+BPA	-1.1814177	82.0225673	0	***
**E**	SOD+AAP+Sotorasib	SOD+BPA+Paclitaxel	-0.7429437	46.0646908	0	***

E	SOD+AAP+Sotorasib	SOD+BPA+Sotorasib	-0.6512987	47.2188036	0	***
E	SOD+AAP+Sotorasib	SOD+Paclitaxel	0.12747835	8.87042552	8.31E-19	***
E	SOD+AAP+Sotorasib	SOD+Sotorasib	-0.749632	53.5680056	0	***
E	SOD+BPA	SOD+BPA+Paclitaxel	0.43847403	26.6186942	1.76E-155	***
E	SOD+BPA	SOD+BPA+Sotorasib	0.53011905	37.3479123	2.59E-303	***
E	SOD+BPA	SOD+Paclitaxel	1.3088961	88.7005842	0	***
E	SOD+BPA	SOD+Sotorasib	0.43178571	30.007416	6.38E-197	***
E	SOD+BPA+Paclitaxel	SOD+BPA+Sotorasib	0.09164502	5.74884917	9.54E-09	***
E	SOD+BPA+Paclitaxel	SOD+Paclitaxel	0.87042208	52.9322366	0	***
E	SOD+BPA+Paclitaxel	SOD+Sotorasib	-0.0066883	0.41502284	0.67812597	NS
E	SOD+BPA+Sotorasib	SOD+Paclitaxel	0.77877706	54.9937531	0	***
E	SOD+BPA+Sotorasib	SOD+Sotorasib	-0.0983333	7.13682377	1.05E-12	***
E	SOD+Paclitaxel	SOD+Sotorasib	-0.8771104	61.0934522	0	***
G	SOD	SOD+AAP	1.70628788	196.877791	0	***
G	SOD	SOD+AAP+Paclitaxel	1.7447619	203.539321	0	***
G	SOD	SOD+AAP+Sotorasib	1.5905303	181.02788	0	***
G	SOD	SOD+BPA	1.4116342	155.850029	0	***
G	SOD	SOD+BPA+Paclitaxel	1.01424242	106.805678	0	***
G	SOD	SOD+BPA+Sotorasib	1.40555195	160.833721	0	***
G	SOD	SOD+Paclitaxel	1.96511905	240.888423	0	***
G	SOD	SOD+Sotorasib	1.88541126	224.938175	0	***
G	SOD+AAP	SOD+AAP+Paclitaxel	0.03847403	5.63046902	1.85E-08	***
G	SOD+AAP	SOD+AAP+Sotorasib	-0.1157576	16.304376	1.16E-59	***
G	SOD+AAP	SOD+BPA	-0.2946537	39.6403092	0	***
G	SOD+AAP	SOD+BPA+Paclitaxel	-0.6920455	86.9222146	0	***
G	SOD+AAP	SOD+BPA+Sotorasib	-0.3007359	42.7084201	0	***
G	SOD+AAP	SOD+Paclitaxel	0.25883117	41.0476415	0	***
G	SOD+AAP	SOD+Sotorasib	0.17912338	27.168712	6.17E-162	***
G	SOD+AAP+Paclitaxel	SOD+AAP+Sotorasib	-0.1542316	22.0836774	8.68E-108	***
G	SOD+AAP+Paclitaxel	SOD+BPA	-0.3331277	45.4928642	0	***
G	SOD+AAP+Paclitaxel	SOD+BPA+Paclitaxel	-0.7305195	92.9585017	0	***
G	SOD+AAP+Paclitaxel	SOD+BPA+Sotorasib	-0.33921	48.9847135	0	***
G	SOD+AAP+Paclitaxel	SOD+Paclitaxel	0.22035714	35.6857813	2.82E-277	***
G	SOD+AAP+Paclitaxel	SOD+Sotorasib	0.14064935	21.7450403	1.38E-104	***
G	SOD+AAP+Sotorasib	SOD+BPA	-0.1788961	23.6259247	4.82E-123	***
G	SOD+AAP+Sotorasib	SOD+BPA+Paclitaxel	-0.5762879	71.2218924	0	***
G	SOD+AAP+Sotorasib	SOD+BPA+Sotorasib	-0.1849784	25.7342666	1.50E-145	***
G	SOD+AAP+Sotorasib	SOD+Paclitaxel	0.37458874	57.9077301	0	***
G	SOD+AAP+Sotorasib	SOD+Sotorasib	0.29488095	43.6915645	0	***
G	SOD+BPA	SOD+BPA+Paclitaxel	-0.3973918	47.3903178	0	***

**G**	SOD+BPA	SOD+BPA+Sotorasib	-0.0060823	0.80907866	0.41847152	NS
**G**	SOD+BPA	SOD+Paclitaxel	0.55348485	81.0020188	0	***
**G**	SOD+BPA	SOD+Sotorasib	0.47377706	66.7382389	0	***
**G**	SOD+BPA+Paclitaxel	SOD+BPA+Sotorasib	0.39130952	48.6676556	0	***
**G**	SOD+BPA+Paclitaxel	SOD+Paclitaxel	0.95087662	128.420131	0	***
**G**	SOD+BPA+Paclitaxel	SOD+Sotorasib	0.87116883	113.868872	0	***
**G**	SOD+BPA+Sotorasib	SOD+Paclitaxel	0.5595671	87.3667049	0	***
**G**	SOD+BPA+Sotorasib	SOD+Sotorasib	0.47985931	71.7501246	0	***
**G**	SOD+Paclitaxel	SOD+Sotorasib	-0.0797078	13.4913932	2.00E-41	***
**H**	SOD	SOD+AAP	0.12429654	11.6713119	1.86E-31	***
**H**	SOD	SOD+AAP+Paclitaxel	-0.5690801	64.3998578	0	***
**H**	SOD	SOD+AAP+Sotorasib	-1.0685281	132.652641	0	***
**H**	SOD	SOD+BPA	0.65335498	73.3009261	0	***
**H**	SOD	SOD+BPA+Paclitaxel	-1.1830087	150.359467	0	***
**H**	SOD	SOD+BPA+Sotorasib	-0.9312662	109.772896	0	***
**H**	SOD	SOD+Paclitaxel	-0.1815909	19.4010511	1.11E-83	***
**H**	SOD	SOD+Sotorasib	-1.5172078	188.121953	0	***
**H**	SOD+AAP	SOD+AAP+Paclitaxel	-0.6933766	67.2327848	0	***
**H**	SOD+AAP	SOD+AAP+Sotorasib	-1.1928247	123.58577	0	***
**H**	SOD+AAP	SOD+BPA	0.52905844	50.9747561	0	***
**H**	SOD+AAP	SOD+BPA+Paclitaxel	-1.3073052	137.667398	0	***
**H**	SOD+AAP	SOD+BPA+Sotorasib	-1.0555628	105.427847	0	***
**H**	SOD+AAP	SOD+Paclitaxel	-0.3058874	28.4156907	6.04E-177	***
**H**	SOD+AAP	SOD+Sotorasib	-1.6415043	169.92636	0	***
**H**	SOD+AAP+Paclitaxel	SOD+AAP+Sotorasib	-0.4994481	65.6789329	0	***
**H**	SOD+AAP+Paclitaxel	SOD+BPA	1.22243506	143.677057	0	***
**H**	SOD+AAP+Paclitaxel	SOD+BPA+Paclitaxel	-0.6139286	82.8984749	0	***
**H**	SOD+AAP+Paclitaxel	SOD+BPA+Sotorasib	-0.3621861	44.9536457	0	***
**H**	SOD+AAP+Paclitaxel	SOD+Paclitaxel	0.38748918	43.1746885	0	***
**H**	SOD+AAP+Paclitaxel	SOD+Sotorasib	-0.9481277	124.509305	0	***
**H**	SOD+AAP+Sotorasib	SOD+BPA	1.72188312	223.814939	0	***
**H**	SOD+AAP+Sotorasib	SOD+BPA+Paclitaxel	-0.1144805	17.7402001	2.60E-70	***
**H**	SOD+AAP+Sotorasib	SOD+BPA+Sotorasib	0.1372619	19.0879147	4.47E-81	***
**H**	SOD+AAP+Sotorasib	SOD+Paclitaxel	0.88693723	108.077278	0	***
**H**	SOD+AAP+Sotorasib	SOD+Sotorasib	-0.4486797	67.0458573	0	***
**H**	SOD+BPA	SOD+BPA+Paclitaxel	-1.8363636	244.943464	0	***
**H**	SOD+BPA	SOD+BPA+Sotorasib	-1.5846212	194.649754	0	***
**H**	SOD+BPA	SOD+Paclitaxel	-0.8349459	92.2551091	0	***
**H**	SOD+BPA	SOD+Sotorasib	-2.1705628	281.754431	0	***
**H**	SOD+BPA+Paclitaxel	SOD+BPA+Sotorasib	0.25174242	36.0626929	3.91E-283	***

H	SOD+BPA+Paclitaxel	SOD+Paclitaxel	1.00141775	124.821016	0	***
H	SOD+BPA+Paclitaxel	SOD+Sotorasib	-0.3341991	51.6890346	0	***
H	SOD+BPA+Sotorasib	SOD+Paclitaxel	0.74967532	86.893891	0	***
H	SOD+BPA+Sotorasib	SOD+Sotorasib	-0.5859416	81.3562701	0	***
H	SOD+Paclitaxel	SOD+Sotorasib	-1.3356169	162.557664	0	***
S	SOD	SOD+AAP	-2.7065476	183.626874	0	***
S	SOD	SOD+AAP+Paclitaxel	-1.4306277	105.063394	0	***
S	SOD	SOD+AAP+Sotorasib	-1.1792532	93.0589399	0	***
S	SOD	SOD+BPA	-2.3933333	171.001235	0	***
S	SOD	SOD+BPA+Paclitaxel	-0.3399567	20.4437817	1.24E-92	***
S	SOD	SOD+BPA+Sotorasib	-2.1001299	144.679105	0	***
S	SOD	SOD+Paclitaxel	-2.5514069	184.559614	0	***
S	SOD	SOD+Sotorasib	-0.8324134	69.1232075	0	***
S	SOD+AAP	SOD+AAP+Paclitaxel	1.27591991	79.1590908	0	***
S	SOD+AAP	SOD+AAP+Sotorasib	1.52729437	99.636339	0	***
S	SOD+AAP	SOD+BPA	0.31321429	19.0519392	9.40E-81	***
S	SOD+AAP	SOD+BPA+Paclitaxel	2.36659091	126.33635	0	***
S	SOD+AAP	SOD+BPA+Sotorasib	0.60641775	35.9151581	7.43E-281	***
S	SOD+AAP	SOD+Paclitaxel	0.15514069	9.5212844	1.74E-21	***
S	SOD+AAP	SOD+Sotorasib	1.8741342	126.524708	0	***
S	SOD+AAP+Paclitaxel	SOD+AAP+Sotorasib	0.25137446	17.6370991	1.66E-69	***
S	SOD+AAP+Paclitaxel	SOD+BPA	-0.9627056	62.3450554	0	***
S	SOD+AAP+Paclitaxel	SOD+BPA+Paclitaxel	1.090671	61.0588692	0	***
S	SOD+AAP+Paclitaxel	SOD+BPA+Sotorasib	-0.6695022	42.0694683	0	***
S	SOD+AAP+Paclitaxel	SOD+Paclitaxel	-1.1207792	73.320086	0	***
S	SOD+AAP+Paclitaxel	SOD+Sotorasib	0.59821429	43.6786674	0	***
S	SOD+AAP+Sotorasib	SOD+BPA	-1.2140801	83.0690848	0	***
S	SOD+AAP+Sotorasib	SOD+BPA+Paclitaxel	0.83929654	48.9289668	0	***
S	SOD+AAP+Sotorasib	SOD+BPA+Sotorasib	-0.9208766	60.9295196	0	***
S	SOD+AAP+Sotorasib	SOD+Paclitaxel	-1.3721537	94.9524044	0	***
S	SOD+AAP+Sotorasib	SOD+Sotorasib	0.34683983	27.1882868	3.38E-162	***
S	SOD+BPA	SOD+BPA+Paclitaxel	2.05337662	113.112905	0	***
S	SOD+BPA	SOD+BPA+Sotorasib	0.29320346	18.0545718	9.90E-73	***
S	SOD+BPA	SOD+Paclitaxel	-0.1580736	10.1168068	4.89E-24	***
S	SOD+BPA	SOD+Sotorasib	1.56091991	110.916979	0	***
S	SOD+BPA+Paclitaxel	SOD+BPA+Sotorasib	-1.7601732	94.8520844	0	***
S	SOD+BPA+Paclitaxel	SOD+Paclitaxel	-2.2114502	122.713222	0	***
S	SOD+BPA+Paclitaxel	SOD+Sotorasib	-0.4924567	29.4997231	2.55E-190	***
S	SOD+BPA+Sotorasib	SOD+Paclitaxel	-0.4512771	28.0433872	2.18E-172	***
S	SOD+BPA+Sotorasib	SOD+Sotorasib	1.26771645	86.8899586	0	***

**S**	SOD+Paclitaxel	SOD+Sotorasib	1.71899351	123.649798	0	***
**T**	SOD	SOD+AAP	1.48356061	103.265383	0	***
**T**	SOD	SOD+AAP+Paclitaxel	2.52975108	177.157498	0	***
**T**	SOD	SOD+AAP+Sotorasib	3.53874459	263.113296	0	***
**T**	SOD	SOD+BPA	2.84213203	184.030028	0	***
**T**	SOD	SOD+BPA+Paclitaxel	3.03899351	216.941366	0	***
**T**	SOD	SOD+BPA+Sotorasib	2.07088745	144.447974	0	***
**T**	SOD	SOD+Paclitaxel	2.03954545	138.907068	0	***
**T**	SOD	SOD+Sotorasib	2.75577922	204.647002	0	***
**T**	SOD+AAP	SOD+AAP+Paclitaxel	1.04619048	72.7167397	0	***
**T**	SOD+AAP	SOD+AAP+Sotorasib	2.05518398	151.521769	0	***
**T**	SOD+AAP	SOD+BPA	1.35857143	87.4054423	0	***
**T**	SOD+AAP	SOD+BPA+Paclitaxel	1.5554329	110.174054	0	***
**T**	SOD+AAP	SOD+BPA+Sotorasib	0.58732684	40.663271	0	***
**T**	SOD+AAP	SOD+Paclitaxel	0.55598485	37.5985425	1.91E-307	***
**T**	SOD+AAP	SOD+Sotorasib	1.27221861	93.6833417	0	***
**T**	SOD+AAP+Paclitaxel	SOD+AAP+Sotorasib	1.00899351	74.8976196	0	***
**T**	SOD+AAP+Paclitaxel	SOD+BPA	0.31238095	20.2016723	1.52E-90	***
**T**	SOD+AAP+Paclitaxel	SOD+BPA+Paclitaxel	0.50924242	36.2976899	8.29E-287	***
**T**	SOD+AAP+Paclitaxel	SOD+BPA+Sotorasib	-0.4588636	31.9602494	4.07E-223	***
**T**	SOD+AAP+Paclitaxel	SOD+Paclitaxel	-0.4902056	33.3403425	1.63E-242	***
**T**	SOD+AAP+Paclitaxel	SOD+Sotorasib	0.22602814	16.7575794	6.43E-63	***
**T**	SOD+AAP+Sotorasib	SOD+BPA	-0.6966126	47.3887821	0	***
**T**	SOD+AAP+Sotorasib	SOD+BPA+Paclitaxel	-0.4997511	37.9067134	0	***
**T**	SOD+AAP+Sotorasib	SOD+BPA+Sotorasib	-1.4678571	108.473645	0	***
**T**	SOD+AAP+Sotorasib	SOD+Paclitaxel	-1.4991991	107.869812	0	***
**T**	SOD+AAP+Sotorasib	SOD+Sotorasib	-0.7829654	62.1108811	0	***
**T**	SOD+BPA	SOD+BPA+Paclitaxel	0.19686147	12.9404008	3.00E-38	***
**T**	SOD+BPA	SOD+BPA+Sotorasib	-0.7712446	49.7074471	0	***
**T**	SOD+BPA	SOD+Paclitaxel	-0.8025866	50.6806363	0	***
**T**	SOD+BPA	SOD+Sotorasib	-0.0863528	5.8683337	4.54E-09	***
**T**	SOD+BPA+Paclitaxel	SOD+BPA+Sotorasib	-0.9681061	68.7208796	0	***
**T**	SOD+BPA+Paclitaxel	SOD+Paclitaxel	-0.9994481	69.2151697	0	***
**T**	SOD+BPA+Paclitaxel	SOD+Sotorasib	-0.2832143	21.4547406	7.48E-102	***
**T**	SOD+BPA+Sotorasib	SOD+Paclitaxel	-0.031342	2.12368067	0.0336989	*
**T**	SOD+BPA+Sotorasib	SOD+Sotorasib	0.68489177	50.5517557	0	***
**T**	SOD+Paclitaxel	SOD+Sotorasib	0.71623377	51.4750263	0	***
**P**	SOD	SOD+AAP	-0.0366991	4.86916069	1.22E-06	***
**P**	SOD	SOD+AAP+Paclitaxel	-0.9648052	101.055696	0	***
**P**	SOD	SOD+AAP+Sotorasib	0.02568182	3.39857782	0.00071745	***

P	SOD	SOD+BPA	0.09608225	13.5685957	8.20E-42	***
P	SOD	SOD+BPA+Paclitaxel	-0.9727922	117.372069	0	***
P	SOD	SOD+BPA+Sotorasib	0.31366883	44.538949	0	***
P	SOD	SOD+Paclitaxel	0.10143939	14.319223	2.33E-46	***
P	SOD	SOD+Sotorasib	-1.3087554	137.467853	0	***
P	SOD+AAP	SOD+AAP+Paclitaxel	-0.9281061	95.5195117	0	***
P	SOD+AAP	SOD+AAP+Sotorasib	0.06238095	8.0292256	1.11E-15	***
P	SOD+AAP	SOD+BPA	0.13278139	18.1701478	1.50E-73	***
P	SOD+AAP	SOD+BPA+Paclitaxel	-0.9360931	110.357197	0	***
P	SOD+AAP	SOD+BPA+Sotorasib	0.35036797	48.1921998	0	***
P	SOD+AAP	SOD+Paclitaxel	0.13813853	18.8959214	2.24E-79	***
P	SOD+AAP	SOD+Sotorasib	-1.2720563	131.274398	0	***
P	SOD+AAP+Paclitaxel	SOD+AAP+Sotorasib	0.99048701	101.780489	0	***
P	SOD+AAP+Paclitaxel	SOD+BPA	1.06088745	113.255193	0	***
P	SOD+AAP+Paclitaxel	SOD+BPA+Paclitaxel	-0.007987	0.77469372	0.43852226	NS
P	SOD+AAP+Paclitaxel	SOD+BPA+Sotorasib	1.27847403	136.910213	0	***
P	SOD+AAP+Paclitaxel	SOD+Paclitaxel	1.06624459	113.800294	0	***
P	SOD+AAP+Paclitaxel	SOD+Sotorasib	-0.3439502	30.3727075	1.06E-201	***
P	SOD+AAP+Sotorasib	SOD+BPA	0.07040043	9.60722807	8.82E-22	***
P	SOD+AAP+Sotorasib	SOD+BPA+Paclitaxel	-0.998474	117.470346	0	***
P	SOD+AAP+Sotorasib	SOD+BPA+Sotorasib	0.28798701	39.5015763	0	***
P	SOD+AAP+Sotorasib	SOD+Paclitaxel	0.07575758	10.3343163	6.06E-25	***
P	SOD+AAP+Sotorasib	SOD+Sotorasib	-1.3344372	137.4958	0	***
P	SOD+BPA	SOD+BPA+Paclitaxel	-1.0688745	132.285331	0	***
P	SOD+BPA	SOD+BPA+Sotorasib	0.21758658	32.014428	9.52E-224	***
P	SOD+BPA	SOD+Paclitaxel	0.00535714	0.78325622	0.43852226	NS
P	SOD+BPA	SOD+Sotorasib	-1.4048377	150.412523	0	***
P	SOD+BPA+Paclitaxel	SOD+BPA+Sotorasib	1.28646104	159.88394	0	***
P	SOD+BPA+Paclitaxel	SOD+Paclitaxel	1.0742316	132.906271	0	***
P	SOD+BPA+Paclitaxel	SOD+Sotorasib	-0.3359632	32.6651313	9.39E-233	***
P	SOD+BPA+Sotorasib	SOD+Paclitaxel	-0.2122294	31.2122471	7.82E-213	***
P	SOD+BPA+Sotorasib	SOD+Sotorasib	-1.6224242	174.255049	0	***
P	SOD+Paclitaxel	SOD+Sotorasib	-1.4101948	150.95034	0	***
~	SOD	SOD+AAP	-1.5969589	109.090081	0	***
~	SOD	SOD+AAP+Paclitaxel	-2.4788203	173.52992	0	***
~	SOD	SOD+AAP+Sotorasib	-3.8887229	265.827412	0	***
~	SOD	SOD+BPA	-1.9014286	124.214706	0	***
~	SOD	SOD+BPA+Paclitaxel	-2.4357576	164.911164	0	***
~	SOD	SOD+BPA+Sotorasib	-0.8866342	53.7806472	0	***
~	SOD	SOD+Paclitaxel	-1.8697619	125.453829	0	***

~	SOD	SOD+Sotorasib	-1.8858983	124.067033	0	***
~	SOD+AAP	SOD+AAP+Paclitaxel	-0.8818615	55.1644268	0	***
~	SOD+AAP	SOD+AAP+Sotorasib	-2.2917641	140.64879	0	***
~	SOD+AAP	SOD+BPA	-0.3044697	18.0092076	2.47E-72	***
~	SOD+AAP	SOD+BPA+Paclitaxel	-0.8387987	51.0800161	0	***
~	SOD+AAP	SOD+BPA+Sotorasib	0.71032468	39.5055585	0	***
~	SOD+AAP	SOD+Paclitaxel	-0.272803	16.4917601	5.52E-61	***
~	SOD+AAP	SOD+Sotorasib	-0.2889394	17.1889981	4.48E-66	***
~	SOD+AAP+Paclitaxel	SOD+AAP+Sotorasib	-1.4099026	88.2470957	0	***
~	SOD+AAP+Paclitaxel	SOD+BPA	0.57739177	34.7814386	1.38E-263	***
~	SOD+AAP+Paclitaxel	SOD+BPA+Paclitaxel	0.04306277	2.67365997	0.00818604	**
~	SOD+AAP+Paclitaxel	SOD+BPA+Sotorasib	1.59218615	89.9886908	0	***
~	SOD+AAP+Paclitaxel	SOD+Paclitaxel	0.60905844	37.528677	2.91E-306	***
~	SOD+AAP+Paclitaxel	SOD+Sotorasib	0.59292208	35.9303208	4.83E-281	***
~	SOD+AAP+Sotorasib	SOD+BPA	1.98729437	117.608453	0	***
~	SOD+AAP+Sotorasib	SOD+BPA+Paclitaxel	1.45296537	88.5294755	0	***
~	SOD+AAP+Sotorasib	SOD+BPA+Sotorasib	3.00208874	167.041529	0	***
~	SOD+AAP+Sotorasib	SOD+Paclitaxel	2.01896104	122.118548	0	***
~	SOD+AAP+Sotorasib	SOD+Sotorasib	2.00282468	119.210678	0	***
~	SOD+BPA	SOD+BPA+Paclitaxel	-0.534329	31.3940333	2.29E-215	***
~	SOD+BPA	SOD+BPA+Sotorasib	1.01479437	54.7682918	0	***
~	SOD+BPA	SOD+Paclitaxel	0.03166667	1.8479205	0.06841727	NS
~	SOD+BPA	SOD+Sotorasib	0.0155303	0.89280245	0.37196476	NS
~	SOD+BPA+Paclitaxel	SOD+BPA+Sotorasib	1.54912338	85.6467079	0	***
~	SOD+BPA+Paclitaxel	SOD+Paclitaxel	0.56599567	33.9773807	1.02E-251	***
~	SOD+BPA+Paclitaxel	SOD+Sotorasib	0.54985931	32.4900083	1.92E-230	***
~	SOD+BPA+Sotorasib	SOD+Paclitaxel	-0.9831277	54.0273736	0	***
~	SOD+BPA+Sotorasib	SOD+Sotorasib	-0.9992641	54.1882533	0	***
~	SOD+Paclitaxel	SOD+Sotorasib	-0.0161364	0.94691941	0.35350123	NS

SOD Complexes_Chain B

SS_Type	Group1	Group2	Mean_Diff	t_value	p_adj	Significance
E	SOD	SOD+AAP	0.03451299	2.91892868	0.00361345	**
E	SOD	SOD+AAP+Paclitaxel	1.87877706	99.5610508	0	***
E	SOD	SOD+AAP+Sotorasib	1.94028139	135.834617	0	***
E	SOD	SOD+BPA	0.195671	15.931469	5.18E-57	***
E	SOD	SOD+BPA+Paclitaxel	0.25166667	21.6345071	1.79E-103	***
E	SOD	SOD+BPA+Sotorasib	0.98359307	69.951135	0	***
E	SOD	SOD+Paclitaxel	0.83669913	64.3703093	0	***
E	SOD	SOD+Sotorasib	1.47465368	124.005018	0	***
E	SOD+AAP	SOD+AAP+Paclitaxel	1.84426407	92.3421019	0	***

**E**	SOD+AAP	SOD+AAP+Sotorasib	1.9057684	121.305047	0	***
**E**	SOD+AAP	SOD+BPA	0.16115801	11.5814301	5.98E-31	***
**E**	SOD+AAP	SOD+BPA+Paclitaxel	0.21715368	16.2716499	2.18E-59	***
**E**	SOD+AAP	SOD+BPA+Sotorasib	0.94908009	61.1991112	0	***
**E**	SOD+AAP	SOD+Paclitaxel	0.80218615	55.1284439	0	***
**E**	SOD+AAP	SOD+Sotorasib	1.44014069	106.110524	0	***
**E**	SOD+AAP+Paclitaxel	SOD+AAP+Sotorasib	0.06150433	2.85798641	0.0042642	**
**E**	SOD+AAP+Paclitaxel	SOD+BPA	-1.6831061	83.1298673	0	***
**E**	SOD+AAP+Paclitaxel	SOD+BPA+Paclitaxel	-1.6271104	81.9311504	0	***
**E**	SOD+AAP+Paclitaxel	SOD+BPA+Sotorasib	-0.895184	41.8842736	0	***
**E**	SOD+AAP+Paclitaxel	SOD+Paclitaxel	-1.0420779	50.3687283	0	***
**E**	SOD+AAP+Paclitaxel	SOD+Sotorasib	-0.4041234	20.1936141	2.16E-90	***
**E**	SOD+AAP+Sotorasib	SOD+BPA	-1.7446104	108.642855	0	***
**E**	SOD+AAP+Sotorasib	SOD+BPA+Paclitaxel	-1.6886147	108.472929	0	***
**E**	SOD+AAP+Sotorasib	SOD+BPA+Sotorasib	-0.9566883	54.8037613	0	***
**E**	SOD+AAP+Sotorasib	SOD+Paclitaxel	-1.1035823	66.4313	0	***
**E**	SOD+AAP+Sotorasib	SOD+Sotorasib	-0.4656277	29.5415111	6.06E-191	***
**E**	SOD+BPA	SOD+BPA+Paclitaxel	0.05599567	4.07149134	4.95E-05	***
**E**	SOD+BPA	SOD+BPA+Sotorasib	0.78792208	49.679266	0	***
**E**	SOD+BPA	SOD+Paclitaxel	0.64102814	42.9473118	0	***
**E**	SOD+BPA	SOD+Sotorasib	1.27898268	91.5320254	0	***
**E**	SOD+BPA+Paclitaxel	SOD+BPA+Sotorasib	0.73192641	47.64281	0	***
**E**	SOD+BPA+Paclitaxel	SOD+Paclitaxel	0.58503247	40.6377351	0	***
**E**	SOD+BPA+Paclitaxel	SOD+Sotorasib	1.22298701	91.2279557	0	***
**E**	SOD+BPA+Sotorasib	SOD+Paclitaxel	-0.1468939	8.94547536	4.10E-19	***
**E**	SOD+BPA+Sotorasib	SOD+Sotorasib	0.49106061	31.5591596	1.63E-217	***
**E**	SOD+Paclitaxel	SOD+Sotorasib	0.63795455	43.6759051	0	***
**G**	SOD	SOD+AAP	2.12601732	245.666898	0	***
**G**	SOD	SOD+AAP+Paclitaxel	2.11257576	243.532091	0	***
**G**	SOD	SOD+AAP+Sotorasib	1.56114719	162.875729	0	***
**G**	SOD	SOD+BPA	2.04145022	231.682262	0	***
**G**	SOD	SOD+BPA+Paclitaxel	2.34050866	282.607729	0	***
**G**	SOD	SOD+BPA+Sotorasib	0.80017316	84.5095326	0	***
**G**	SOD	SOD+Paclitaxel	0.79103896	80.1464293	0	***
**G**	SOD	SOD+Sotorasib	2.15668831	255.147316	0	***
**G**	SOD+AAP	SOD+AAP+Paclitaxel	-0.0134416	2.22393331	0.02690207	*
**G**	SOD+AAP	SOD+AAP+Sotorasib	-0.5648701	77.481469	0	***
**G**	SOD+AAP	SOD+BPA	-0.0845671	13.5553645	9.19E-42	***
**G**	SOD+AAP	SOD+BPA+Paclitaxel	0.21449134	39.2468876	0	***
**G**	SOD+AAP	SOD+BPA+Sotorasib	-1.3258442	185.781366	0	***

**G**	SOD+AAP	SOD+Paclitaxel	-1.3349784	174.250542	0	***
**G**	SOD+AAP	SOD+Sotorasib	0.030671	5.36127846	8.77E-08	***
**G**	SOD+AAP+Paclitaxel	SOD+AAP+Sotorasib	-0.5514286	75.3841755	0	***
**G**	SOD+AAP+Paclitaxel	SOD+BPA	-0.0711255	11.3487055	8.77E-30	***
**G**	SOD+AAP+Paclitaxel	SOD+BPA+Paclitaxel	0.2279329	41.4585581	0	***
**G**	SOD+AAP+Paclitaxel	SOD+BPA+Sotorasib	-1.3124026	183.254703	0	***
**G**	SOD+AAP+Paclitaxel	SOD+Paclitaxel	-1.3215368	171.972188	0	***
**G**	SOD+AAP+Paclitaxel	SOD+Sotorasib	0.04411255	7.66901087	1.91E-14	***
**G**	SOD+AAP+Sotorasib	SOD+BPA	0.48030303	64.2416643	0	***
**G**	SOD+AAP+Sotorasib	SOD+BPA+Paclitaxel	0.77936147	113.868716	0	***
**G**	SOD+AAP+Sotorasib	SOD+BPA+Sotorasib	-0.760974	92.3439826	0	***
**G**	SOD+AAP+Sotorasib	SOD+Paclitaxel	-0.7701082	88.528542	0	***
**G**	SOD+AAP+Sotorasib	SOD+Sotorasib	0.59554113	84.4714655	0	***
**G**	SOD+BPA	SOD+BPA+Paclitaxel	0.29905844	52.3645347	0	***
**G**	SOD+BPA	SOD+BPA+Sotorasib	-1.2412771	169.420504	0	***
**G**	SOD+BPA	SOD+Paclitaxel	-1.2504113	159.520312	0	***
**G**	SOD+BPA	SOD+Sotorasib	0.1152381	19.3475959	3.47E-83	***
**G**	SOD+BPA+Paclitaxel	SOD+BPA+Sotorasib	-1.5403355	230.578155	0	***
**G**	SOD+BPA+Paclitaxel	SOD+Paclitaxel	-1.5494697	214.070067	0	***
**G**	SOD+BPA+Paclitaxel	SOD+Sotorasib	-0.1838203	35.7598693	1.78E-278	***
**G**	SOD+BPA+Sotorasib	SOD+Paclitaxel	-0.0091342	1.06577308	0.2865284	NS
**G**	SOD+BPA+Sotorasib	SOD+Sotorasib	1.35651515	196.852051	0	***
**G**	SOD+Paclitaxel	SOD+Sotorasib	1.36564935	183.726246	0	***
**H**	SOD	SOD+AAP	-1.6985065	167.957178	0	***
**H**	SOD	SOD+AAP+Paclitaxel	-2.563474	329.476392	0	***
**H**	SOD	SOD+AAP+Sotorasib	-2.1985714	285.818577	0	***
**H**	SOD	SOD+BPA	-1.6890152	221.647197	0	***
**H**	SOD	SOD+BPA+Paclitaxel	-2.4665368	360.858009	0	***
**H**	SOD	SOD+BPA+Sotorasib	-0.5201732	64.1122725	0	***
**H**	SOD	SOD+Paclitaxel	-1.5260823	193.308325	0	***
**H**	SOD	SOD+Sotorasib	-4.4964069	595.636869	0	***
**H**	SOD+AAP	SOD+AAP+Paclitaxel	-0.8649675	81.3740789	0	***
**H**	SOD+AAP	SOD+AAP+Sotorasib	-0.5000649	47.3318093	0	***
**H**	SOD+AAP	SOD+BPA	0.00949134	0.902832	0.36661717	NS
**H**	SOD+AAP	SOD+BPA+Paclitaxel	-0.7680303	77.1232479	0	***
**H**	SOD+AAP	SOD+BPA+Sotorasib	1.17833333	108.346002	0	***
**H**	SOD+AAP	SOD+Paclitaxel	0.17242424	16.0943764	3.46E-58	***
**H**	SOD+AAP	SOD+Sotorasib	-2.7979004	267.45424	0	***
**H**	SOD+AAP+Paclitaxel	SOD+AAP+Sotorasib	0.3649026	43.6487813	0	***
**H**	SOD+AAP+Paclitaxel	SOD+BPA	0.87445887	105.434512	0	***

**H**	SOD+AAP+Paclitaxel	SOD+BPA+Paclitaxel	0.09693723	12.7904586	2.02E-37	***
**H**	SOD+AAP+Paclitaxel	SOD+BPA+Sotorasib	2.04330087	233.542596	0	***
**H**	SOD+AAP+Paclitaxel	SOD+Paclitaxel	1.03739177	121.381584	0	***
**H**	SOD+AAP+Paclitaxel	SOD+Sotorasib	-1.9329329	234.91213	0	***
**H**	SOD+AAP+Sotorasib	SOD+BPA	0.50955628	62.0568383	0	***
**H**	SOD+AAP+Sotorasib	SOD+BPA+Paclitaxel	-0.2679654	35.784831	7.55E-279	***
**H**	SOD+AAP+Sotorasib	SOD+BPA+Sotorasib	1.67839827	193.569764	0	***
**H**	SOD+AAP+Sotorasib	SOD+Paclitaxel	0.67248918	79.4316031	0	***
**H**	SOD+AAP+Sotorasib	SOD+Sotorasib	-2.2978355	282.118802	0	***
**H**	SOD+BPA	SOD+BPA+Paclitaxel	-0.7775216	104.867056	0	***
**H**	SOD+BPA	SOD+BPA+Sotorasib	1.16884199	135.800689	0	***
**H**	SOD+BPA	SOD+Paclitaxel	0.1629329	19.394489	1.26E-83	***
**H**	SOD+BPA	SOD+Sotorasib	-2.8073918	347.576495	0	***
**H**	SOD+BPA+Paclitaxel	SOD+BPA+Sotorasib	1.94636364	245.741351	0	***
**H**	SOD+BPA+Paclitaxel	SOD+Paclitaxel	0.94045455	122.201046	0	***
**H**	SOD+BPA+Paclitaxel	SOD+Sotorasib	-2.0298701	276.512396	0	***
**H**	SOD+BPA+Sotorasib	SOD+Paclitaxel	-1.0059091	113.652035	0	***
**H**	SOD+BPA+Sotorasib	SOD+Sotorasib	-3.9762338	465.388185	0	***
**H**	SOD+Paclitaxel	SOD+Sotorasib	-2.9703247	356.312246	0	***
**S**	SOD	SOD+AAP	-2.2144156	138.870011	0	***
**S**	SOD	SOD+AAP+Paclitaxel	-3.0565693	198.006587	0	***
**S**	SOD	SOD+AAP+Sotorasib	-2.5552814	156.535248	0	***
**S**	SOD	SOD+BPA	-1.7757576	119.251151	0	***
**S**	SOD	SOD+BPA+Paclitaxel	-0.7638203	52.1649755	0	***
**S**	SOD	SOD+BPA+Sotorasib	-2.3234848	156.163523	0	***
**S**	SOD	SOD+Paclitaxel	-1.7168939	121.816794	0	***
**S**	SOD	SOD+Sotorasib	-2.4177056	170.401433	0	***
**S**	SOD+AAP	SOD+AAP+Paclitaxel	-0.8421537	49.9674465	0	***
**S**	SOD+AAP	SOD+AAP+Sotorasib	-0.3408658	19.2903078	1.04E-82	***
**S**	SOD+AAP	SOD+BPA	0.43865801	26.8200279	6.99E-158	***
**S**	SOD+AAP	SOD+BPA+Paclitaxel	1.45059524	89.9332442	0	***
**S**	SOD+AAP	SOD+BPA+Sotorasib	-0.1090693	6.67320732	2.66E-11	***
**S**	SOD+AAP	SOD+Paclitaxel	0.49752165	31.823851	3.79E-221	***
**S**	SOD+AAP	SOD+Sotorasib	-0.20329	12.9330797	3.50E-38	***
**S**	SOD+AAP+Paclitaxel	SOD+AAP+Sotorasib	0.50128788	29.1240646	1.03E-185	***
**S**	SOD+AAP+Paclitaxel	SOD+BPA	1.28081169	80.7598101	0	***
**S**	SOD+AAP+Paclitaxel	SOD+BPA+Paclitaxel	2.29274892	146.72257	0	***
**S**	SOD+AAP+Paclitaxel	SOD+BPA+Sotorasib	0.73308442	46.257492	0	***
**S**	SOD+AAP+Paclitaxel	SOD+Paclitaxel	1.33967532	88.6390156	0	***
**S**	SOD+AAP+Paclitaxel	SOD+Sotorasib	0.63886364	42.0256868	0	***

**S**	SOD+AAP+Sotorasib	SOD+BPA	0.77952381	46.6099816	0	***
**S**	SOD+AAP+Sotorasib	SOD+BPA+Paclitaxel	1.79146104	108.550232	0	***
**S**	SOD+AAP+Sotorasib	SOD+BPA+Sotorasib	0.23179654	13.8689157	1.23E-43	***
**S**	SOD+AAP+Sotorasib	SOD+Paclitaxel	0.83838745	52.336992	0	***
**S**	SOD+AAP+Sotorasib	SOD+Sotorasib	0.13757576	8.54402231	1.43E-17	***
**S**	SOD+BPA	SOD+BPA+Paclitaxel	1.01193723	67.0712872	0	***
**S**	SOD+BPA	SOD+BPA+Sotorasib	-0.5477273	35.7599801	1.90E-278	***
**S**	SOD+BPA	SOD+Paclitaxel	0.05886364	4.04396199	5.26E-05	***
**S**	SOD+BPA	SOD+Sotorasib	-0.6419481	43.8273888	0	***
**S**	SOD+BPA+Paclitaxel	SOD+BPA+Sotorasib	-1.5596645	103.4584	0	***
**S**	SOD+BPA+Paclitaxel	SOD+Paclitaxel	-0.9530736	66.6410193	0	***
**S**	SOD+BPA+Paclitaxel	SOD+Sotorasib	-1.6538853	114.897226	0	***
**S**	SOD+BPA+Sotorasib	SOD+Paclitaxel	0.60659091	41.7093614	0	***
**S**	SOD+BPA+Sotorasib	SOD+Sotorasib	-0.0942208	6.43821348	1.25E-10	***
**S**	SOD+Paclitaxel	SOD+Sotorasib	-0.7008117	50.6501452	0	***
**T**	SOD	SOD+AAP	2.72404762	192.343332	0	***
**T**	SOD	SOD+AAP+Paclitaxel	4.09027056	284.791906	0	***
**T**	SOD	SOD+AAP+Sotorasib	3.05008658	210.257957	0	***
**T**	SOD	SOD+BPA	2.71386364	195.673717	0	***
**T**	SOD	SOD+BPA+Paclitaxel	2.29030303	174.545283	0	***
**T**	SOD	SOD+BPA+Sotorasib	3.3647619	240.128632	0	***
**T**	SOD	SOD+Paclitaxel	2.03974026	143.991335	0	***
**T**	SOD	SOD+Sotorasib	3.61045455	266.198393	0	***
**T**	SOD+AAP	SOD+AAP+Paclitaxel	1.36622294	93.2511505	0	***
**T**	SOD+AAP	SOD+AAP+Sotorasib	0.32603896	22.0411549	2.14E-107	***
**T**	SOD+AAP	SOD+BPA	-0.010184	0.71879814	0.47226671	NS
**T**	SOD+AAP	SOD+BPA+Paclitaxel	-0.4337446	32.2800713	1.66E-227	***
**T**	SOD+AAP	SOD+BPA+Sotorasib	0.64071429	44.7798776	0	***
**T**	SOD+AAP	SOD+Paclitaxel	-0.6843074	47.3296209	0	***
**T**	SOD+AAP	SOD+Sotorasib	0.88640693	63.9157696	0	***
**T**	SOD+AAP+Paclitaxel	SOD+AAP+Sotorasib	-1.040184	69.4207062	0	***
**T**	SOD+AAP+Paclitaxel	SOD+BPA	-1.3764069	95.7974815	0	***
**T**	SOD+AAP+Paclitaxel	SOD+BPA+Paclitaxel	-1.7999675	131.890526	0	***
**T**	SOD+AAP+Paclitaxel	SOD+BPA+Sotorasib	-0.7255087	50.0144945	0	***
**T**	SOD+AAP+Paclitaxel	SOD+Paclitaxel	-2.0505303	139.927955	0	***
**T**	SOD+AAP+Paclitaxel	SOD+Sotorasib	-0.479816	34.0961779	1.85E-253	***
**T**	SOD+AAP+Sotorasib	SOD+BPA	-0.3362229	23.1687488	2.00E-118	***
**T**	SOD+AAP+Sotorasib	SOD+BPA+Paclitaxel	-0.7597835	55.0607992	0	***
**T**	SOD+AAP+Sotorasib	SOD+BPA+Sotorasib	0.31467532	21.481532	3.95E-102	***
**T**	SOD+AAP+Sotorasib	SOD+Paclitaxel	-1.0103463	68.2877136	0	***

**T**	SOD+AAP+Sotorasib	SOD+Sotorasib	0.56036797	39.408539	0	***
**T**	SOD+BPA	SOD+BPA+Paclitaxel	-0.4235606	32.2648042	2.56E-227	***
**T**	SOD+BPA	SOD+BPA+Sotorasib	0.65089827	46.4329042	0	***
**T**	SOD+BPA	SOD+Paclitaxel	-0.6741234	47.569415	0	***
**T**	SOD+BPA	SOD+Sotorasib	0.89659091	66.0768141	0	***
**T**	SOD+BPA+Paclitaxel	SOD+BPA+Sotorasib	1.07445887	80.9164222	0	***
**T**	SOD+BPA+Paclitaxel	SOD+Paclitaxel	-0.2505628	18.6425285	1.99E-77	***
**T**	SOD+BPA+Paclitaxel	SOD+Sotorasib	1.32015152	103.107884	0	***
**T**	SOD+BPA+Sotorasib	SOD+Paclitaxel	-1.3250216	92.5854118	0	***
**T**	SOD+BPA+Sotorasib	SOD+Sotorasib	0.24569264	17.9140889	1.17E-71	***
**T**	SOD+Paclitaxel	SOD+Sotorasib	1.57071429	113.23138	0	***
**P**	SOD	SOD+AAP	-0.3902056	49.1682318	0	***
**P**	SOD	SOD+AAP+Paclitaxel	-0.1743506	22.7187384	6.57E-114	***
**P**	SOD	SOD+AAP+Sotorasib	0.16670996	22.764459	2.41E-114	***
**P**	SOD	SOD+BPA	-0.0891991	11.7184057	1.20E-31	***
**P**	SOD	SOD+BPA+Paclitaxel	0.33313853	49.4682375	0	***
**P**	SOD	SOD+BPA+Sotorasib	0.10463203	13.6303346	3.41E-42	***
**P**	SOD	SOD+Paclitaxel	-0.2929113	37.5237338	5.69E-306	***
**P**	SOD	SOD+Sotorasib	0.15916667	22.8710181	2.24E-115	***
**P**	SOD+AAP	SOD+AAP+Paclitaxel	0.21585498	26.446466	1.82E-153	***
**P**	SOD+AAP	SOD+AAP+Sotorasib	0.55691558	71.1005289	0	***
**P**	SOD+AAP	SOD+BPA	0.30100649	37.146209	5.99E-300	***
**P**	SOD+AAP	SOD+BPA+Paclitaxel	0.72334416	99.2891254	0	***
**P**	SOD+AAP	SOD+BPA+Sotorasib	0.49483766	60.6126534	0	***
**P**	SOD+AAP	SOD+Paclitaxel	0.09729437	11.7421227	9.37E-32	***
**P**	SOD+AAP	SOD+Sotorasib	0.54937229	73.311812	0	***
**P**	SOD+AAP+Paclitaxel	SOD+AAP+Sotorasib	0.34106061	45.0697584	0	***
**P**	SOD+AAP+Paclitaxel	SOD+BPA	0.08515152	10.8514097	2.21E-27	***
**P**	SOD+AAP+Paclitaxel	SOD+BPA+Paclitaxel	0.50748918	72.5077663	0	***
**P**	SOD+AAP+Paclitaxel	SOD+BPA+Sotorasib	0.27898268	35.2711578	8.51E-271	***
**P**	SOD+AAP+Paclitaxel	SOD+Paclitaxel	-0.1185606	14.754584	3.94E-49	***
**P**	SOD+AAP+Paclitaxel	SOD+Sotorasib	0.33351732	46.2205561	0	***
**P**	SOD+AAP+Sotorasib	SOD+BPA	-0.2559091	34.1026736	2.14E-253	***
**P**	SOD+AAP+Sotorasib	SOD+BPA+Paclitaxel	0.16642857	25.1695608	3.08E-139	***
**P**	SOD+AAP+Sotorasib	SOD+BPA+Sotorasib	-0.0620779	8.20103505	2.55E-16	***
**P**	SOD+AAP+Sotorasib	SOD+Paclitaxel	-0.4596212	59.6837911	0	***
**P**	SOD+AAP+Sotorasib	SOD+Sotorasib	-0.0075433	1.10262103	0.27019401	NS
**P**	SOD+BPA	SOD+BPA+Paclitaxel	0.42233766	60.9381584	0	***
**P**	SOD+BPA	SOD+BPA+Sotorasib	0.19383117	24.6947011	3.81E-134	***
**P**	SOD+BPA	SOD+Paclitaxel	-0.2037121	25.5409244	2.63E-143	***

P	SOD+BPA	SOD+Sotorasib	0.2483658	34.7396387	9.03E-263	***
P	SOD+BPA+Paclitaxel	SOD+BPA+Sotorasib	-0.2285065	32.6372437	2.76E-232	***
P	SOD+BPA+Paclitaxel	SOD+Paclitaxel	-0.6260498	87.6418041	0	***
P	SOD+BPA+Paclitaxel	SOD+Sotorasib	-0.1739719	28.0291985	4.47E-172	***
P	SOD+BPA+Sotorasib	SOD+Paclitaxel	-0.3975433	49.46097	0	***
P	SOD+BPA+Sotorasib	SOD+Sotorasib	0.05453463	7.55535367	4.33E-14	***
P	SOD+Paclitaxel	SOD+Sotorasib	0.45207792	61.4594516	0	***
~	SOD	SOD+AAP	0.52769481	35.6351834	1.65E-276	***
~	SOD	SOD+AAP+Paclitaxel	-1.6828139	111.174022	0	***
~	SOD	SOD+AAP+Sotorasib	-1.2405628	83.3212735	0	***
~	SOD	SOD+BPA	-0.2608766	17.3617797	2.20E-67	***
~	SOD	SOD+BPA+Paclitaxel	-1.0269481	75.0212968	0	***
~	SOD	SOD+BPA+Sotorasib	-1.107197	66.2013924	0	***
~	SOD	SOD+Paclitaxel	0.77984848	48.8241132	0	***
~	SOD	SOD+Sotorasib	-0.5385606	37.3513411	2.00E-303	***
~	SOD+AAP	SOD+AAP+Paclitaxel	-2.2105087	137.824504	0	***
~	SOD+AAP	SOD+AAP+Sotorasib	-1.7682576	111.880501	0	***
~	SOD+AAP	SOD+BPA	-0.7885714	49.4898284	0	***
~	SOD+AAP	SOD+BPA+Paclitaxel	-1.5546429	105.903542	0	***
~	SOD+AAP	SOD+BPA+Sotorasib	-1.6348918	93.1824126	0	***
~	SOD+AAP	SOD+Paclitaxel	0.25215368	14.9826602	1.16E-50	***
~	SOD+AAP	SOD+Sotorasib	-1.0662554	69.4050141	0	***
~	SOD+AAP+Paclitaxel	SOD+AAP+Sotorasib	0.44225108	27.4467251	2.97E-165	***
~	SOD+AAP+Paclitaxel	SOD+BPA	1.42193723	87.5590297	0	***
~	SOD+AAP+Paclitaxel	SOD+BPA+Paclitaxel	0.6558658	43.6920166	0	***
~	SOD+AAP+Paclitaxel	SOD+BPA+Sotorasib	0.57561688	32.2959369	1.00E-227	***
~	SOD+AAP+Paclitaxel	SOD+Paclitaxel	2.46266234	143.851667	0	***
~	SOD+AAP+Paclitaxel	SOD+Sotorasib	1.14425325	72.9767849	0	***
~	SOD+AAP+Sotorasib	SOD+BPA	0.97968615	61.1959822	0	***
~	SOD+AAP+Sotorasib	SOD+BPA+Paclitaxel	0.21361472	14.4714072	2.14E-47	***
~	SOD+AAP+Sotorasib	SOD+BPA+Sotorasib	0.1333658	7.57192547	3.81E-14	***
~	SOD+AAP+Sotorasib	SOD+Paclitaxel	2.02041126	119.545931	0	***
~	SOD+AAP+Sotorasib	SOD+Sotorasib	0.70200216	45.4648138	0	***
~	SOD+BPA	SOD+BPA+Paclitaxel	-0.7660714	51.4164706	0	***
~	SOD+BPA	SOD+BPA+Sotorasib	-0.8463203	47.7360606	0	***
~	SOD+BPA	SOD+Paclitaxel	1.04072511	61.1416801	0	***
~	SOD+BPA	SOD+Sotorasib	-0.277684	17.8314445	5.79E-71	***
~	SOD+BPA+Paclitaxel	SOD+BPA+Sotorasib	-0.0802489	4.8310558	1.36E-06	***
~	SOD+BPA+Paclitaxel	SOD+Paclitaxel	1.80679654	113.967632	0	***
~	SOD+BPA+Paclitaxel	SOD+Sotorasib	0.48838745	34.1844712	9.58E-255	***

~	SOD+BPA+Sotorasib	SOD+Paclitaxel	1.88704545	101.79161	0	***
~	SOD+BPA+Sotorasib	SOD+Sotorasib	0.56863636	33.0264368	5.94E-238	***
~	SOD+Paclitaxel	SOD+Sotorasib	-1.3184091	79.9615289	0	***

p < 0.001 → Highly Significant (***), p < 0.05 → Significant (**), and p > 0.05 → Not Significant (NS)

Sup. table 7. Pairwise Statistical Comparisons of the Distances Between Pollutants and Important Residues in Pro-GRP and SOD Complexes

Pro-GRP complexes						
**Group1**	**Group2**	**Mean Diff**	**t-value**	**df**	**p_adj**	**Significance**
Pro-GRP+AAP+Paclitaxel	Pro-GRP+BPA+Paclitaxel	-0.8206	49.34	5528.34	0	***
Pro-GRP+AAP+Sotorasib	Pro-GRP+BPA+Paclitaxel	-0.8955	53.19	5787.93	0	***
Pro-GRP+AAP+Sotorasib	Pro-GRP+BPA+Sotorasib	-0.2638	40.85	9998	0	***
Pro-GRP+BPA	Pro-GRP+BPA+Paclitaxel	-0.7786	45.93	5933.26	0	***
Pro-GRP+BPA+Paclitaxel	Pro-GRP+BPA+Sotorasib	0.6317	37.52	5788.2	8.56E-275	***
Pro-GRP+AAP	Pro-GRP+BPA+Paclitaxel	-0.6427	35.06	7591.54	1.46E-248	***
Pro-GRP+AAP+Paclitaxel	Pro-GRP+BPA+Sotorasib	-0.1889	32.02	9618.05	1.24E-212	***
Pro-GRP+AAP	Pro-GRP+AAP+Sotorasib	0.2528	26.04	7626.7	4.43E-142	***
Pro-GRP+BPA	Pro-GRP+BPA+Sotorasib	-0.1469	21.75	9925.55	2.49E-101	***
Pro-GRP+AAP	Pro-GRP+AAP+Paclitaxel	0.1779	19.03	6831.61	1.50E-77	***
Pro-GRP+AAP+Sotorasib	Pro-GRP+BPA	-0.1169	17.31	9925.25	5.57E-65	***
Pro-GRP+AAP	Pro-GRP+BPA	0.1359	13.72	8026.22	3.65E-41	***
Pro-GRP+AAP+Paclitaxel	Pro-GRP+AAP+Sotorasib	0.0749	12.7	9618.68	1.69E-35	***
Pro-GRP+AAP+Paclitaxel	Pro-GRP+BPA	-0.042	6.75	9273.79	2.40E-10	***
Pro-GRP+AAP	Pro-GRP+BPA+Sotorasib	-0.011	1.13	7627.5	1.00E+00	ns

SOD Complexes (Chain A)						
**Group1**	**Group2**	**Mean_Diff**	**t_value**	**df**	**p_adj**	**Significance**
SOD+AAP	SOD+AAP+Paclitaxel	0.0939	39.72	118592.48	0	***
SOD+AAP	SOD+BPA	0.2396	132.04	67471.36	0	***
SOD+AAP	SOD+BPA+Paclitaxel	0.2132	120.08	61983.74	0	***
SOD+AAP	SOD+BPA+Sotorasib	0.2252	126.02	63552.12	0	***
SOD+AAP+Paclitaxel	SOD+AAP+Sotorasib	-0.1388	74.66	100390.93	0	***
SOD+AAP+Paclitaxel	SOD+BPA	0.1457	88.92	69277.27	0	***
SOD+AAP+Paclitaxel	SOD+BPA+Paclitaxel	0.1193	74.79	62468.3	0	***
SOD+AAP+Paclitaxel	SOD+BPA+Sotorasib	0.1312	81.64	64418.13	0	***
SOD+AAP+Sotorasib	SOD+BPA	0.2845	264.37	83180.94	0	***
SOD+AAP+Sotorasib	SOD+BPA+Paclitaxel	0.258	256.05	66363.92	0	***
SOD+AAP+Sotorasib	SOD+BPA+Sotorasib	0.27	262.73	71329.88	0	***
SOD+BPA	SOD+BPA+Paclitaxel	-0.0265	53.46	89677.74	0	***
SOD+BPA+Paclitaxel	SOD+BPA+Sotorasib	0.012	31.64	111071.65	1.47E-217	***
SOD+BPA	SOD+BPA+Sotorasib	-0.0145	27.12	106447.34	3.47E-160	***
SOD+AAP	SOD+AAP+Sotorasib	-0.0449	22.25	94029.87	3.17E-108	***

SOD Complexes (ChainB)						
SOD+AAP	SOD+AAP+Paclitaxel	-0.824	330.04	82,785.63	0	***
SOD+AAP	SOD+AAP+Sotorasib	-0.456	181.04	84,819.97	0	***
SOD+AAP	SOD+BPA	-0.421	176.57	70,989.95	0	***
SOD+AAP	SOD+BPA+Paclitaxel	-0.191	83.33	61,242.15	0	***
SOD+AAP	SOD+BPA+Sotorasib	-0.322	139.31	63,100.68	0	***

SOD+AAP+Paclitaxel	SOD+AAP+Sotorasib	0.368	250.96	119,738.59	0	***
SOD+AAP+Paclitaxel	SOD+BPA	0.403	328.1	106,082.62	0	***
SOD+AAP+Paclitaxel	SOD+BPA+Paclitaxel	0.633	608.69	66,283.72	0	***
SOD+AAP+Paclitaxel	SOD+BPA+Sotorasib	0.502	465.79	75,465.68	0	***
SOD+AAP+Sotorasib	SOD+BPA+Paclitaxel	0.265	243.37	65,727.37	0	***
SOD+AAP+Sotorasib	SOD+BPA+Sotorasib	0.134	119	74,130.50	0	***
SOD+BPA	SOD+BPA+Paclitaxel	0.23	314.43	73,290.99	0	***
SOD+BPA	SOD+BPA+Sotorasib	0.099	126.35	91,155.98	0	***
SOD+BPA+Paclitaxel	SOD+BPA+Sotorasib	-0.131	301.25	101,420.65	0	***
SOD+AAP+Sotorasib	SOD+BPA	0.035	27.25	103,307.27	7.04E-163	***

p < 0.001 → Highly Significant (***)

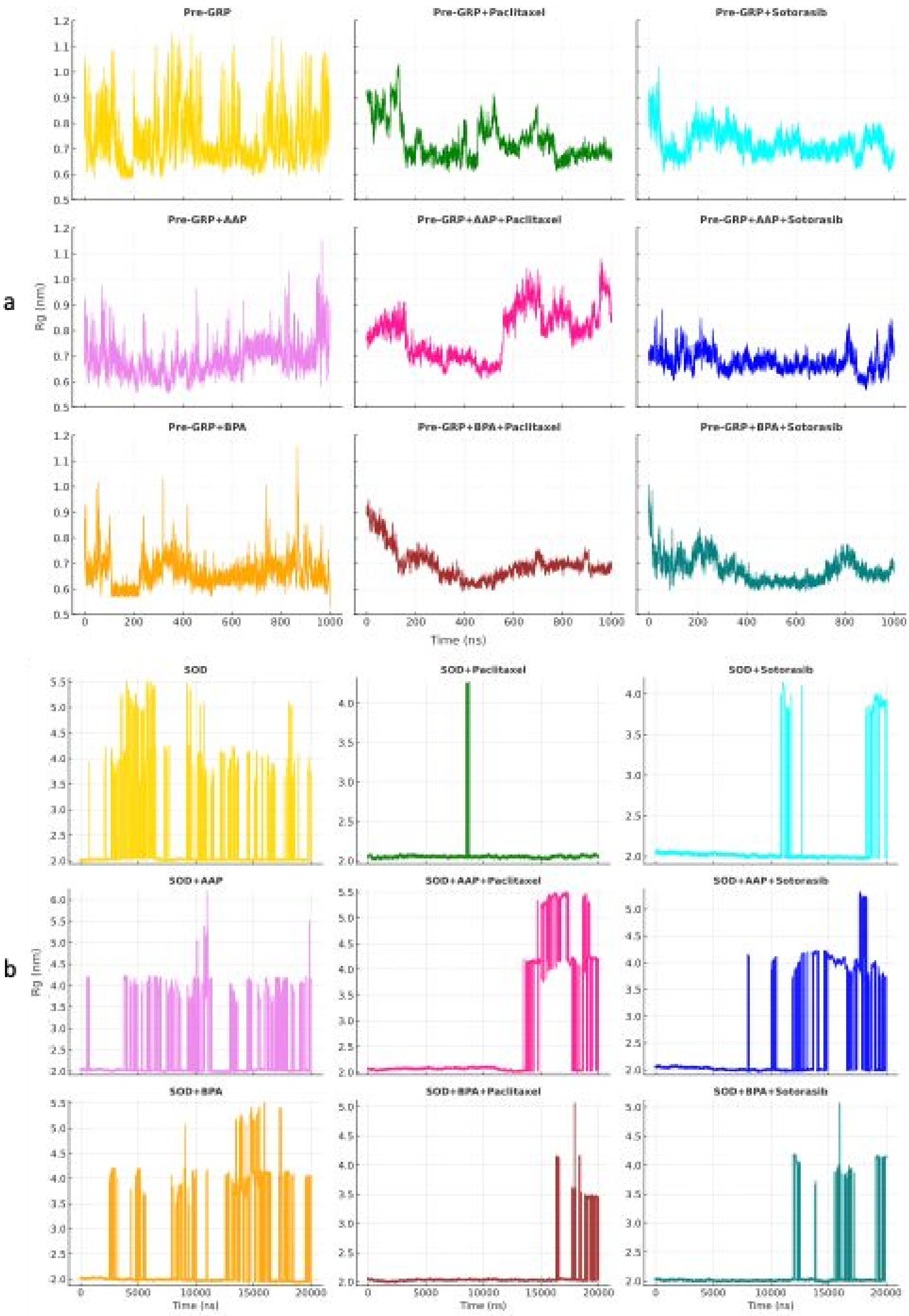

Sup. Figure 3. Rg of proteins (a: Pro-GRP and b: SOD) during a 1000 ns simulation.

Sup. table 8. Pairwise Statistical Comparisons of Pro-GRP and SOD Complexes binding affinity between pollutants and proteins

Pro-GRP complexes						
**Group1**	**Group2**	**Mean Diff**	**t-value**	**p-value**	**p_adj**	**Significance**
Pro-GRP+AAP+Sotorasib	Pro-GRP+BPA+Paclitaxel	-27.85	17.02	2.12E-48	3.18E-47	***
Pro-GRP+AAP+Sotorasib	Pro-GRP+BPA	-27.25	16.56	1.41E-46	2.12E-45	***
Pro-GRP+AAP+Sotorasib	Pro-GRP+BPA+Sotorasib	-23.39	13.00	1.91E-32	2.86E-31	***
Pro-GRP+AAP+Paclitaxel	Pro-GRP+AAP+Sotorasib	16.42	10.72	3.20E-23	4.80E-22	***
Pro-GRP+AAP+Paclitaxel	Pro-GRP+BPA+Paclitaxel	-11.43	8.98	1.22E-17	1.82E-16	***
Pro-GRP+AAP+Paclitaxel	Pro-GRP+BPA	-10.84	8.44	6.82E-16	1.02E-14	***
Pro-GRP+AAP	Pro-GRP+AAP+Sotorasib	15.30	8.31	1.57E-15	2.35E-14	***
Pro-GRP+AAP	Pro-GRP+BPA+Paclitaxel	-12.55	7.69	1.37E-13	2.05E-12	***
Pro-GRP+AAP	Pro-GRP+BPA	-11.96	7.28	1.96E-12	2.94E-11	***
Pro-GRP+AAP+Paclitaxel	Pro-GRP+BPA+Sotorasib	-6.97	4.73	3.34E-06	5.01E-05	***
Pro-GRP+AAP	Pro-GRP+BPA+Sotorasib	-8.09	4.51	8.63E-06	1.29E-04	***
Pro-GRP+BPA+Paclitaxel	Pro-GRP+BPA+Sotorasib	4.46	2.81	5.18E-03	7.77E-02	*
Pro-GRP+BPA	Pro-GRP+BPA+Sotorasib	3.86	2.42	1.58E-02	2.38E-01	*
Pro-GRP+AAP	Pro-GRP+AAP+Paclitaxel	-1.12	0.73	4.64E-01	1.00E+00	NS
Pro-GRP+BPA	Pro-GRP+BPA+Paclitaxel	-0.59	0.42	6.74E-01	1.00E+00	NS

SOD Complexes						
Group1	**Group2**	**Mean_Diff**	**t_value**	**p_value**	**p_adj**	**Significance**
SOD+AAP	SOD+BPA+Sotorasib	-31.541895	24.2062097	1.25E-79	1.88E-78	***
SOD+AAP+Paclitaxel	SOD+BPA+Sotorasib	-25.326635	22.0756559	6.72E-71	1.01E-69	***
SOD+AAP	SOD+BPA	-26.35335	15.3490867	3.37E-41	5.06E-40	***
SOD+AAP	SOD+AAP+Sotorasib	-30.68845	14.1487174	4.37E-35	6.56E-34	***
SOD+BPA	SOD+AAP+Paclitaxel	20.13809	12.5712461	1.48E-29	2.22E-28	***
SOD+AAP	SOD+BPA+Paclitaxel	-23.699915	11.652756	2.75E-26	4.13E-25	***
SOD+AAP+Paclitaxel	SOD+AAP+Sotorasib	-24.47319	11.7708496	6.11E-26	9.16E-25	***
SOD+AAP+Paclitaxel	SOD+BPA+Paclitaxel	-17.484655	9.0232519	3.43E-17	5.15E-16	***
SOD+AAP	SOD+AAP+Paclitaxel	-6.21526	4.9556844	1.09E-06	1.64E-05	***
SOD+BPA+Paclitaxel	SOD+BPA+Sotorasib	-7.84198	3.9812588	8.70E-05	1.31E-03	***
SOD+BPA	SOD+BPA+Sotorasib	-5.188545	3.1628422	1.71E-03	2.56E-02	**
SOD+AAP+Sotorasib	SOD+BPA+Paclitaxel	6.988535	2.663116	8.06E-03	1.21E-01	*
SOD+BPA	SOD+AAP+Sotorasib	-4.3351	1.816136	7.02E-02	1.00E+00	NS
SOD+BPA	SOD+BPA+Paclitaxel	2.653435	1.1715509	2.42E-01	1.00E+00	NS
SOD+AAP+Sotorasib	SOD+BPA+Sotorasib	-0.853445	0.4046715	6.86E-01	1.00E+00	NS

p < 0.001 → Highly Significant (***), p < 0.05 → Significant (**), and p > 0.05 → Not Significant (NS)

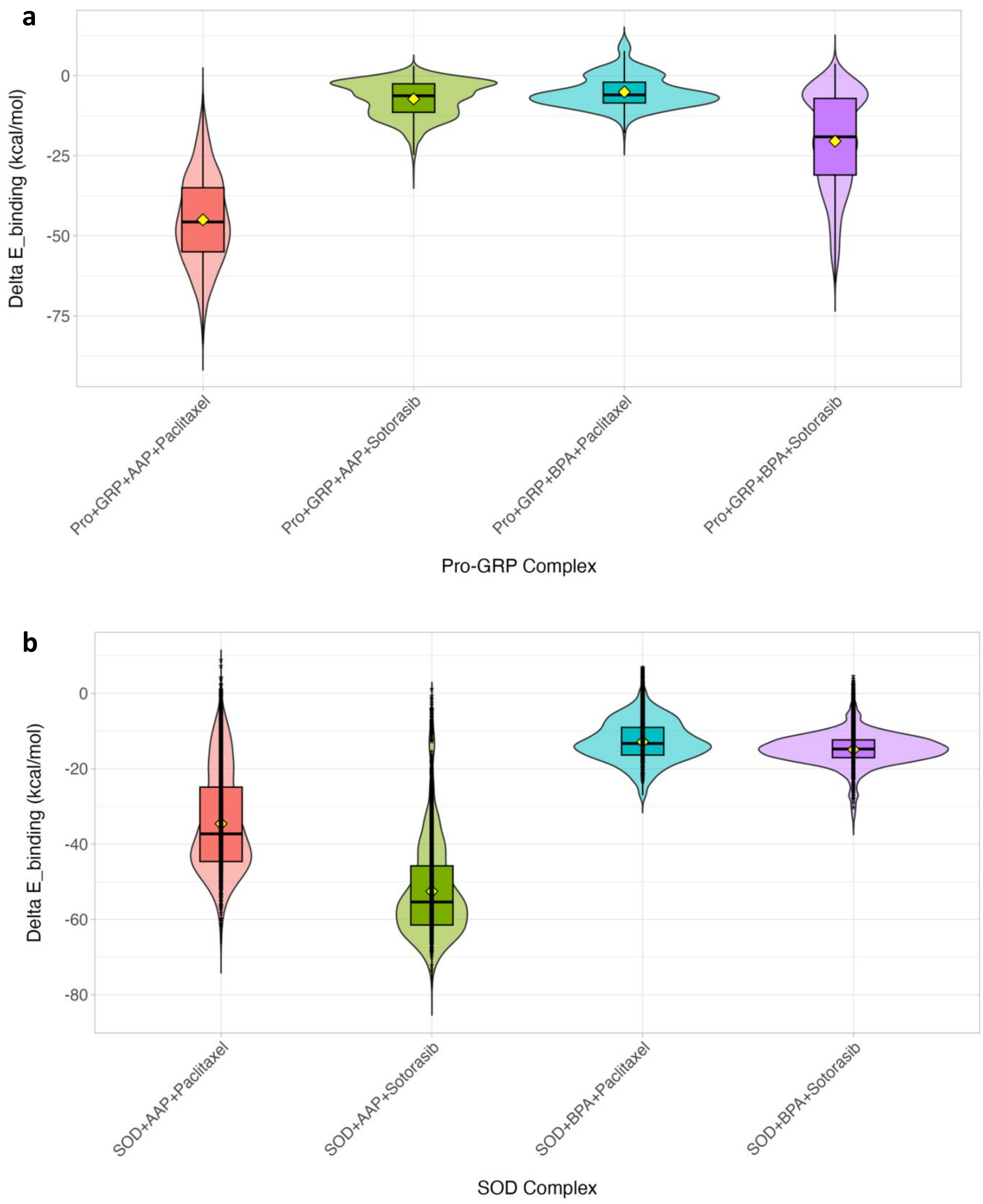

Sup. Figure 4. Binding affinity between anticancer drugs and pollutants in Pro-GRP and SOD complexes. Violin plots represent the binding energy (ΔE_binding) distribution of anticancer drugs (Paclitaxel and Sotorasib) in combination with pollutants (AAP and BPA) for Pro-GRP (a) and SOD (b) complexes. The yellow diamonds indicate mean binding energy values for each condition. Statistical analysis (Sup. Table 8) reveals significant differences in binding affinity across different drug-pollutant combinations, with Paclitaxel generally showing stronger binding than Sotorasib. In SOD complexes, Paclitaxel exhibits a significantly higher affinity when combined with AAP, whereas Sotorasib has a greater impact in BPA-containing complexes. These variations in binding affinity may influence the structural stability and functional interactions of the proteins.

Sup. table 9. Pairwise Statistical Comparisons of Pro-GRP and SOD Complexes binding affinity between pollutants and anticancers

Pro-GRP complexes					
**Group1**	**Group2**	**MeanDiff**	**t_value**	**p_value**	**p_value_adj**
Pro.GRP.AAP.Paclitaxel	Pro.GRP.AAP.Sotorasib	-37.704274	113.12873	0.00E+00	0.00E+00
Pro.GRP.AAP.Paclitaxel	Pro.GRP.BPA.Paclitaxel	-39.8999	121.52557	0.00E+00	0.00E+00
Pro.GRP.AAP.Paclitaxel	Pro.GRP.BPA.Sotorasib	-24.539031	53.68324	0.00E+00	0.00E+00
Pro.GRP.BPA.Paclitaxel	Pro.GRP.BPA.Sotorasib	15.360869	43.22833	1.75E-303	1.05E-302
Pro.GRP.AAP.Sotorasib	Pro.GRP.BPA.Sotorasib	13.165243	36.57706	9.18E-236	5.51E-235
Pro.GRP.AAP.Sotorasib	Pro.GRP.BPA.Paclitaxel	-2.195625	13.0285	5.08E-38	3.05E-37
**SOD Complexes**					
**Group1**	**Group2**	**MeanDiff**	**t_value**	**p_value**	**p_value_adj**
SOD+AAP+Paclitaxel	SOD+AAP+Sotorasib	17.950729	44.20574	0.00E+00	0.00E+00
SOD+AAP+Paclitaxel	SOD+BPA+Paclitaxel	-21.711153	68.40076	0.00E+00	0.00E+00
SOD+AAP+Paclitaxel	SOD+BPA+Sotorasib	-19.665152	63.65527	0.00E+00	0.00E+00
SOD+AAP+Sotorasib	SOD+BPA+Paclitaxel	-39.661883	130.15941	0.00E+00	0.00E+00
SOD+AAP+Sotorasib	SOD+BPA+Sotorasib	-37.615881	127.13427	0.00E+00	0.00E+00
SOD+BPA+Paclitaxel	SOD+BPA+Sotorasib	2.046002	13.37596	6.58E-40	3.95E-39
